# Fast, Efficient, and Versatile Synthesis of 6-amino-5-carboxamidouracils as Precursors for 8-Substituted Xanthines

**DOI:** 10.3389/fchem.2019.00056

**Published:** 2019-02-18

**Authors:** Daniel Marx, Lukas M. Wingen, Gregor Schnakenburg, Christa E. Müller, Matthias S. Scholz

**Affiliations:** ^1^Pharmaceutical Chemistry 1, Pharmaceutical Institute, University of Bonn, Bonn, Germany; ^2^Department of Chemistry, Institute of Inorganic Chemistry, University of Bonn, Bonn, Germany

**Keywords:** amide, COMU, purine, uracil, xanthine, X-ray crystal structure

## Abstract

Substituted xanthine derivatives are important bioactive molecules. Herein we report on a new, practical synthesis of 6-amino-5-carboxamidouracils, the main building blocks for the preparation of 8-substituted xanthines, by condensation of 5,6-diaminouracil derivatives and various carboxylic acids using the recently developed non-hazardous coupling reagent COMU (1-[(1-(cyano-2-ethoxy-2-oxoethylideneaminooxy)dimethylaminomorpholinomethylene)]methanaminium hexafluorophosphate). Optimized reaction conditions led to the precipitation of pure products after only 5 to 10 min of reaction time. The method tolerates a variety of substituted 5,6-diaminouracil and carboxylic acid derivatives as starting compounds resulting in most cases in more than 80% isolated yield. Regioselectivity of the reaction yielding only the 5-carboxamido-, but not the 6-carboxamidouracil derivatives, was unambiguously confirmed by single X-ray crystallography and multidimensional NMR experiments. The described method represents a convenient, fast access to direct precursors of 8-substituted xanthines under mild conditions without the necessity of hazardous coupling or chlorinating reagents.

## Introduction

**Figure 1:**
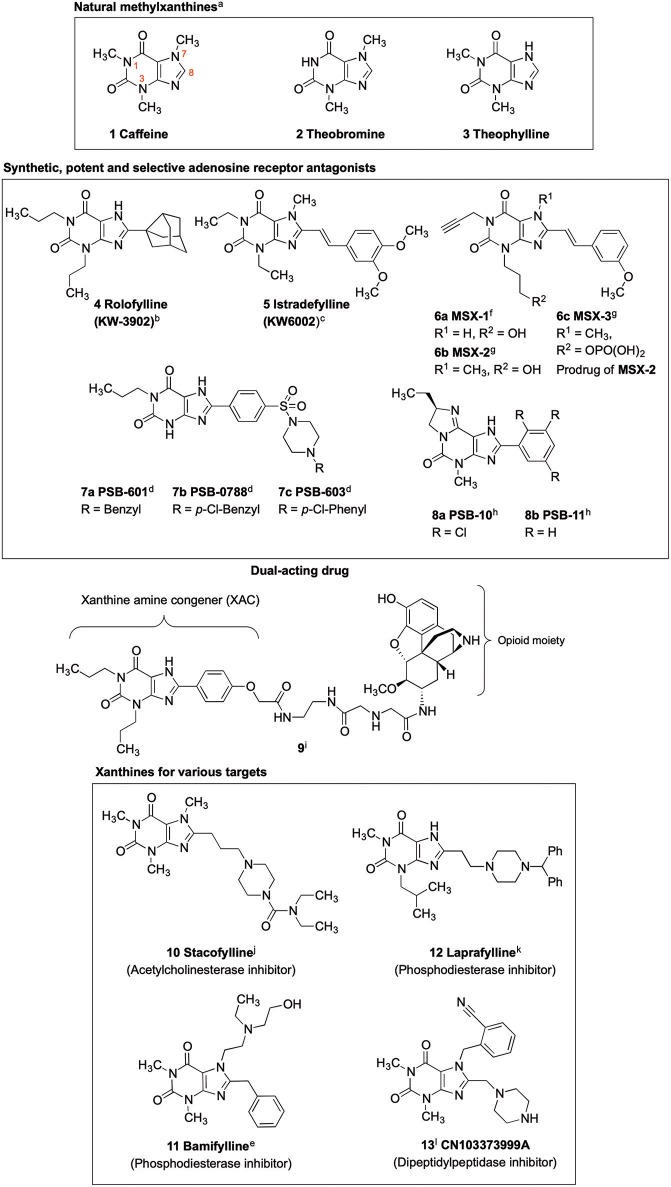
Xanthine-based drugs. ^a^(Franco et al., 2013); ^b^(Slawsky and Givertz, 2009); ^c^(Park and Stacy, 2012); ^d^(Borrmann et al., 2009); ^e^(Alciato et al., 1990); ^f^(Sauer et al., 2000); ^g^(Hockemeyer et al., 2004); ^h^(Ozola et al., 2003); ^i^(Jacobson, 2009); ^j^(Gallagher, 2004); ^k^(Baraldi et al., 2007); ^l^(Costante et al., 2015).

Xanthines are privileged structures in medicinal chemistry (Jacobson et al., 1993; Scammells et al., 1994; Kim et al., 2000; Baraldi et al., 2004; Müller and Jacobson, 2011). The methylxanthines caffeine (compound **1**, Figure 1), theobromine (**2**) and theophylline (**3**) are frequently consumed and therapeutically applied natural products (Franco et al., 2013). The biological activities of **1** and **2**, including central nervous system stimulatory, diuretic and antiasthmatic effects, are due to their blockade of adenosine receptors (ARs). The ARs, which belong to the family of G protein-coupled receptors (GPCRs), are (potential) drug targets for several diseases, in particular for heart and brain diseases (Baraldi et al., 2008; Müller and Jacobson, 2011; Chen et al., 2013). Recent findings point toward a great potential of A_2A_ and A_2B_ AR antagonists in immuno-oncology (Leone et al., 2015; Müller et al., 2018).

Caffeine and theophylline are weak, non-selective AR antagonists (Nieber, 2017; Oñatibia-Astibia et al., 2017). Replacing the hydrogen atom at *C*8 by a larger residue, in combination with suitable substituents at the xanthine nitrogen atoms, may result in highly potent and subtype-selective AR antagonists (Baraldi et al., 2007; Müller and Jacobson, 2011). Inspired by the natural methylxanthines, several drugs have been developed, which were designated by the suffix “fylline” (Figure 1) (Alciato et al., 1990; LeWitt et al., 2008). An example is rolofylline (KW-3902, **4**, Figure 1), which carries a bulky noradamantanyl residue at the 8-position and acts as a selective A_1_ AR receptor antagonist (LeWitt et al., 2008). Istradefylline (KW-6002, **5**, Figure 1), a potent, selective A_2A_ AR antagonist was approved for the treatment of Parkinson's disease (PD) in Japan (LeWitt et al., 2008; Dungo and Deeks, 2013; Kondo and Mizuno, 2015). It features a styryl residue at the xanthine 8-position and ethyl groups at the xanthine *N*1 and *N*3 nitrogen atoms. An *N*1-propargyl residue in combination with a *C*8-styryl substitution yielded the potent and selective A_2A_ AR antagonist MSX-2 (**6b**) and its prodrug MSX-3 (**6c**) prepared from the precursor MSX-1 (**6a**) (Sauer et al., 2000; Hockemeyer et al., 2004). PSB-601 (**7a**), PSB-0788 (**7b**) and PSB-603 (**7c**) are potent, selective A_2B_ AR antagonists. These xanthines carry a *para*-sulfonamido-substituted phenyl ring at the 8-position and are potential therapeutics for the treatment of asthma, pain and cancer (Feoktistov et al., 1998; Yan et al., 2006; Singh and Yadav, 2016; Hinz et al., 2018; Müller et al., 2018). The tricyclic purine derivatives PSB-10 (**8a**) and PSB-11 (**8b**) are selective A_3_ AR antagonists (Müller et al., 2002; Ozola et al., 2003).

Crystal structures of the AR subtypes A_1_ (Cheng et al., 2017; Glukhova et al., 2017) and A_2A_ (Doré et al., 2011; Liu et al., 2012; Sun et al., 2017) showed that large 8-substituents of xanthine derivatives point out of the receptor binding pocket toward the extracellular space. This makes *C*8 a privileged position for the attachment of fluorophores (Köse et al., 2018), solubilizing moieties (Daly et al., 1985), spin labels for electron paramagnetic resonance (EPR) studies (Ilaš et al., 2005) or linkers for dual-acting compounds (Jacobson, 2009). An example of a dual ligand is compound **9** (Jacobson, 2009).

Receptors other than ARs, and enzymes can also be addressed by selecting appropriate substituents at the xanthine scaffold. Stacofylline (**10**) inhibits the enzyme acetylcholinesterase; it contains a diethylaminocarbonylpiperazinyl residue connected via a propyl spacer to the 8-position of caffeine (Gallagher, 2004). Bamifylline (**11**), a phosphodiesterase inhibitor, carries a benzyl-substituent at *C*8 and is used as an analgesic, bronchodilatory and vasodilatory drug (Alciato et al., 1990). The phosphodiesterase inhibitor laprafylline (**12**) features, similar to stacofylline (**10**), a piperazinyl residue attached by an ethyl linker to the 8-position of 1-methyl-3-isobutylxanthine. Recently, dipeptidylpeptidase 4 (DPP-4) inhibitors have gained attention for the treatment of type 2 diabetes (Crepaldi et al., 2007; Costante et al., 2015). Xanthine-derived compounds, such as CN103373999A (**13**), bearing a piperazinylmethyl residue at the xanthine 8-position have been identified as potent DPP-4 inhibitors (Costante et al., 2015).

**Scheme 1:**
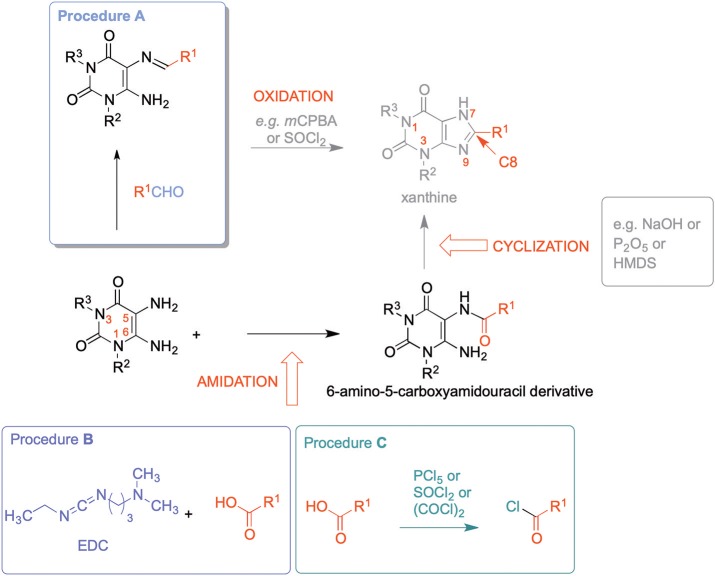
Syntheses of 8-substituted xanthine derivatives.

8-Substituted xanthines can be synthesized by reacting 5,6-diaminouracil derivatives with carboxylic acids or aldehydes (Scheme 1).

Different routes have been employed to obtain the required xanthine precursors. Condensation of 5,6-diaminouracils with aldehydes forming the corresponding imines [5-(arylidene- or alkylidene-amino)-6-aminouracils] as precursors, followed by oxidative cyclization is a commonly used route for the synthesis of 8-substituted xanthine derivatives (Hayallah et al., 2002; El-Sabbagh et al., 2007). However, aldehydes are less stable than the corresponding carboxylic acids, and commercial availability is often limited (Procedure A, Scheme 1) (Daly et al., 1985; Hayallah et al., 2002). Alternatively, 6-amino-5-carboxamidouracils can be prepared, which are the most frequently utilized xanthine precursors, that can be cyclized using a variety of methods, e.g., by sodium hydroxide or methylate, trimethylsilyl polyphosphate (PPSE), hexamethyldisilazane (HMDS) (Hayallah et al., 2002), or phosphorus pentoxide (Müller et al., 2008), depending on their reactivity and stability.

An established method for their preparation is the coupling of 5,6-diaminouracil derivatives with carboxylic acids in the presence of 1-ethyl-3-(3-dimethylaminopropyl)carbodiimide hydrochloride (EDC-HCl) as a coupling reagent (Procedure B, Scheme 1) (Sauer et al., 2000; Hayallah et al., 2002; Hockemeyer et al., 2004; Basu et al., 2017). Another method requires the activation of the carboxylic acid by formation of the carboxylic acid chloride (Procedure C, Scheme 1) (Jacobson et al., 1989; Hockemeyer et al., 2004). Procedure C had been used to establish a multigram-scale synthesis of istradefylline (**5**). Drawbacks of this reaction are long reaction times (16 h) for the formation of the amide, only moderate yields (65%), and importantly, an additional step due to conversion of the acid into the corresponding acid chloride using hazardous chlorinating reagents. Furthermore, carboxylic acid chlorides are less stable than the corresponding carboxylic acids rendering storage and handling more demanding (Hockemeyer et al., 2004). Coupling reactions with the irritant and moisture-sensitive EDC-HCl also suffer from rather long reaction times, and typically provide moderate yields requiring tedious purification (Sauer et al., 2000; Hockemeyer et al., 2004).

All of these disadvantages motivated us to search for an alternative amide coupling procedure for the preparation of 6-amino-5-carboxamidouracil derivatives being the most stable and easily storable xanthine precursors. Our aim was to develop a fast and effective coupling method applicable to a variety of diaminouracils and carboxylic acids that would allow simple work-up and straightforward isolation of the desired product (**Scheme 3**).

## Experimental

Chemicals were purchased from Merck (Darmstadt, Germany), ABCR (Karlsruhe, Germany) or TCI (Eschborn, Germany). Analytical thin layer chromatography (TLC) was performed on TLC plates F_254_ (Merck) and analyzed using UV light. High resolution mass spectra (HR-MS) were recorded on a micrOTOF-Q mass spectrometer (Bruker), low resolution mass spectra (LR-MS) on an API 2000 (Applied Biosystems) mass spectrometer. ^1^H NMR and ^13^C NMR spectra were recorded in CDCl_3_ or (CD_3_)_2_SO on a Bruker Ascend 600 MHz NMR-spectrometer operating at 600.18 MHz (^1^H), and 150.93 MHz (^13^C). Chemical shifts (δ) are reported in ppm and are referenced to the chemical shifts of the residual solvent proton(s) present in chloroform δ [(CHCl_3_) = 7.26 ppm for the ^1^H NMR spectra and δ (CDCl_3_) = 77.16 ppm for the ^13^C NMR spectra] and in dimethylsulfoxide δ ((CH_3_)_2_SO) = 2.50 ppm for the ^1^H NMR spectra and δ ((CD_3_)_2_SO) = 39.52 ppm for the ^13^C NMR spectra. Multiplicity: s, singlet; d, doublet; q, quartet; m, multiplet. Coupling constants (*J*) are shown in Hertz (Hz). The infrared spectra were recorded as solid samples on an ALPHA-T (Bruker) with a Platinum ATR Module using the Opus software. The IR spectra were measured in the attenuated total reflection (ATR) mode in the region of 4,000–385 cm^−1^ (s, strong; m, medium; w, weak) and are reported in cm^−1^.

### General Amide Formation Procedure

To a solution of the respective carboxylic acid (1.0 equiv.) and COMU (1.1 equiv.) dissolved in a minimum of dimethylformamide (DMF), a mixture of diaminouracil (1.1 equiv.) and *N*,*N*-diisopropylethylamine (DIPEA) (1.1 equiv.) dissolved in a minimum DMF was added dropwise. The reaction was stirred for 5–10 min at room temperature, and water was added. The resulting precipitate was filtered off, washed with water and dried under reduced pressure. Most of the reactions were performed using 300 mg of the respective diaminouracil and 4 ml of DMF. The product was precipitated using 20 ml of water and washed with small portions of water (10 ml). The reaction generally performed well from 60 mg up to 1.5 g of diaminouracil as a precursor. For the 1.5 g scale 8 ml of DMF were used for dissolution, and 40 ml of water for precipiation, and 20 ml for the subsequent washing step. All other conditions were identical, and virtually the same percentage of yield as obtained independent of the scale of the reaction.

### (9*H*-fluoren-9-yl)methyl 4-(2-((6-amino-3- methyl -2,4-dioxo-1,2,3,4-tetrahydro-pyrimi din-5-yl)amino)-2-oxoethyl)piperazine-1-carboxylate (21)

Yield: 62% (white solid); mp 181–184°C; ^1^H NMR (500 MHz, DMSO-*d*_6_) δ 10.48 (s, 1H, N1-H), 8.21 (s, 1H, CONH), 7.90 (d, *J* = 7.5 Hz, 2H, H_arom_), 7.63 (d, *J* = 7.5 Hz, 2H, H_arom_), 7.42 (t, *J* = 7.4 Hz, 2H. H_arom_), 7.35 (td, *J* = 7.4, 1.1 Hz, 2H, H_arom_), 6.01 (s, 2H, NH_2_), 4.40 (d, *J* = 6.5 Hz, 2H, CH_2_), 4.28 (t, *J* = 6.4 Hz, 1H, CH), 3.40–3.32 (m, 4H, 2 × CH_2_), 3.06 (s, 3H, CH_3_), 3.05–3.01 (m, 2H, CH_2_), 2.48–2.37 (m, 4H, 2 × CH_2_). ^13^C NMR (126 MHz, DMSO) δ 169.5 (CON), 160.7 (C6), 154.3 (OCON), 149.9 (CO), 149.7 (CO), 143.8 (2C, C_arom_), 140.8 (2C, C_arom_), 127.6 (2C, C_arom_), 127.1 (2C, C_arom_), 124.9 (2C, C_arom_), 120.1 (2C, C_arom_), 86.7 (C5), 66.4 (CH_2_), 61.0 (CH_2_), 52.2 (2C, 2 × CH_2_), 46.8 (2C, 2 × CH_2_), 43.3 (CH), 26.4 (CH_3_). IR (cm^−1^): ṽ = 3,556 (w), 33,481 (w), 3,327 (w), 3,208 (w), 3,010 (w), 2,949 (w), 2,895 (w), 2,811 (w), 2,757 (w), 1,730 (m), 1,688 (s), 1,655 (m), 1,556 (s), 1,505 (s), 1,458 (s), 1,442 (s), 1,289 (w), 1,237 (s), 1,203 (w), 1,124 (s), 1,082 (m), 1,006 (m), 966 (m), 755 (s), 737 (s), 641 (w),621 (w), 576 (m), 499 (s), 412 (s). HRMS (ESI-QTOF) calculated for C_26_H_28_N_6_O_5_ [M+H]^+^: 505.2194; found: 505.2190.

### *N*-(6-amino-1-methyl-2,4-dioxo-1,2,3,4-tetrahydropyrimidin-5-yl)benzamide (22)

Yield: 78% (white solid); mp > 320°C; ^1^H NMR (500 MHz, DMSO-*d*_6_) δ 10.59 (s, 1H, N3-H), 8.83 (s, 1H, CONH), 7.99 (d, *J* = 7.8 Hz, 2H, H_arom_), 7.54 (t, *J* = 6.8 Hz, 1H, H_arom_), 7.48 (q, *J* = 7.9, 7.3 Hz, 2H, H_arom_), 6.65 (s, 2H, NH_2_), 3.27 (s, 3H, CH_3_). ^13^C NMR (DMSO, 126 MHz) δ 166.6 (CON), 160.0 (C6), 153.7 (CO), 150.4 (CO), 134.7 (C_arom_), 131.2 (C_arom_), 128.1 (2C, C_arom_), 128.1(2C, C_arom_), 87.8 (C5), 29.2 (CH_3_). IR (cm^−1^): ṽ = 3,342 (w), 3,201 (w), 3,063 (w), 1,778 (w), 1,713 (s), 1,638 (s), 1,584 (s), 1,506 (s), 1,484 (s), 1,390 (w), 1,294 (m), 1,263 (w), 1,220 (w), 1,176 (w), 1,072 (w), 1,012 (w), 891 (w), 782 (m), 744 (w), 715 (s), 686 (w), 584 (m), 545 (s), 477 (s), 428 (w). HRMS (ESI-QTOF) calculated for C_12_H_12_N_4_O_3_ [M+H]^+^: 261.0982; found: 261.0981.

### *N*-(6-amino-2,4-dioxo-1,3-dipropyl-1,2,3,4-tetrahydropyrimidin-5-yl)-4-methoxy-benzamide (23)

Yield: 87% (off-white solid); mp 109–112°C; ^1^H NMR (600 MHz, DMSO-*d*_6_) δ 8.73 (s, 1H, CONH), 7.95 (d, *J* = 8.9 Hz, 2H, H_arom_), 7.00 (d, *J* = 8.9 Hz, 2H, H_arom_), 6.65 (s, 2H, NH_2_), 3.86–3.82 (m, 2H, N1-CH_2_ or N3-CH_2_), 3.82 (s, 3H, OCH_3_), 3.75–3.68 (m, 2H, N1-CH_2_ or N3-CH_2_), 1.57 (dt, *J* = 15.1, 7.5 Hz, 2H, CH_2_), 1.51 (dt, *J* = 14.8, 7.6 Hz, 2H, CH_2_), 0.89 (t, *J* = 7.4 Hz, 3H, CH_3_), 0.83 (t, *J* = 7.5 Hz, 3H, CH_3_). 13C NMR (DMSO, 151 MHz) δ 166.1 (CON), 161.6 (C_arom_), 159.2 (C6), 151.7 (CO), 150.4 (CO), 129.8 (C_arom_), 126.8 (C_arom_), 113.1 (C_arom_), 87.6 (C5), 55.3 (OCH_3_), 43.7 (N1-CH_2_ or N3-CH_2_), 41.8 (N1-CH_2_ or N3-CH_2_), 20.8 (2C, CH_2_), 11.2 (CH_3_), 10.7 (CH_3_). IR (cm^−1^): ṽ = 3,416 (w), 3,348 (w), 3,219 (w), 2,963 (w), 2,939 (w), 2,877 (w), 2,841 (w), 1,695 (m), 1,636 (m), 1,605 (s), 1,488 (s), 1,415 (m), 1,381 (w), 1,259 (s), 1,191 (m), 1,114 (w), 1,080 (w), 1,029 (m), 901 (w), 852 (m), 762 (m), 551 (s), 513 (s). HRMS (ESI-QTOF) calculated for C_18_H_24_N_4_O_4_ [M+H]^+^: 361.1870; found: 361.1885.

### 4-Nitrophenyl 4-((6-amino-3-ethyl-2,4- dioxo-1,2,3,4-tetrahydropyrimidin-5-yl)-carbamoyl) benzenesulfonate (24)

Product was purified by column chromatography (CH_2_Cl_2_/MeOH, 95:5). Yield: 78% (yellowish solid); mp 203–206°C; ^1^H NMR (500 MHz, DMSO-*d*_6_) δ 10.49 (s, 1H, N1-H), 9.21 (s, 1H, CONH), 8.30–8.26 (m, 2H, H_arom_), 8.21–8.17 (m, 2H, H_arom_), 8.06–8.02 (m, 2H, H_arom_), 7.42–7.36 (m, 2H, H_arom_), 6.22 (s, 2H, NH_2_), 3.75 (q, *J* = 7.0 Hz, 2H, N3-CH_2_), 1.06 (t, *J* = 7.0 Hz, 3H, CH_3_).^13^C NMR (DMSO, 126 MHz) δ 164.8 (CON), 160.3 (C6), 153.0 (C_arom_), 150.5 (CO), 149.7 (CO), 146.2 (C_arom_), 140.6 (C_arom_), 135.6 (C_arom_), 129.4 (2C, C_arom_), 128.2 (2C, C_arom_), 125.9 (2C, C_arom_), 123.3 (2C, C_arom_), 86.4 (C5), 34.4 (N3-CH_2_), 13.3 (CH_3_). IR (cm^−1^): ṽ = 3,304 (w), 3,185 (w), 3,078 (w), 2,971 (w), 2,917 (w), 2,851 (w), 1,734 (m), 1,627 (m), 1,507 (s), 1,480 (s), 1,374 (s), 1,349 (s), 1,314 (m), 1,293 (m), 1,203 (s), 1,153 (s), 1,091 (m), 1,012 (w), 866 (s), 757 (s), 733 (w), 692 (m), 630 (w), 606 (s), 564 (s), 500 (s), 445 (m). HRMS (ESI-QTOF) calculated for C_19_H_17_N_5_O_8_S[M+H]^+^: 476.0871; found: 476.0860.

### (2*R*,3*as*,5*S*,6*as*)-*N*-(6-amino-2,4-dioxo-1,3-dipropyl-1,2,3,4-tetrahydropyrimidin-5-yl)octahydro-2,5-methanopentalene-3*a*-carboxamide (25)

Most of the compound precipitated overnight. To increase the yield, the filtrate was extracted with diethyl ether, dried over MgSO_4_, and after filtration the solvent was removed *in vacuo*. Yield: 99% (slightly brown solid); mp 153–157°C; ^1^H NMR (600 MHz, DMSO-*d*_6_) δ 7.74 (s, 1H, CONH), 6.33 (s, 2H, NH_2_), 3.88–3.78 (m, 2H, NCH_2_), 3.74–3.62 (m, 2H, NCH_2_), 2.74–2.69 (m, 1H, CH), 2.24 (s, 2H, H_adamantyl_), 2.05 (d, J = 9.8 Hz, 2H, H_adamantyl_), 1.83–1.76 (m, 4H, H_adamantyl_), 1.55 (p, J = 7.2 Hz, 6H, H_adamantyl_ and CH_2_CH_3_), 1.49 (q, J = 7.4 Hz, 2H, CH_2_CH_3_), 0.88 (t, J = 7.4 Hz, 3H, CH_3_), 0.82 (t, J = 7.4 Hz, 3H, CH_3_). ^13^C NMR (DMSO, 151 MHz) δ 177.1 (CON), 158.9 (C6), 151.4 (CO), 150.3 (CO), 88.2 (C5), 54.7 (C_adamantyl_), 46.8 (NCH_2_), 43.6 (NCH_2_), 43.2 (C_adamantyl_), 42.3 (C_adamantyl_), 41.8 (C_adamantyl_), 37.0 (C_adamantyl_), 34.5 (C_adamantyl_), 20.8 (C_adamantyl_), 11.2 (C_adamantyl_), 10.7 (C_adamantyl_). IR (cm^−1^): ṽ = 3,425 (w), 3,331 (w), 2,925 (w), 2,871 (w), 1,694 (s), 1,627 (m), 1,556 (s), 1,492 (s), 1,374 (w), 1,338 (w), 1,272 (m), 1,226 (m), 1,204 (m), 1,111 (w), 1,085 (w), 899 (w), 843 (w), 763 (w), 716 (w), 549 (m), 475 (w), 429 (w). HRMS (ESI-QTOF) calculated for C_20_H_30_N_4_O_3_ [M+H]^+^: 375.2391; found: 375.2389.

### *N*-(6-amino-3-methyl-2,4-dioxo-1,2,3,4-tetrahydropyrimidin-5-yl)cyclopentane-carboxamide (26)

Yield: 70% (white solid); mp > 320°C; ^1^H NMR (500 MHz, DMSO-*d*_6_) δ 10.38 (s, 1H, N1-H), 8.21 (s, 1H, CONH), 5.77 (s, 2H, NH_2_), 3.04 (s, 3H, NCH_3_), 2.74 (p, *J* = 8.0 Hz, 1H, CH), 1.84–1.75 (m, 2H, H_cyclopentyl_), 1.74–1.66 (m, 2H, H_cyclopentyl_), 1.61 (qt, *J* = 10.3, 4.3 Hz, 2H, H_cyclopentyl_), 1.50 (dtt, *J* = 9.2, 5.6, 2.9 Hz, 2H, H_cyclopentyl_). ^13^C NMR (DMSO, 126 MHz) δ 176.0 (CON), 160.9 (C6), 150.1 (CO), 150.0 (CO), 87.7 (C5), 44.1 (C_cyclopentyl_), 30.1 (2C, C_cyclopentyl_), 26.6 (CH3), 25.9 (2C, C_cyclopentyl_). IR (cm^−1^): ṽ = 3,328 (w), 3,173 (w), 2,967 (w), 2,951 (w), 2,872 (w), 1,720 (s), 1,651 (s), 1,633 (s), 1,552 (s), 1,497 (s), 1,456 (s), 1,380 (w), 1,302 (w), 1,211 (m), 1,170 (w), 1,120 (w), 1,024 (w), 996 (w), 961 (w), 945 (w), 755 (s), 711 (m), 662 (m), 592 (s), 549 (m), 512 (s), 471 (m), 417 (s). HRMS (ESI-QTOF) calculated for C_11_H_16_N_4_O_3_ [M+H]^+^: 253.1295; found: 253.1294.

### (*E*)-*N*-(6-amino-1,3-diethyl-2,4-dioxo-1,2,3,4-tetrahydropyrimidin-5-yl)-3-(3,4-dimethoxyphenyl)acrylamide (27)

Yield: 70% (white solid); mp 108–112°C; ^1^H NMR (500 MHz, DMSO-*d*_6_) δ 8.49 (s, 1H, CONH), 7.39 (d, *J* = 15.8 Hz, 1H, CH), 7.18 (d, *J* = 1.9 Hz, 1H, H_arom_), 7.15 (dd, *J* = 8.3, 1.9 Hz, 1H, H_arom_), 7.01 (d, *J* = 8.4 Hz, 1H, H_arom_), 6.71 (d, *J* = 15.8 Hz, 1H, CH), 6.62 (s, 2H, NH_2_), 3.93 (q, *J* = 6.9 Hz, 2H, N1-CH_2_ or N3-CH_2_), 3.80 (d, *J* = 6.3 Hz, 8H, N1-CH_2_ or N3-CH_2_ and 2 × OCH_3_), 1.14 (t, *J* = 7.0 Hz, 3H, CH_3_), 1.07 (t, *J* = 7.0 Hz, 3H, CH_3_). ^13^C NMR (DMSO, 126 MHz) δ 165.6 (CON), 158.8 (C6), 151.0 (C_arom_), 150.1 (CO), 149.8 (CO), 148.9 (C_arom_), 138.8 (CH), 127.8 (C_arom_), 121.1 (C_arom_), 120.4 (C_arom_), 111.9 (C_arom_), 110.2 (CH), 87.8 (C5), 55.5 (OCH_3_), 55.4 (OCH_3_), 37.6 (N1-CH_2_), 35.4 (N3-CH_2_), 13.2 (2C, CH_3_). IR (cm^−1^): ṽ = 3,370 (w), 3,197 (w), 2,987 (w), 2,939 (w), 2,840 (w), 1,705 (s), 1,661 (m), 1,644 (m), 1,581 (s), 1,509 (s), 1,464 (s), 1,419 (m), 1,374 (w), 1,325 (w), 1,267 (s), 1,238 (s), 1,185 (s), 1,161 (s), 1,139 (s), 1,024 (m), 974 (m), 848 (w), 794 (m), 760 (m), 671 (m), 554 (s), 529 (s), 448 (s). HRMS (ESI-QTOF) calculated for C_19_H_24_N_4_O_5_ [M+H]^+^: 389.1819; found: 389.1812.

### (*E*)-*N*-(6-amino-2,4-dioxo-3-(prop-2-yn-1-yl)-1,2,3,4-tetrahydropyrimidin-5-yl)-3-(3-methoxyphenyl)acrylamide (28)

Yield: 83% (white solid); mp 295–298°C; ^1^H NMR (500 MHz, DMSO-*d*_6_) δ 10.59 (s, 1H, N1-H), 8.67 (s, 1H, CONH), 7.44 (d, *J* = 15.8 Hz, 1H, CH), 7.35 (t, *J* = 7.9 Hz, 1H, H_arom_), 7.17 (d, *J* = 7.7 Hz, 1H, H_arom_), 7.15–7.12 (m, 1H, H_arom_), 6.98–6.95 (m, 1H, H_arom_), 6.82 (d, *J* = 15.8 Hz, 1H, CH), 6.13 (s, 2H, NH_2_), 4.44 (d, *J* = 2.4 Hz, 2H, N3-CH_2_), 3.80 (s, 3H, OCH_3_), 3.03 (t, *J* = 2.4 Hz, 1H, H_propargyl_). ^13^C NMR (DMSO, 126 MHz) δ 165.0 (CON), 159.6 (C6), 150.3 (CO), 149.1 (CO), 138.8 (C_arom_ or CH), 136.4 (C_arom_ or CH), 130.0 (C_arom_), 122.7 (C_arom_), 119.7 (C_arom_), 115.2 (C_arom_), 112.7 (CH), 86.9 (C5), 79.9 (C_propargyl_), 72.4 (C_propargyl_), 55.1 (OCH_3_), 28.9 (N3-CH_2_). IR (cm^−1^): ṽ = 3,393 (w), 3,290 (w), 3,252 (w), 3,120 (w), 1,727 (s), 1,707 (m), 1,650 (s), 1,625 (m), 1,598 (s), 1,550 (s), 1,508 (s), 1,492 (s), 1,447 (s), 1,410 (w), 1,388 (w), 1,340 (m), 1,313 (m), 1,295 (m), 1,250 (s), 1,187 (m), 1,159 (m), 1,038 (w), 1,016 (w), 976 (s), 944 (w), 930 (w), 903 (w), 885 (w), 836 (w), 778 (m), 759 (s), 698 (s), 643 (s), 564 (s), 456 (s). HRMS (ESI-QTOF) calculated for C_17_H_16_N_4_O_4_ [M+H]^+^: 341.1244; found: 341.1241.

### *N*-(6-amino-1,3-dimethyl-2,4-dioxo-1,2,3,4-tetrahydropyrimidin-5-yl)-2-phenyl–acetamide (29)

Yield: 85% (white solid); mp 258–261°C; ^1^H NMR (500 MHz, DMSO-*d*_6_) δ 8.58 (s, 1H, CONH), 7.38–7.33 (m, 2H, H_arom_), 7.32–7.27 (m, 2H, H_arom_), 7.21 (tt, *J* = 6.4, 1.1 Hz, 1H, H_arom_), 6.54 (s, 2H, NH_2_), 3.59 (s, 2H, CH_2_), 3.31 (s, 3H, CH_3_), 3.11 (s, 3H, CH_3_). ^13^C NMR (DMSO, 126 MHz) δ 170.7 (CON), 159.3 (C6), 152.0 (CO), 150.5 (CO), 136.5 (C_arom_), 129.2 (C_arom_), 128.0 (C_arom_), 126.1 (C_arom_), 87.5 (C5), 42.0 (CH_2_), 30.0 (CH_3_), 27.5 (CH_3_). IR (cm^−1^): ṽ = 3,322 (w), 3,190 (w), 1,699 (m), 1,667 (s), 1,643 (m), 1,583 (s), 1,496 (s), 1,421 (w), 1,381 (w), 1,344 (w), 1,322 (w), 1,225 (m), 1,164 (w), 1,153 (m), 1,057 (w), 1,028 (w), 979 (w), 954 (w), 935 (w), 903 (w), 837 (w), 756 (m), 728 (s), 693 (m), 557 (s), 535 (m), 487 (s), 438 (m). HRMS (ESI-QTOF) calculated for C_14_H_16_N_4_O_3_ [M+H]^+^: 289.1295; found: 289.1296.

### *N*-(6-amino-2,4-dioxo-1,3-dipropyl-1,2,3,4-tetrahydropyrimidin-5-yl)benzamide (30)

Yield: 85% (off-white solid); mp. 121–124°C; ^1^H NMR (600 MHz, chloroform-*d*_1_) δ 8.19 (s, 1H, CONH), 7.93 (d, *J* = 7.4 Hz, 2H, H_arom_), 7.52 (t, *J* = 7.1 Hz, 1H, H_arom_), 7.43 (t, *J* = 7.5 Hz, 2H, H_arom_), 5.71 (s, 2H, NH_2_), 3.83 (dt, *J* = 14.3, 7.8 Hz, 4H, 2 × NCH_2_), 1.72 (q, *J* = 7.4 Hz, 2H, CH_2_), 1.61 (q, *J* = 7.4 Hz, 2H, CH_2_), 0.99 (t, *J* = 7.3 Hz, 3H, CH_3_), 0.91 (t, *J* = 7.4 Hz, 3H, CH_3_). ^13^C NMR (CDCl3, 151 MHz) δ 166.9 (CON), 160.2 (C6), 150.2 (CO), 148.0 (CO), 133.4 (C_arom_), 132.3 (C_arom_), 128.8 (C_arom_), 127.6 (C_arom_), 92.3 (C5), 44.9 (NCH_2_), 43.6 (NCH_2_), 21.6 (CH_2_), 21.3 (CH_2_), 11.4 (CH_3_), 11.3 (CH_3_). IR (cm^−1^): ṽ = 3,364 (w), 3,216 (w), 2,963 (w), 2,931 (w), 2,874 (w), 1,696 (m), 1,664 (m), 1,578 (s), 1,508 (s), 1,463 (s), 1,414 (m), 1,278 (m), 1,160 (w), 1,073 (w), 1,000 (w), 900 (w), 842 (w), 764 (m), 689 (m), 543 (s), 456 (m). HRMS (ESI-QTOF) calculated for C_17_H_22_N_4_O_3_ [M+H]^+^: 331.1765; found: 331.1767.

### *N*-(6-amino-3-ethyl-2,4-dioxo-1,2,3,4-tetrahydropyrimidin-5-yl)cinnamamide (31)

Yield: 80% (off-white solid); mp > 320°C; ^1^H NMR (500 MHz, DMSO-*d*_6_) δ 10.43 (s, 1H, N1-H), 8.68 (s, 1H, CONH), 7.58 (d, *J* = 7.4 Hz, 2H, H_arom_), 7.50–7.37 (m, 4H, H_arom_ + H_vinyl_), 6.83 (d, *J* = 15.9 Hz, 1H, H_vinyl_), 5.99 (s, 2H, NH_2_), 3.74 (q, *J* = 6.5 Hz, 2H, CH_2_), 1.06 (t, *J* = 6.7 Hz, 3H, CH_3_). ^13^C NMR (DMSO, 126 MHz) δ 164.9 (CON), 160.3 (C6), 149.7 (CO), 149.5 (CO), 138.8 (C_vinyl_ or C_arom_), 135.0 (C_vinyl_ or C_arom_), 129.4 (C_vinyl_ or C_arom_), 129.0 (2C, C_arom_), 127.4 (2C, C_arom_), 122.4 (C_vinyl_ or C_arom_), 87.4 (C5), 34.4 (N3-CH_2_), 13.2 (CH_3_). IR (cm^−1^): ṽ = 3,315 (w), 3,166 (w), 3,065 (w), 3,026 (w), 2,976 (w), 2,940 (w), 2,913 (w), 1,723 (s), 1,646 (s), 1,617 (s), 1,557 (s), 1,490 (s), 1,427 (m), 1,381 (w), 133 (m), 1,291 (w), 1,192 (m), 1,161 (w), 1,047 (w), 999 (m), 741 (s), 713 (m), 586 (s), 543 (s), 505 (s), 487 (s), 450 (w), 433 (w). HRMS (ESI-QTOF) calculated for C_15_H_16_N_4_O_3_ [M+H]^+^: 301.1295; found: 301.1294.

### *N*-(6-amino-3-ethyl-2,4-dioxo-1,2,3,4-tetrahydropyrimidin-5-yl)-3-phenylpropanamide (32)

Yield: 90% (white solid); mp > 320°C; ^1^H NMR (500 MHz, DMSO-*d*_6_) δ 10.38 (s, 1H, N1-H), 8.39 (s, 1H, CONH), 7.28 (t, *J* = 7.4 Hz, 2H, H_arom_), 7.24 (d, *J* = 6.9 Hz, 2H, H_arom_), 7.18 (t, *J* = 7.1 Hz, 1H, H_arom_), 5.82 (s, 2H, NH_2_), 3.73 (q, *J* = 6.9 Hz, 2H, N3-CH_2_), 2.91–2.80 (m, 2H, CH_2_), 2.53 (dd, *J* = 9.2, 7.0 Hz, 2H, CH_2_), 1.04 (t, *J* = 7.0 Hz, 3H, CH_3_). ^13^C NMR (DMSO, 126 MHz) δ 171.7 (CON), 160.4 (C6), 149.9 (CO), 149.6 (CO), 141.5 (C_arom_), 128.3 (2C, C_arom_), 128.1 (2C, C_arom_), 125.8 (C_arom_), 87.2 (C5), 36.8 (CH_2_), 34.3 (N3-CH_2_), 30.9 (CH_2_), 13.2 (CH_3_). IR (cm^−1^): ṽ = 3,341 (w), 3,290 (w), 3,180 (w), 3,066 (w), 3,029 (w), 2,913 (w), 1,725 (m), 1,637 (s), 1,552 (s), 1,486 (s), 1,382 (m), 1,333 (m), 1,301 (m), 1,192 (w), 1,157 (m), 1,124 (w), 1,044 (w), 970 (w), 921 (w), 799 (w), 78 (w), 760 (s), 730 (m), 695 (m), 662 (m), 571 (s), 501 (s), 481 (s). HRMS (ESI-QTOF) calculated for C_15_H_18_N_4_O_3_ [M+H]^+^: 303.1452; found: 303.1454.

### *N*-(6-amino-3-ethyl-2,4-dioxo-1,2,3,4-tetrahydropyrimidin-5-yl)-2-phenylcyclo–propanecarboxamide (33)

Yield: 89% (white solid); mp 302–305°C; ^1^H NMR (500 MHz, DMSO-*d*_6_) δ 10.35 (s, 1H, N1-H), 8.68 (s, 1H, CONH), 7.29 (t, *J* = 7.5 Hz, 2H, H_arom_), 7.19 (d, *J* = 7.7 Hz, 1H, H_arom_), 7.14 (d, *J* = 7.9 Hz, 2H, H_arom_), 5.90 (s, 2H, NH_2_), 3.72 (q, *J* = 6.9 Hz, 2H, N3-CH_2_), 2.28 (dt, *J* = 9.5, 6.0 Hz, 1H, CH), 2.09 (dt, *J* = 8.8, 4.7 Hz, 1H, CH), 1.37 (dt, *J* = 9.0, 4.5 Hz, 1H, CH), 1.26–1.20 (m, 1H, CH), 1.04(t, *J* = 6.9 Hz, 3H, CH_3_). ^13^C NMR (DMSO, 126 MHz) δ 171.5 (CON), 160.4 (C6), 149.9 (CO), 149.5 (CO), 141.2 (C_arom_), 128.2 (2C, C_arom_), 125.9 (3C, C_arom_), 87.4 (C5), 34.4 (N3-CH_2_), 25.6 (C_cyclopropyle_), 24.3 (C_cyclopropyle_), 16.1 (C_cyclopropyle_), 13.2 (CH_3_). IR (cm^−1^): ṽ = 3,355 (w), 3,312 (w), 3,186 (w), 3,082 (w), 3,032 (w), 3,011 (w), 2,978 (w), 2,941 (w), 1,726 (s), 1,650 (s), 1,628 (s), 1,555 (s), 1,497 (s), 1,454 (s), 1,427 (m), 1,382 (w), 1,334 (m), 1,300 (m), 1,199 (m), 1,160 (w), 1,080 (w), 1,026 (w), 957 (w), 760 (s), 693 (m), 662 (m), 592 (m), 543 (m), 518 (s), 499 (m). HRMS (ESI-QTOF) calculated for C_16_H_18_N_4_O_3_ [M+H]^+^: 315.1452; found: 315.1460.

### *N*-(6-amino-3-ethyl-2,4-dioxo-1,2,3,4-tetrahydropyrimidin-5-yl)-2-phenoxyacet-amide (34)

Yield: 88% (off-white solid); mp 289–293°C; ^1^H NMR (500 MHz, DMSO-*d*_6_) δ 10.45 (s, 1H, N1-H), 8.53 (s, 1H, CONH), 7.31 (td, *J* = 7.4, 2.0 Hz, 2H, H_arom_), 7.05–6.99 (m, 2H, H_arom_), 6.97 (t, *J* = 7.3 Hz, 1H, H_arom_), 6.07 (s, 2H, NH_2_), 4.57 (s, 2H, COCH_2_), 3.73 (q, *J* = 7.0 Hz, 2H, N3-CH_2_), 1.05 (t, *J* = 7.0 Hz, 3H, CH_3_). ^13^C NMR (DMSO, 126 MHz) δ 167.9 (CON), 160.3 (C6), 157.9 (C_arom_), 150.2 (CO), 149.6 (CO), 129.4 (2C, C_arom_), 121.0 (C_arom_), 114.7 (2C, C_arom_), 85.9 (C5), 66.9 (COCH_2_), 34.4 (N3-CH_2_), 13.2 (CH_3_). IR (cm^−1^): ṽ = 3,364 (w), 3,321 (w), 3,273 (w), 3,170 (w), 1,716 (m), 1,689 (m), 1,643 (m), 1,574 (s), 1,487 (s), 1,458 (m), 1,379 (w), 1,339 (w), 1,279 (w), 1,249 (w), 1,221 (s), 1,167 (w), 1,111 (w), 1,084 (w), 1,065 (w), 924 (w), 830 (w), 791 (w), 753 (s), 6,966 (w), 635 (m), 578 (w), 534 (s), 508 (m), 440 (w). HRMS (ESI-QTOF) calculated for C_14_H_16_N_4_O_4_ [M+H]^+^: 305.1244; found: 305.1253.

### *N*-(6-amino-3-ethyl-2,4-dioxo-1,2,3,4-tetrahydropyrimidin-5-yl)-2-methyl-3-phenylpropanamide (35)

Yield: quantitative (white solid); mp 265–267°C; ^1^H NMR (500 MHz, DMSO-*d*_6_) δ 10.37 (s, 1H, N1-H), 8.42 (s, 1H, CONH), 7.28 (t, *J* = 7.4 Hz, 2H, H_arom_), 7.25–7.21 (m, 2H, H_arom_), 7.18 (t, *J* = 7.2 Hz, 1H, H_arom_), 5.51 (s, 2H, N3–NH_2_), 3.72 (q, *J* = 6.9 Hz, 2H, CH_2_), 2.98 (dd, *J* = 13.4, 6.2 Hz, 1H, CH), 2.78–2.68 (m, 1H, CH), 2.57–2.51 (m, 1H, CH), 1.04 (t, *J* = 7.0 Hz, 3H, CH_3_), 1.00 (d, *J* = 6.8 Hz, 3H, CH_3_). ^13^C NMR (DMSO, 126 MHz) δ 175.4 (CON), 160.3 (C6), 149.6 (CO), 149.5 (CO), 140.1 (C_arom_), 128.9 (2C, C_arom_), 128.1 (2C, C_arom_), 125.9 (C_arom_), 87.5 (C5), 41.0 (CCH_3_), 34.4 (N3-CH_2_), 16.8 (CH_3_), 13.2 (CH_3_). IR (cm^−1^): ṽ = 3,354 (w), 3,318 (w), 3,178 (w), 3,082 (w), 3,022 (w), 3,002 (w), 2,975 (w), 2,938 (w), 2,875 (w), 1,723 (s), 1,632 (s), 1,552 (s), 1,492 (s), 1,457 (s), 1,426 (s), 1,378 (m), 1,331 (w), 1,299 (m), 1,226 (w), 1,181 (w), 1,160 (w), 1,116 (w), 1,044 (w), 948 (w), 759 (s), 745 (m), 698 (s), 659 (m), 543 (s), 505 (s). HRMS (ESI-QTOF) calculated for C_16_H_20_N_4_O_3_ [M+H]^+^: 317.1608; found: 317.1617.

### *N*-(6-amino-3-ethyl-2,4-dioxo-1,2,3,4-tetrahydropyrimidin-5-yl)benzamide (36)

Yield: 87% (off-white solid); mp > 320°C; ^1^H NMR (500 MHz, DMSO-*d*_6_) δ 10.38 (s, 1H, N1-H), 8.86 (s, 1H, CONH), 7.99–7.91 (m, 2H, H_arom_), 7.56–7.51 (m, 1H, H_arom_), 7.47 (t, *J* = 7.5 Hz, 2H, H_arom_), 6.06 (s, 2H, NH_2_), 3.75 (q, *J* = 7.0 Hz, 2H, N3-CH_2_), 1.06 (t, *J* = 7.0 Hz, 3H, CH_3_). ^13^C NMR (126 MHz, DMSO) δ 166.4 (CON), 160.5 (C6), 150.4 (CO), 149.7 (CO), 134.5 (C_arom_), 131.1 (C_arom_), 128.0 (C_arom_), 127.8 (C_arom_), 87.1 (C5), 34.4 (N3-CH_2_), 13.3 (CH_3_). IR (cm^−1^): ṽ = 3,302 (w), 3,166 (w), 3,061 (w), 2,976 (w), 1,718 (m), 1,627 (m), 1,552 (s), 1,504 (s), 1,481 (s), 1,456 (s), 1,426 (s), 1,381 (m), 1,334 (w), 1,299 (m), 1,165 (w), 1,047 (w), 926 (w), 883 (w), 797 (m), 760 (m), 692 (m), 657 (m), 544 (s), 503 (m), 473 (m), 445 (w). HRMS (ESI-QTOF) calculated for C_13_H_14_N_4_O_3_ [M+H]^+^: 275.1139; found: 275.1142.

### *N*-(6-amino-3-ethyl-2,4-dioxo-1,2,3,4-tetrahydropyrimidin-5-yl)-2-phenylacetamide (37)

Yield: 80% (white solid); mp > 320°C; ^1^H NMR (500 MHz, DMSO-*d*_6_) δ 10.39 (s, 1H, N1-H), 8.58 (s, 1H, CONH), 7.35–7.31 (m, 2H, H_arom_), 7.28 (m, 2H, H_arom_), 7.23–7.19 (m, 1H, H_arom_), 5.90 (s, 2H, NH_2_), 3.71 (q, *J* = 7.0 Hz, 2H, N3-CH_2_), 3.56 (s, 2H, CH_2_), 1.03 (t, *J* = 7.0 Hz, 3H, CH_3_). ^13^C NMR (DMSO, 126 MHz) δ 170.6 (CON), 160.5 (C6), 150.1 (CO), 149.7 (CO), 136.6 (C_arom_), 129.4 (C_arom_), 128.2 (C_arom_), 126.3 (C_arom_), 87.5 (C5), 42.1 (COCH_2_), 34.5 (N3-CH_2_), 13.4 (CH_3_). IR (cm^−1^): ṽ = 3,349 (w), 3,297 (w), 3,184 (w), 3,065 (w), 2,980 (w), 2,909 (w), 2,885 (w), 1,729 (m), 1,638 (s), 1,547 (s), 1,483 (s), 1,421 (s), 1,331 (m), 1,294 (m), 1,216 (w), 1,180 (m), 1,155 (m), 1,031 (w), 963 (w), 926 (w), 793 (w), 758 (s), 694 (s), 661 (m), 599 (s), 488 (s). HRMS (ESI-QTOF) calculated for C_14_H_16_N_4_O_3_ [M+H]^+^: 289.1295; found: 289.1304.

### *N*-(6-amino-3-ethyl-2,4-dioxo-1,2,3,4-tetrahydropyrimidin-5-yl)-6-methylheptanamide (38)

Yield: 81% (white solid); mp 278–281°C; ^1^H NMR (500 MHz, DMSO-*d*_6_) δ 10.34 (s, 1H, N1-H), 8.24 (s, 1H, CONH), 5.82 (s, 2H, NH_2_), 3.70 (q, *J* = 7.0 Hz, 2H, N3-CH_2_), 2.24–2.12 (m, 2H, COCH_2_), 1.55–1.45 (m, 3H, CH_2_ and CH), 1.34–1.23 (m, 2H, CH_2_), 1.19–1.11 (m, 2H, CH_2_), 1.03 (t, *J* = 7.0 Hz, 3H, CH_3_), 0.85 (d, *J* = 6.6 Hz, 6H, 2 × CH_3_). ^13^C NMR (DMSO, 126 MHz) δ 172.7 (CON), 160.6 (C6), 150.0 (CO), 149.7 (CO), 87.6 (C5), 38.5 (CH_2_), 35.4 (CH_2_), 34.5 (N3-CH_2_), 27.4 (CH_2_), 26.7 (CH), 25.4 (CH_2_), 22.7 (2 × CH_3_), 13.4 (CH_3_). IR (cm^−1^): ṽ = 3,341 (w), 3,302 (w), 3,186 (w), 3,075 (w), 2,957 (w), 2,915 (w), 2,875 (w), 2,851 (w), 1,728 (m), 1,637 (s), 1,551 (s), 1,488 (s), 1,424 (s), 1,379 (m), 1,333 (m), 1,294 (m), 1,200 (w), 1,159 (m), 1,111 (w), 1,048 (w), 967 (w), 925 (w), 760 (s), 729 (w), 664 (m), 580 (s), 500 (s), 444 (m). HRMS (ESI-QTOF) calculated for C_14_H_24_N_4_O_3_ [M+H]^+^: 297.1921; found: 297.1924.

### (3*aS*,4*S*,5*S*,7*aR*)-*N*-(6-amino-3-ethyl-2,4-dioxo-1,2,3,4-tetrahydropyrimidin-5-yl) –octahydro-1*H*-2,5-methanoindene-4-carboxamide (39)

Filtrate was extracted with Et_2_O, dried over MgSO_4_ and the solvent removed in vacuo. Yield: 75% (off-white solid); mp > 320°C; ^1^H NMR (500 MHz, DMSO-*d*_6_) δ 10.33 (s, 1H, N1-H), 7.77 (s, 1H, CONH), 5.64 (s, 2H, NH_2_), 3.71 (q, J = 7.0 Hz, 2H, N3-CH_2_), 1.97 (s, 3H, H_noradamantane_), 1.87 (d, J = 2.7 Hz, 6H, H_noradamantane_), 1.69–1.63 (m, 6H, H_noradamantane_), 1.04 (t, J = 7.0 Hz, 3H, CH_3_). ^13^C NMR (DMSO, 126 MHz) δ 177.3 (CON), 160.2 (C6), 149.7 (CO), 149.6 (CO), 87.6 (C5), 38.6 (CH_2noradamantane_), 36.2 (CH_2noradamantane_), 34.3 (N3–CH_2_), 27.7 (4C, CH_noradamantane_), 13.2 (CH_3_). IR (cm^−1^): ṽ = 3,478 (w), 3,428 (w), 3,289 (w), 3,165 (w), 3,067 (w), 2,984 (w), 2,909 (w), 2,853 (w), 1,718 (m), 1,622 (s), 1,545 (s), 1,507 (s), 1,486 (s), 1,446 (s), 1,372 (w), 1,330 (w), 1,291 (m), 1,244 (w), 1,184 (w), 1,161 (w), 1,110 (w), 1,042 (w), 989 (w), 927 (w), 760 (s), 701 (w), 653 (m), 542 (s), 499 (m). HRMS (ESI-QTOF) calculated for C_17_H_24_N_4_O_3_ [M+H]^+^: 333.1921; found: 333.1922.

## Results and Discussion

Disadvantages of irritant and hazardous coupling procedures, long reaction times and moderate yields encouraged us to search for a new method to yield the desired 6-amino-5-carboxamidouracil derivatives. After initial experiments with various procedures, the coupling reagent COMU showed the most promising results. COMU, which was developed in 2009, does not contain a potentially explosive benzotriazole moiety, and is therefore safer than classical coupling reagents such as, for example, 1-[bis(dimethylamino)methylene]-1*H*-1,2,3-triazolo[4,5-*b*]pyridinium-3-oxide hexafluorophosphate (HATU). COMU shows high solubility, is stable in typically used solvents, can be easily removed due to the water-solubility of its products, and may be used for a broad range of carboxylic acids and amines yielding the corresponding amides ((El-Faham et al., 2009; El-Faham and Albericio, 2010, 2011); Hjørringgaard et al., 2012).

**Scheme 2:**
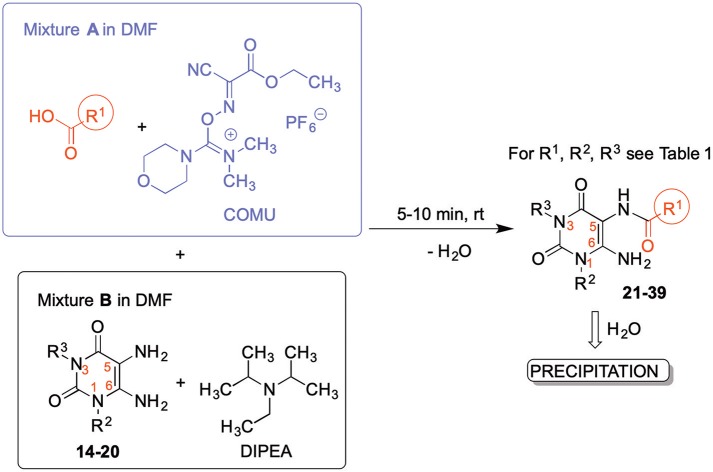
Formation of 6-amino-5-carboxamidouracils using COMU as a coupling reagent.

**Figure 2:**
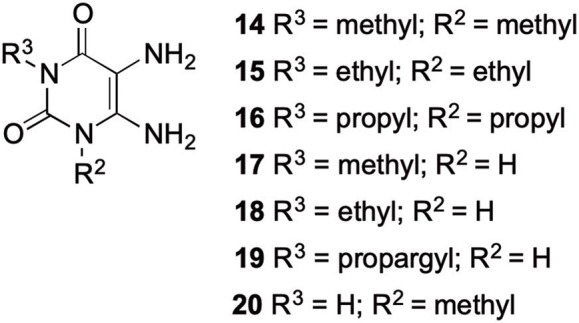
*N*1-Mono- and *N*1,*N*3-disubstituted 5,6-diaminouracil derivatives synthesized as starting materials for amide formation (for details see Supporting Material Data Sheet 1).

The synthetic procedure which led to differently substituted 6-amino-5-carboxamidouracils is shown in Scheme 2. Diaminouracil derivatives and carboxylic acids were used as starting materials and subjected to amide coupling using COMU. *N*1-mono- and *N*1,*N*3-disubstituted 5,6-diaminouracil derivatives (**14–20**, Figure 2) were individually prepared (for details see Supporting Material Data Sheet 1) according to previously described procedures and (Maxwell and Salivar, 1952; Müller et al., 1993; Hockemeyer et al., 2004), while the employed carboxylic acid derivatives were in most cases commercially available.

### Amide Coupling Reaction

Amide formation with the coupling reagent required the adjustment of different parameters, including solvent, reaction time, temperature and base. With DMF, DIPEA and COMU the optimal conditions were found (Scheme 2). The reaction may also be performed in other solvents, such as CH_2_Cl_2_, ethyl acetate or tetrahydrofurane (MacMillan et al., 2013), however, DMF is preferred resulting in short reaction times, and, importantly, the product can easily be precipitated in high purity by the addition of water. This renders a tedious isolation and purification procedure dispensable.

**Scheme 3:**
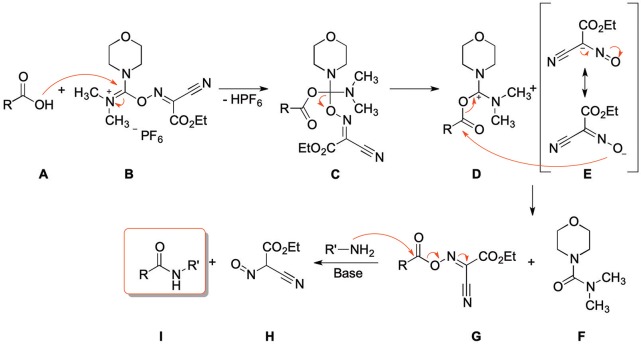
Proposed reaction mechanism of amide coupling with COMU.

Scheme 3 depicts the proposed reaction mechanism, which is based on the mechanism proposed for the synthesis of esters using COMU (Twibanire and Grindley, 2011). The first step is the nucleophilic attack of the carboxylic acid (**A**) at the uronium moiety of COMU (**B**) resulting in intermediate **C**. Decomposition of **C**, followed by addition of the resulting anion **E** to the carbonyl group of **D** and subsequent elimination of the urea derivative **F** leads to the activated carboxylic acid **G**. Finally, the corresponding amide derivative is formed by nucleophilic attack of an amine and elimination of the water-soluble side product **H**.

According to the proposed reaction mechanism, the carboxylic acid was converted to its active ester after dissolving it (1.0 equiv) together with COMU (1.1 equiv) in a minimum of DMF (mixture A, Scheme 2). Then, a solution of the 5,6-diaminouracil derivative (1.2 equiv) and diisopropylethylamine (DIPEA, 1.1 equiv) as a base dissolved in a minimum of DMF (mixture B) was added, followed by 5–10 min of stirring at room temperature (Scheme 2). Upon addition of cold water, the product precipitated. It was filtered off, washed with cold water, and dried under reduced pressure yielding the target compounds **21–39** (Table 1) in high purity and with yields ranging from 62 to 99%. Due to our interest in AR antagonists, we prepared various precursors for 8-substituted xanthines, which we could obtain in high yields and isolate by simple precipitation as shown for various examples (**22–29**). The 1,3-dipropyl derivatives **23** and **30** were formed in 87 and 85% yield, with 98 and 99% purity, respectively. Compound **23** is a precursor of the dual-acting A_1_ AR-opioid receptor ligands, such as **9**. Compound **22** was obtained in 78% yield and provides access to the A_3_ AR antagonists PSB-11 (**8b**). Compound **24**, the key compound for the synthesis of highly potent and selective A_2B_ AR antagonists, was successfully condensed and precipitated. The carboxylic acid for the synthesis of **24** was not commercially available and was therefore prepared according to a literature procedure (Borrmann et al., 2009). To gain a purity of over 95% for **24**, an additional chromatographic purification procedure was required. Compound **25**, the precursor of the A_1_ AR antagonist rolofylline (**4**), which contains an 8-noradamantanyl substituent, and propyl residues on *N*1 and *N*3, precipitated in high purity (99%); fractional precipitation after cooling to 0°C was required to give a final yield of 79%. The less bulky and less hydrophobic cyclopentanecarboxylic acid was reacted with 5,6-diamino-3-methyluracil to obtain amide **26** as a precursor for 8-cyclopentyltheophylline (**CPX**), and was isolated in 69% yield with 99% purity. The additional substituent on *N*1 can be easily introduced subsequently by alkylation according to literature procedures (Hockemeyer et al., 2004). The precursor **29** of the A_1_ AR antagonist bamifylline (**11**), with methyl groups at both uracil nitrogen atoms, precipitated immediately in 85% yield and 99% purity. Compound **27**, the precursor of the A_2A_ AR antagonist and anti-Parkinson drug istradefylline (**5**), precipitated in 70% yield with 97% purity. Amide formation with 3-methoxycinnamic acid, carrying the styrene moiety, which is required for the preparation of the potent and selective A_2A_ AR antagonists of the MSX series (**6a-c**), gave the 6-amino-5-carboxamidouracil precursor **28** in 83% isolated yield after precipiation.

**Table 1:** Formation of 6-amino-5-carboxamidouracil derivatives.

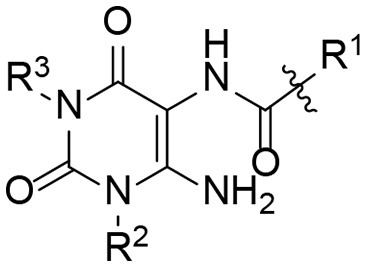						
**Precursors for xanthines with known bioactivity**						
**Compounds**	**R**^**1**^	**Precursor for (target)**	**R**^**2**^	**R**^**3**^	**Isolated yield (%)**	**Purity after precipitation (%)**
**21**	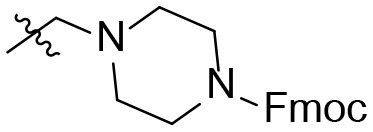	13 (DPP-4 inhibitor)	H	Me	62	99
**22**	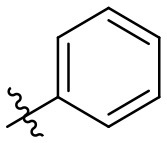	8b (A_3_ antagonist)	Me	H	78	96
**23**	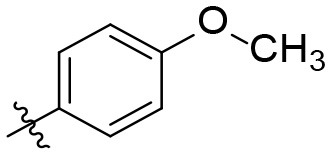	9 (A_1_ antagonists)	Pr	Pr	87	98
**24**^b^	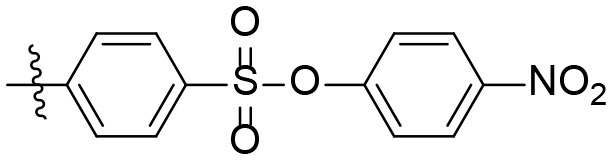	7a, 7b, 7c (A_2B_ antagonists)	H	Et	78	99^a^
**25**^c^	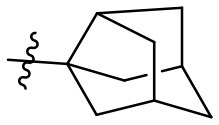	4 (A_1_ antagonist)	Pr	Pr	79	99
**26**	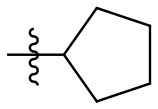	CPX (A_1_ antagonist)	H	Me	69	99
**27**^d^	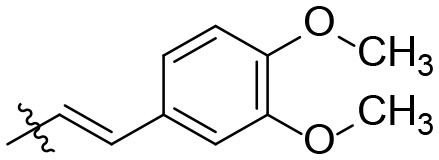	5 (A_2A_ antagonist)	Et	Et	70	97
**28**^e^	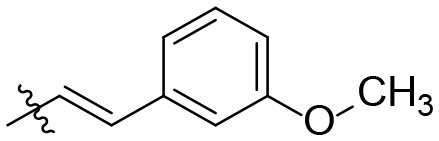	6a–c (A_2A_ antagonists)	H	Propargyl	83	98
**29**	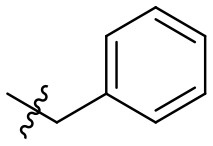	11 (A_1_ antagonists)	Me	Me	84	99
**Precursors for xanthines with various 8-substituents**						
**30**^f^	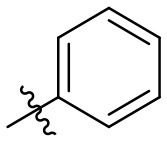	–	Pr	Pr	85	99
**31**	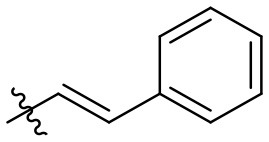	–	H	Et	80	94
**32**	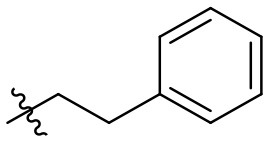	–	H	Et	90	99
**33**	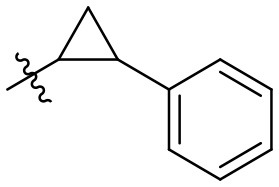	–	H	Et	89	98
**34**	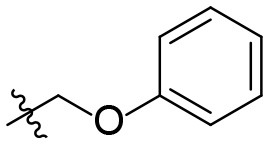	–	H	Et	88	99
**35**	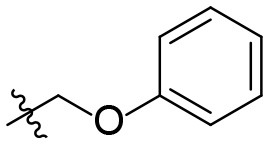	–	H	Et	99	99
**36**^g^	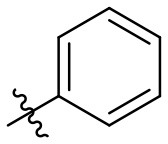	–	H	Et	87	99
**37**	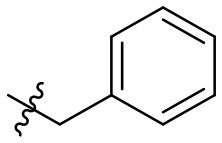	–	H	Et	80	90
**38**	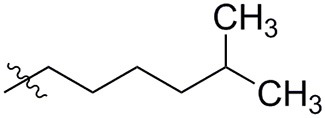	–	H	Et	81	99
**39**	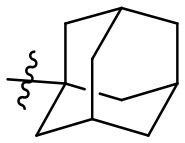	–	H	Et	78	87

a *Purity after additional column chromatography*.

b (Borrmann et al., 2009);

c (Moore et al., 1999);

d (Rabasseda et al., 2001);

e (Hockemeyer et al., 2004);

f (Daly et al., 1985);

g *(Rodríguez-Borges et al., 2010)*.

To investigate the impact of different carboxylic acid derivatives regarding precipitation of the product, we used 3-ethyldiaminouracil and various carboxylic acids as a test system for the formation of differently substituted 6-amino-5-carboxamidouracils (Table 1). Compound **32**, with a phenylpropionyl residue, was isolated in 90% yield. The analogous compound **33** containing a rigidified cyclopropyl ring gave a similar yield of 89%, as did the ether analog **34**. The presence of an α-methyl group in compound **35** resulted in quantitative product formation and precipitation. The 6-amino-5-carboxamidouracil **38** bearing an alkyl residue was isolated in 81% yield with 99% purity.

Comparing all reactions, we observed the following trends: 1,3-disubstituted uracils could be formed best in case of a bulky, hydrophobic carboxylic acid derivative, which favors precipitation from the DMF/H_2_O solution. Reactions of *N*1-unsubstituted diaminouracils generally gave higher product yields, and the products were easily precipitated. The melting points of those products were high indicating the formation of intermolecular hydrogen bonds in the solid state, which was confirmed by the crystal structure of **32** (see below).

### Structural Studies and Regioselectivity

Since 5,6-diaminouracil carries two amino groups, the question arises, which one forms the amide bond (Yang et al., 2015). Due to literature reports, the 5-amino group is proposed to react (Sauer et al., 2000; Hayallah et al., 2002; Hockemeyer et al., 2004). We checked this assumption by NMR and small single molecule X-ray crystallography, comparing the NMR signals of 6-aminouracil, 6-amino-5-nitrosouracil, 5,6-diaminouracil, and 5-amino-6-carboxamidouracil. We additionally applied 2-dimensional NMR spectroscopy, namely heteronuclear multiple bond correlation (HMBC) and nuclear Overhauser enhancement spectroscopy (NOESY), for determining the structure of amide **25**.

**Scheme 4:**
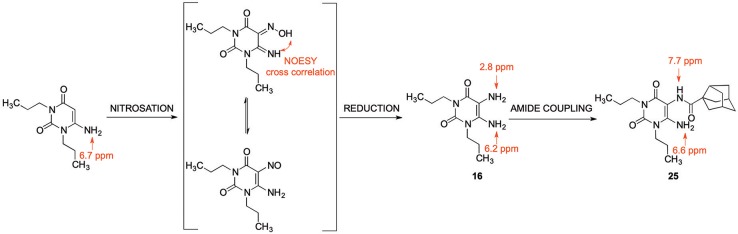
NMR signals of 6-aminouracil derivatives with various substituents in the 5-position, and NOESY cross correlation for structure/tautomer analysis determined in chloroform-*d*_1_.

In literature, the product of the first reaction step has been described as a 5-nitroso derivative. Based on our NMR experiments, the 5-(hydroxyimino)-6-imino derivative is the tautomer that is present in chloroform employed as a solvent (Scheme 4). The chemical shift of the 5-amino group in compound **16** indicates a magnetic shielding of the hydrogen atoms giving the nitrogen atom a more nucleophilic character, which is in accordance with our regioselectivity studies.

**Figure 3:**
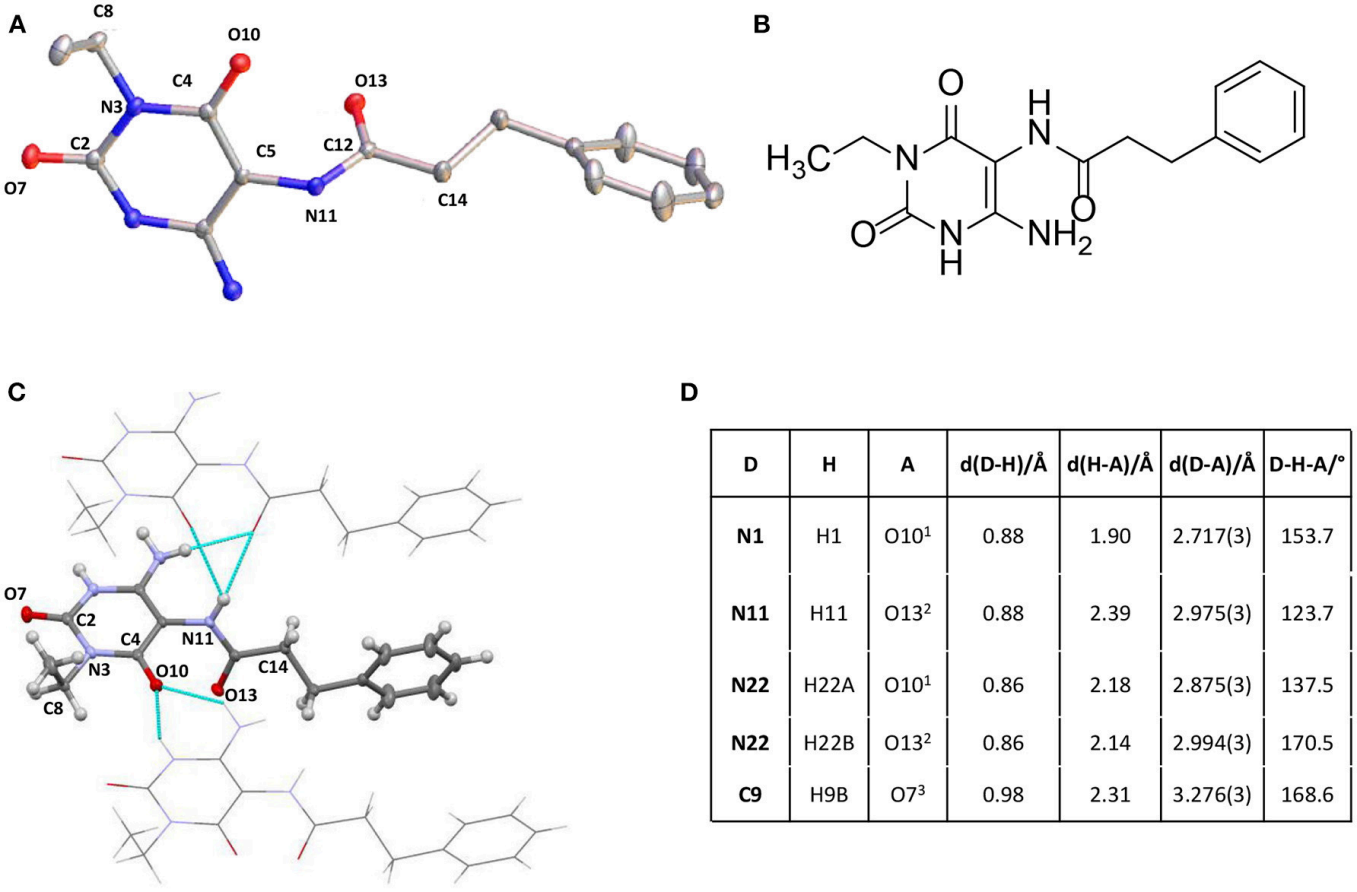
**(A)** Crystal structure of 6-amino-5-carboxamidouracil 32. **(B)** Structure of 32. **(C)** Intermolecular interactions building the crystal structure of 32. **(D)** Most important intermolecular hydrogen bonds of 32. Supporting Material Data Sheet 2.

Finally, we tried to obtain a crystal structure of **25**. Different crystallization experiments were performed but the crystallization of **25** has not been successful. Fortunately, compound **32**, crystallized from DMSO solution at room temperature, yielding a crystal of the size 0.4 × 0.2 × 0.08 mm. Measurement and analysis of the resulting crystal structure using a Bruker X8-KappaApexII instrument showed a monocline crystal system within the space group P2_1_. In accordance with the NMR experiment of **25** the crystal structure of **32** confirmed a regioselective amide coupling of the carboxylic acid with the 5,6-diaminouracil derivative in position 5. The crystal is mainly formed by intermolecular hydrogen bonds. π-Stacking or interaction with the solvent could not be observed. The most important intermolecular hydrogen bonds are summarized in Figure 3. All NH groups showed a donor functionalization and all oxygen atoms showed acceptor properties to surrounding molecules. Figure 3 visualizes these intermolecular interactions. The surrounding molecules are shaded while the intermolecular interactions are shown in turquoise. All bond lengths were in the expected range.

## Conclusions

In summary, we report on a new regioselective amide formation of 5,6-diaminouracil derivatives with carboxylic acids using the coupling reagent COMU which leads to the preparation of important precursors for xanthine derivatives. The reaction is completed after only 5–10 min of stirring at room temperature in DMF, followed by straightforward isolation of the formed amides by precipitation through the addition of water. After filtration, the 6-amino-5-carboxamidouracils were obtained in high isolated yields and showed in most cases purities of 90% or higher requiring no further chromatographic purification. The new procedure is advantageous with regard to reaction time and yields, and it avoids hazardous coupling or chlorinating reagents. In addition to several new derivatives, we synthesized the 6-amino-5-carboxamidouracil precursors of important, biologically active and literature-known xanthines utilizing the new method. The regioselectivity of the amide formation with the 5- rather than the 6-amino group of the uracil derivatives was proven by 2D-NMR spectroscopy and X-ray crystallography. The new regioselective amide coupling procedure allows the preparation of a variety of xanthine precursors. Moreover, the procedure will be well-suitable for automated and parallel synthesis.

## Author Contributions

DM performed most of the experiments. CM supervised the experiments. LW and MS had the idea to use COMU for the synthesis of xanthine precursors, and performed initial experiments elaborating reaction and workup conditions. GS determined the X-ray crystal structure. MS supervised experiments performed by LW. All authors contributed to writing the manuscript.

### Conflict of Interest Statement

The authors declare that the research was conducted in the absence of any commercial or financial relationships that could be construed as a potential conflict of interest.

## Supplementary Material

For details of synthetic procedures, crystallographic parameters, and NMR and IR spectra see Supporting Material Data Sheets 1 and 2.
