# Supplementary material for: Fast, Efficient, and Versatile Synthesis of 6-amino-5-carboxamidouracils as Precursors for 8-Substituted Xanthines
Source: Front Chem. 2019 Feb 18;7:56. doi: 10.3389/fchem.2019.00056 (PMC6387921; doi:10.3389/fchem.2019.00056)
Supplement: Supplementary file 1 [file Data_Sheet_1.PDF]

Supplementary Material

Fast, efficient and versatile synthesis of 6-amino-5-carboxamidouracils as precursors for 8-substituted xanthines

Daniel Marx‡, Lukas M. Wingen‡, Gregor Schnakenburg, Christa E. Müller*, Matthias S. Scholz

*** Correspondence:** Christa E. Müller: christa.mueller@uni-bonn.de

‡ These authors contributed equally to this work

TABLE OF CONTENTS

Synthesis of diaminouracil derivatives........................2

Crystallographic data........................3

^{1}H- and ^{13}C-NMR spectra of compound 21-39.. 8

IR spectra of compound **21-39** 28

References 37

Synthesis of diaminouracil derivatives

Different *N*1- and *N*3-substituted uracil derivatives required as precursors were synthesized according to literature procedures (Maxwell, et al., 1952; Müller et al., 1993; Hockemeyer et al., 2004). Due to the therapeutic potential of A_{2A} and A_{2B} adenosine receptor antagonists, we synthesized various 6-amino-5-carboxamidouracil precursors for xanthine derivatives containing ethyl and propargyl substituents at the uracil *N*1 atom, which are known to be beneficial for interaction with those adenosine receptor subtypes.

Scheme S1. Preperation of symmetrical *N*1,*N*3-dialkyl-5,6-diaminouracil derivatives.

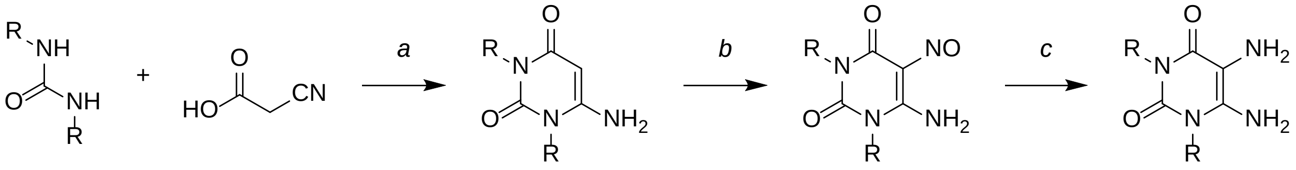

14 R = methyl

15 R = ethyl

16 R = propyl

14-16

Reagents and conditions: (a) Ac_2O, 60 °C, 3 h; (b) aq. AcOH, HNO_2, 50-60 °C; (c) sodium dithionite, NH_3/H_2O, 60 °C.

Scheme S2. Preperation of *N*1- or *N*3-substituted 5,6-diaminouracil derivatives.

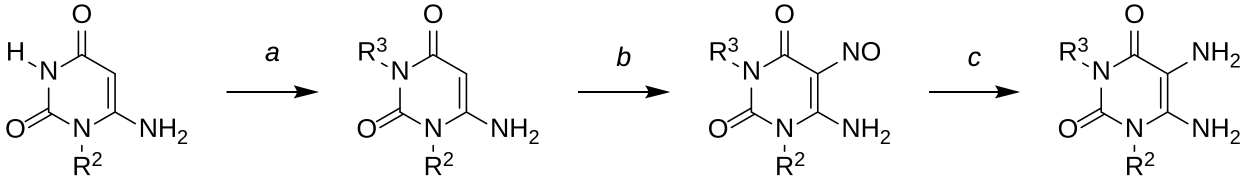

17 R^2 = H; R_3 = methyl

18 R^2 = H; R_3 = ethyl

19 R^2 = H; R_3 = propargyl

20 R^2 = methyl; R_3 = H (starting from the commercially available 1-methyl-6-aminouracil)

17-20

Reagents and conditions: (a) 2.1 equiv. of hexamethyldisilazane (HMDS), reflux, 60–70 °C, 1.7 equiv of methyl iodide for (**17**), ethyl iodide for (**18**) or 3-bromopropyne for (**19**); (b) aq. AcOH, HNO_2, 50-60 °C; (c) sodium dithionite, NH_3/H_2O, 60 °C.

Analytical data were in accordance with published data. For details see (Maxwell, et al., 1952; Müller et al., 1993; Hockemeyer et al., 2004).

Crystallographic data

CCDC 1878798 contains the supplementary crystallographic data for this paper. The data can be obtained free of charge from The Cambridge Crystallographic Data Centre via www.ccdc.cam.ac.uk/structures.

Table S1. Crystal data and structure refinement for compound 32.

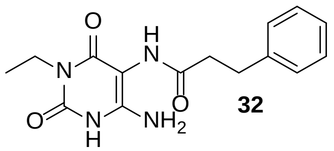

32

Identification code	GPHARM62, **32** // GXray5353f
Crystal Habitus	clear colourless plate
Device Type	Bruker X8-KappaApexII
Empirical formula	$C_{15}H_{18}N_4O_3$
Moiety formula	C15 H18 N4 O3
Formula weight	302.33
Temperature/K	100
Crystal system	monoclinic
Space group	$P2_1$
a/Å	4.6833(3)
b/Å	28.2023(18)
c/Å	5.4732(4)
α/°	90
β/°	98.690(3)

γ/°	90
Volume/Å^3	714.60(8)
Z	2
ρ_{calc}g/cm^3	1.405
μ/mm^{-1}	0.101
F(000)	320.0
Crystal size/mm^3	0.4 × 0.2 × 0.08
Absorption correction	multi-scan
Tmin; Tmax	0.6046; 0.7460
Radiation	MoKα (λ = 0.71073)
2Θ range for data collection/°	7.532 to 60.124°
Completeness to theta	0.995
Index ranges	$-2 \le h \le 6$, $-39 \le k \le 39$, $-7 \le l \le 7$
Reflections collected	11797
Independent reflections	4180 [R_{int} = 0.0380, R_{sigma} = 0.0471]
Data/restraints/parameters	4180/1/201
Goodness-of-fit on F^2	1.026
Final R indexes [I>=2σ (I)]	R_1 = 0.0441, wR_2 = 0.1014
Final R indexes [all data]	R_1 = 0.0569, wR_2 = 0.1097
Largest diff. peak/hole / e Å^{-3}	0.32/-0.23
Flack parameter	0.2(5)

Table S2. Bond lengths for compound 32.

Atom	Atom	Length/Å	Atom	Atom	Length/Å
O7	C2	1.214(3)	C5	C6	1.375(3)
O10	C4	1.244(3)	C8	C9	1.523(4)
O13	C12	1.237(3)	C12	C14	1.511(3)
N1	C2	1.381(3)	C14	C15	1.522(3)
N1	C6	1.369(3)	C15	C16	1.515(3)
N3	C2	1.383(3)	C16	C17	1.381(4)
N3	C4	1.396(3)	C16	C21	1.386(4)
N3	C8	1.485(3)	C17	C18	1.391(4)
N11	C5	1.420(3)	C18	C19	1.355(5)
N11	C12	1.351(3)	C19	C20	1.384(5)
N22	C6	1.351(3)	C20	C21	1.387(4)
C4	C5	1.425(3)			

Table S3 Bond angles for compound 32.

Atom	Atom	Atom	Angle/°	Atom	Atom	Atom	Angle/°
C6	N1	C2	124.5(2)	N22	C6	C5	124.6(2)
C2	N3	C4	123.6(2)	N3	C8	C9	112.4(2)
C2	N3	C8	116.5(2)	O13	C12	N11	122.4(2)
C4	N3	C8	119.9(2)	O13	C12	C14	123.2(2)
C12	N11	C5	123.2(2)	N11	C12	C14	114.4(2)
O7	C2	N1	121.0(2)	C12	C14	C15	114.0(2)
O7	C2	N3	123.5(2)	C16	C15	C14	112.8(2)
N1	C2	N3	115.5(2)	C17	C16	C15	120.5(3)
O10	C4	N3	120.1(2)	C17	C16	C21	117.7(3)
O10	C4	C5	123.0(2)	C21	C16	C15	121.7(2)
N3	C4	C5	116.9(2)	C16	C17	C18	121.2(3)
N11	C5	C4	119.5(2)	C19	C18	C17	120.8(3)
C6	C5	N11	119.8(2)	C18	C19	C20	118.9(3)
C6	C5	C4	120.6(2)	C19	C20	C21	120.7(3)
N1	C6	C5	118.5(2)	C16	C21	C20	120.6(3)
N22	C6	N1	116.8(2)				

Table S4. Hydrogen bonds for compound 32.

D	H	A	d(D-H)/Å	d(H-A)/Å	d(D-A)/Å	D-H-A/°
N1	H1	O10[1]	0.88	1.90	2.717(3)	153.7
N11	H11	O13[2]	0.88	2.39	2.975(3)	123.7
N22	H22A	O10[1]	0.86	2.18	2.875(3)	137.5
N22	H22B	O13[2]	0.86	2.14	2.994(3)	170.5
C9	H9B	O7[3]	0.98	2.31	3.276(3)	168.6

[1]-1+X,+Y,-1+Z; [2]-1+X,+Y,+Z; [3]+X,+Y,1+Z

^{1}H- and ^{13}C-NMR spectra of compounds 21-

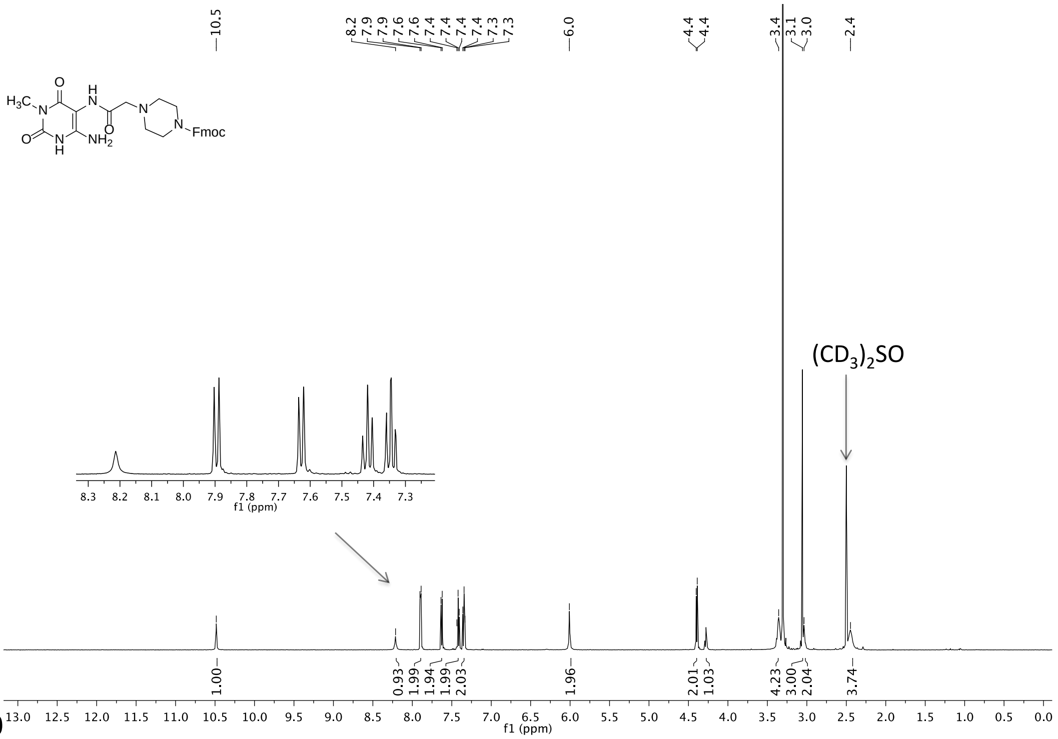

39

Figure S1. ^{1}H-NMR spectrum of 21 in $(CD_3)_2SO$ at room temperature.

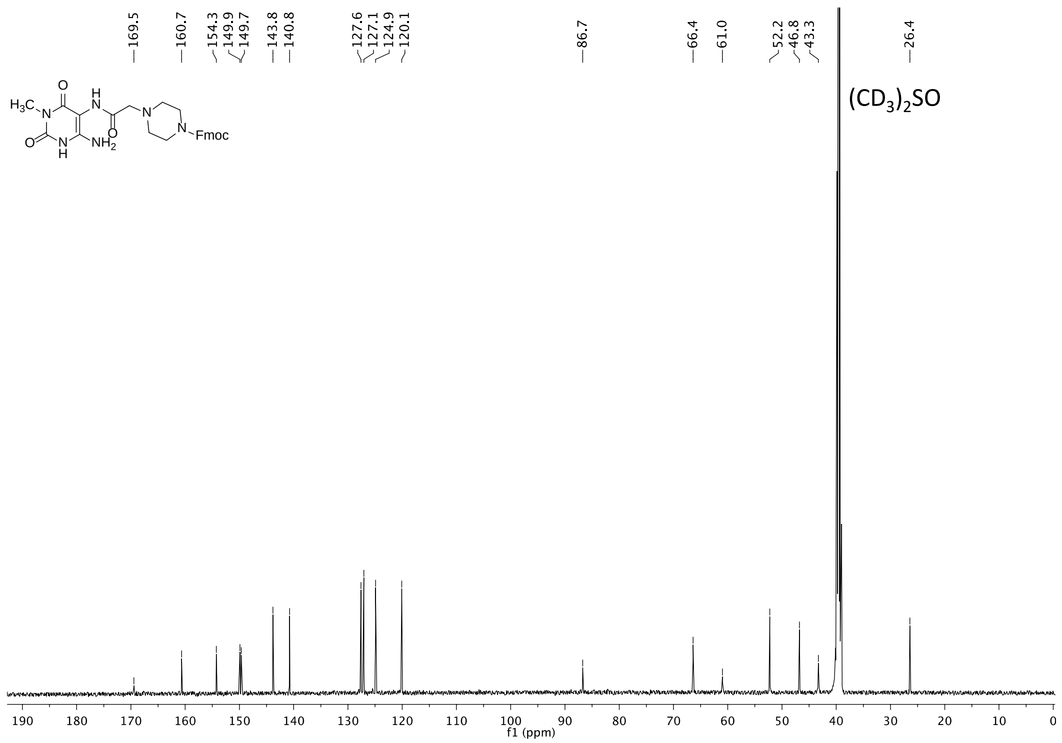

Figure S2. ^{13}C-NMR spectrum of 21 in $(CD_3)_2SO$ at room temperature

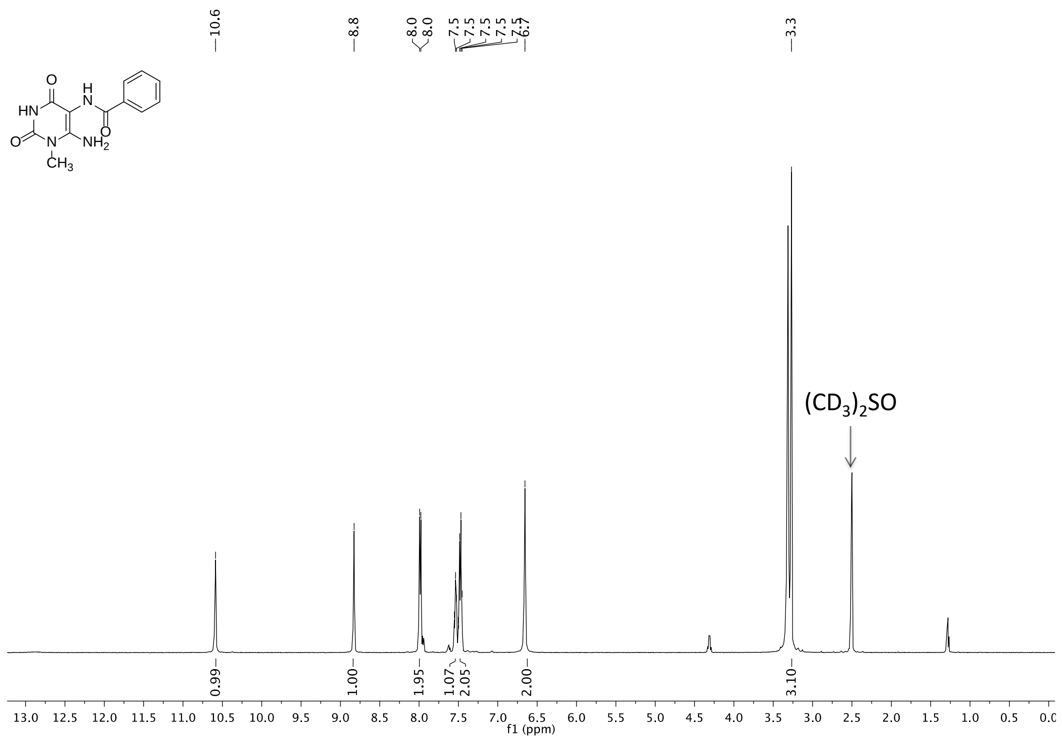

Figure S3. ^{1}H-NMR spectrum of 22 in $(CD_3)_2SO$ at room temperature.

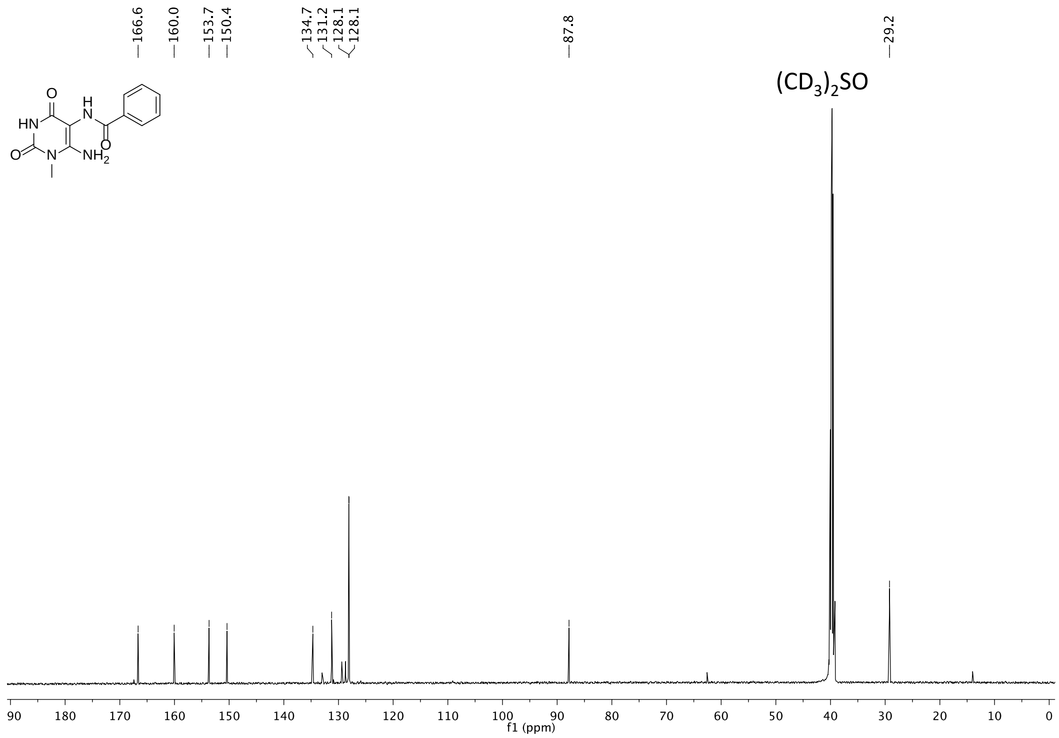

Figure S4. ^{13}C-NMR spectrum of 22 in $(CD_3)_2SO$ at room temperature.

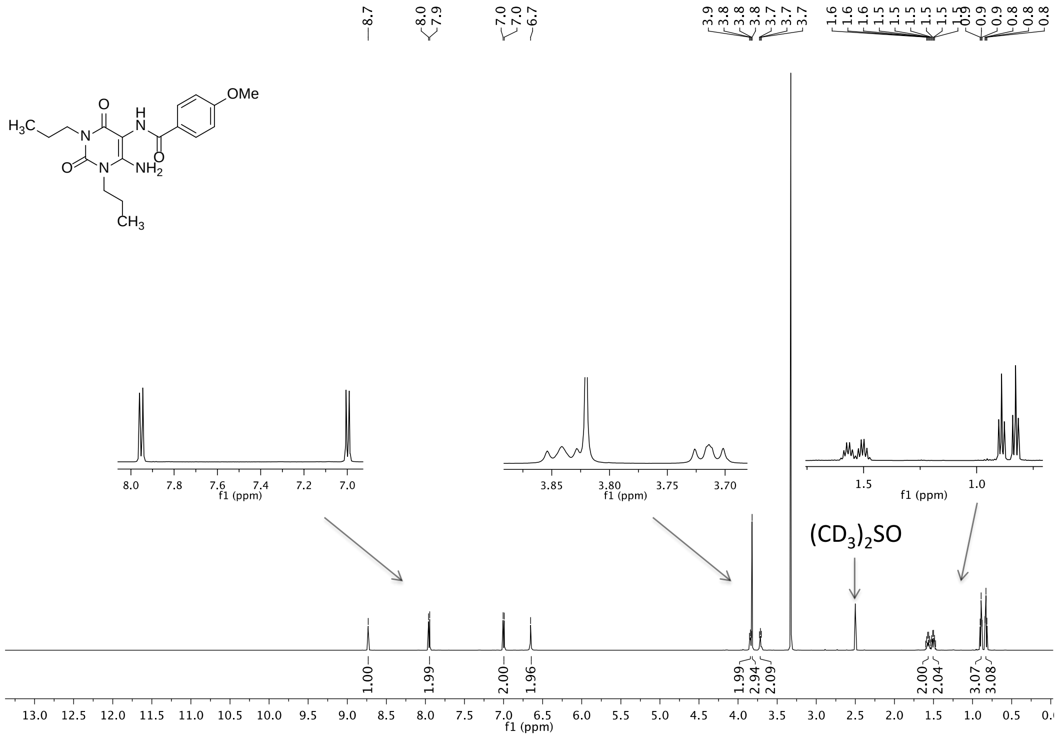

Figure S5. ^{1}H-NMR spectrum of 23 in $(CD_3)_2SO$ at room temperature.

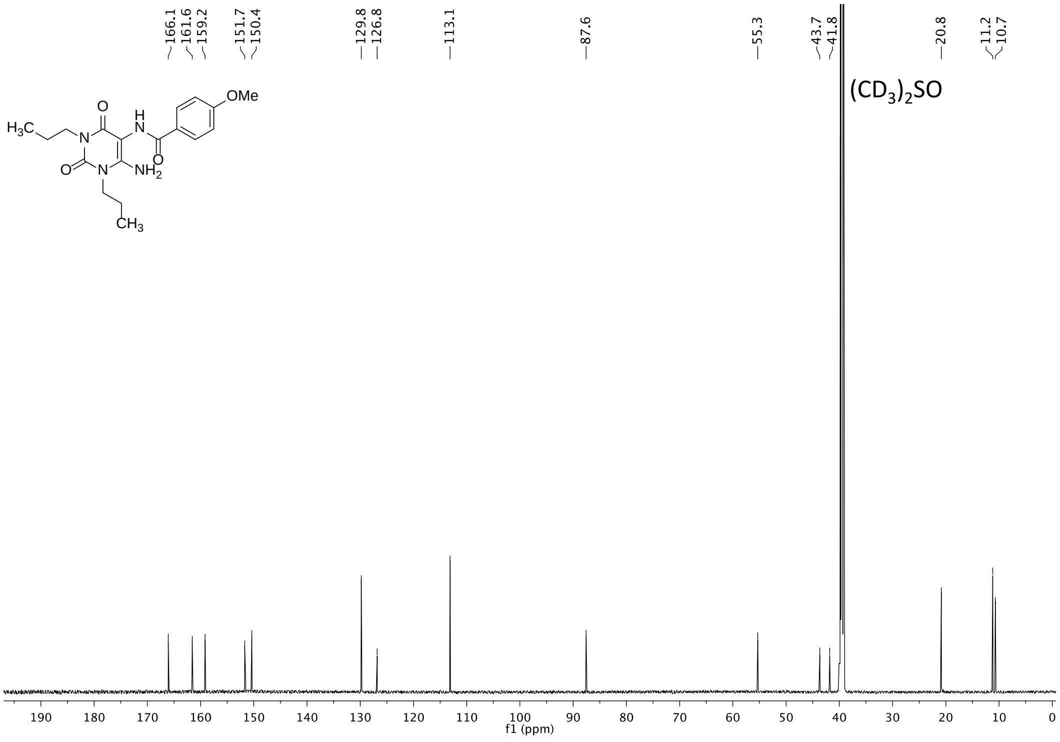

Figure S6. [13]**C-NMR spectrum of 23 in** $(CD_3)_2SO$ **at room temperature.**

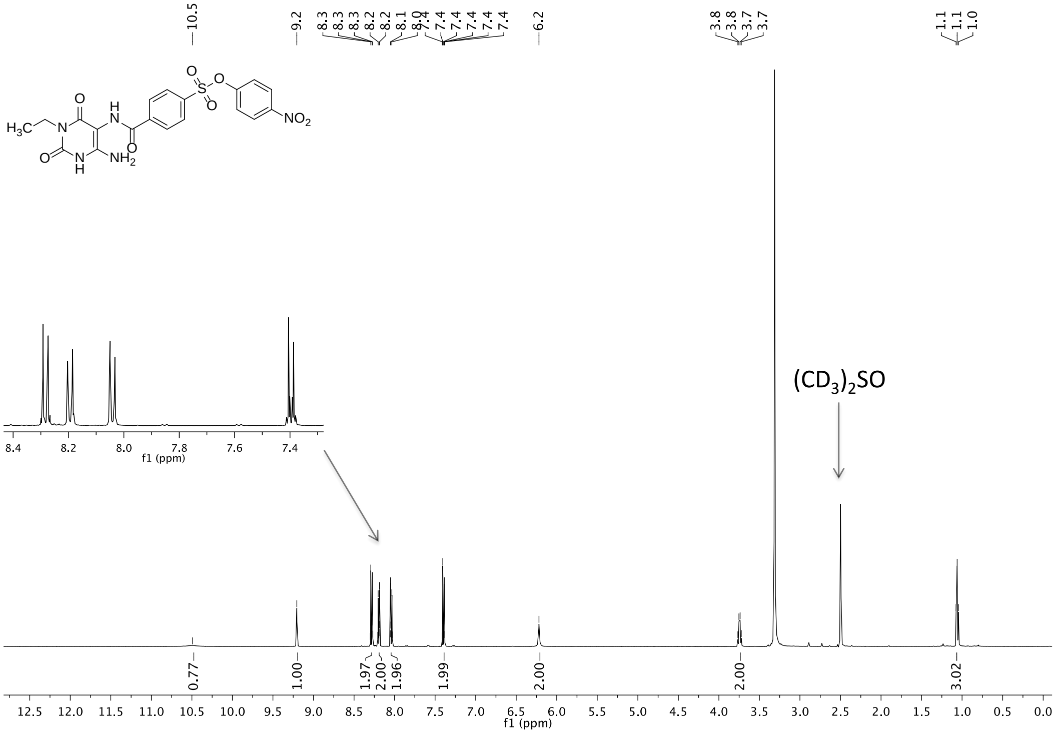

Figure S7. [1]**H-NMR spectrum of 24 in** $(CD_3)_2SO$ **at room temperature.**

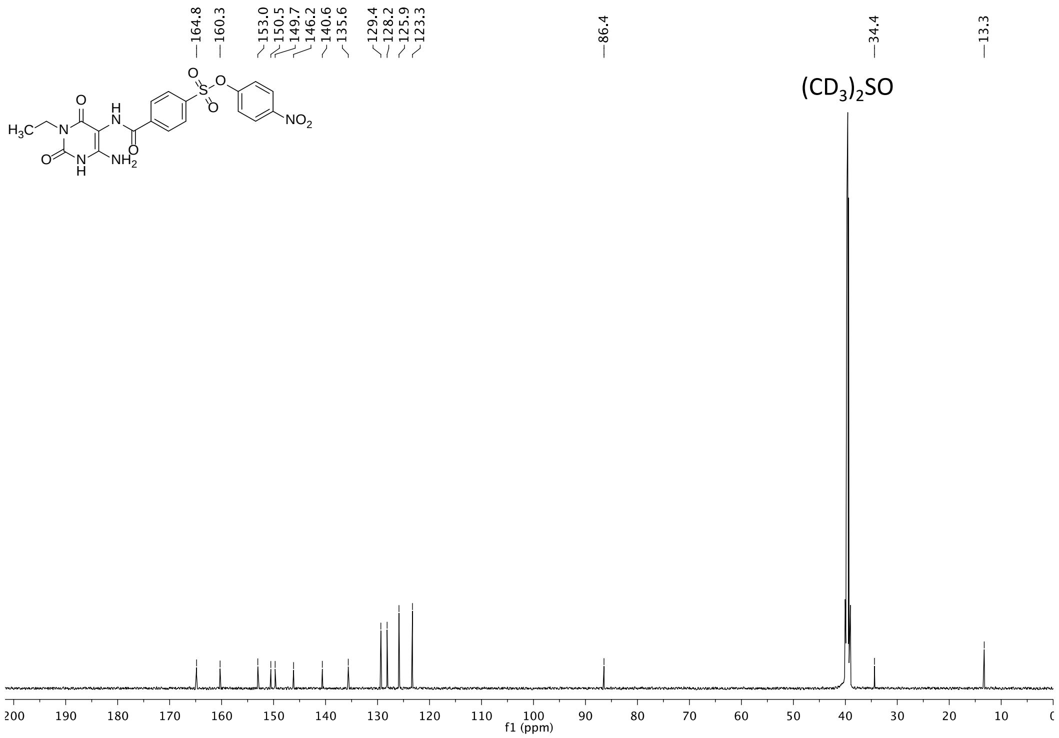

Figure S8. **^{13}C-NMR spectrum of 24 in $(CD_3)_2SO$ at room temperature.**

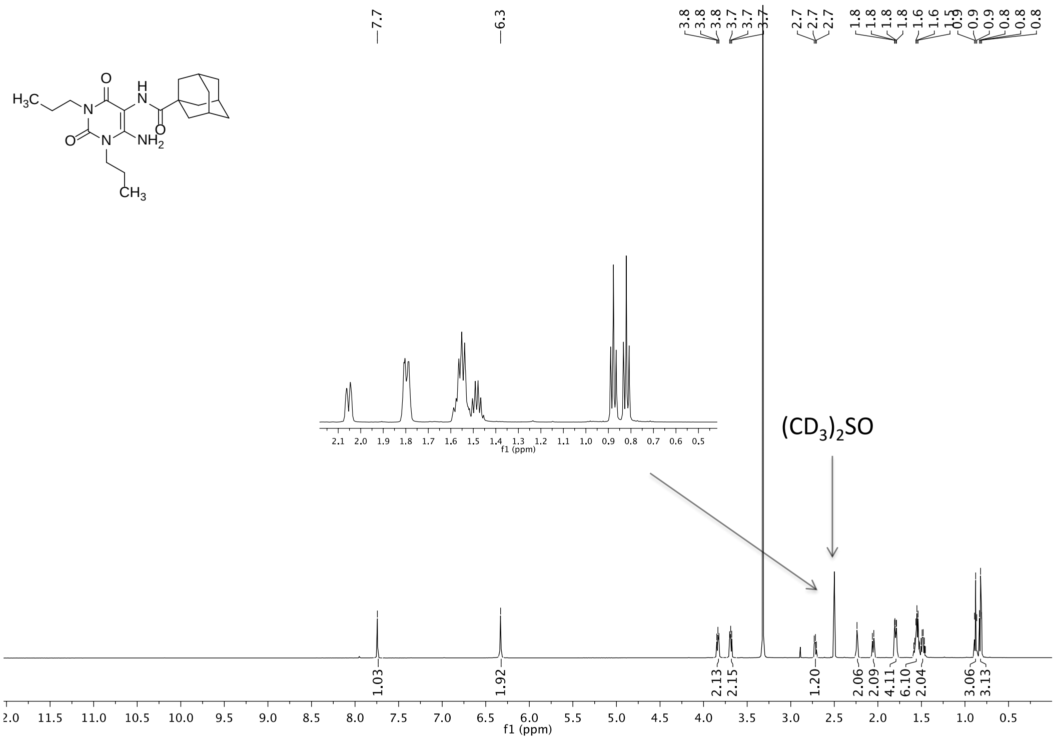

Figure S9. **^{1}H-NMR spectrum of 25 in $(CD_3)_2SO$ at room temperature.**

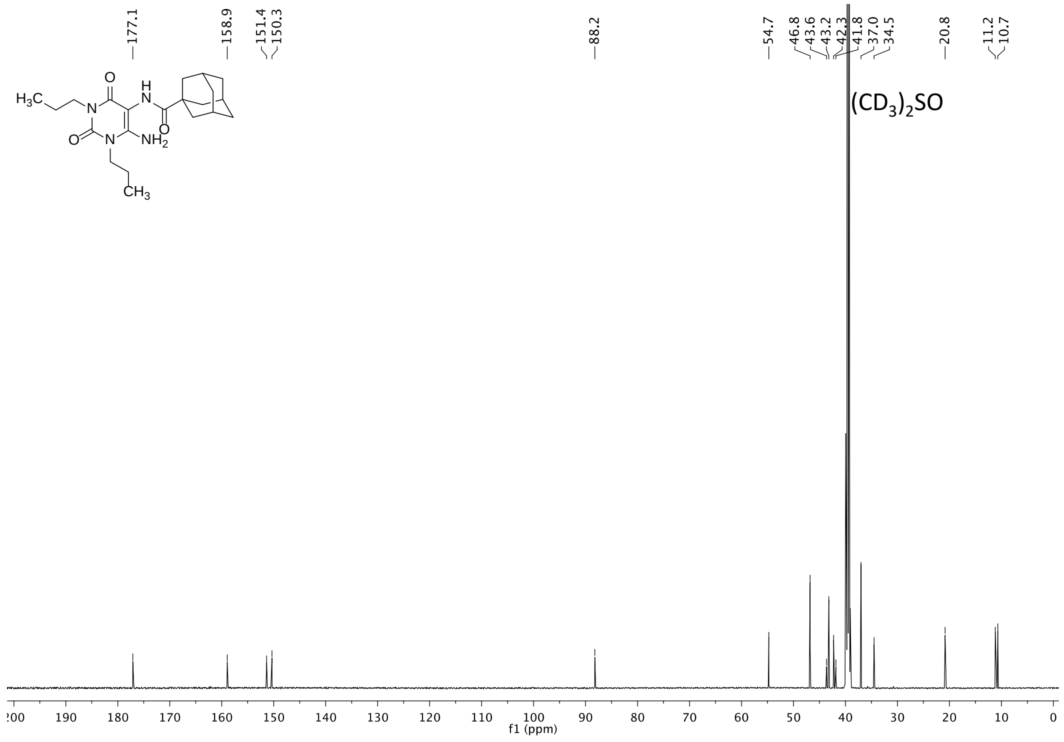

Figure S10. ^{13}**C-NMR spectrum of 25 in** $(CD_3)_2SO$ **at room temperature.**

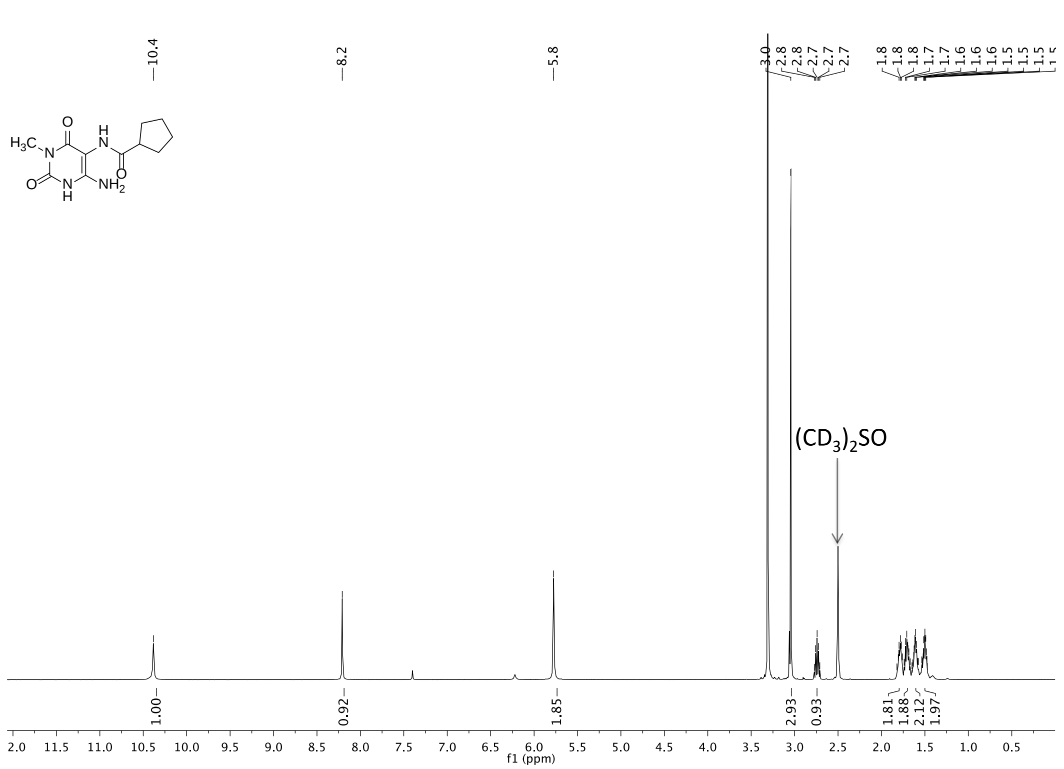

Figure S11. ^{1}**H-NMR spectrum of 26 in** $(CD_3)_2SO$ **at room temperature.**

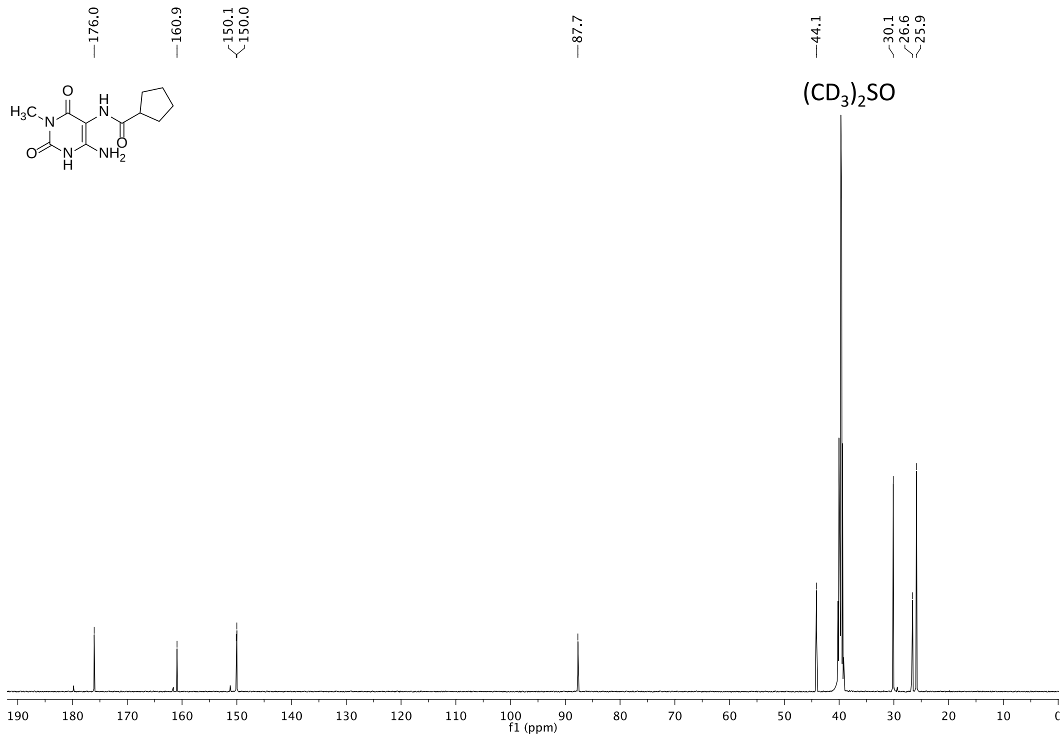

Figure S12. ^{13}C-NMR spectrum of 26 in $(CD_3)_2SO$ at room temperature.

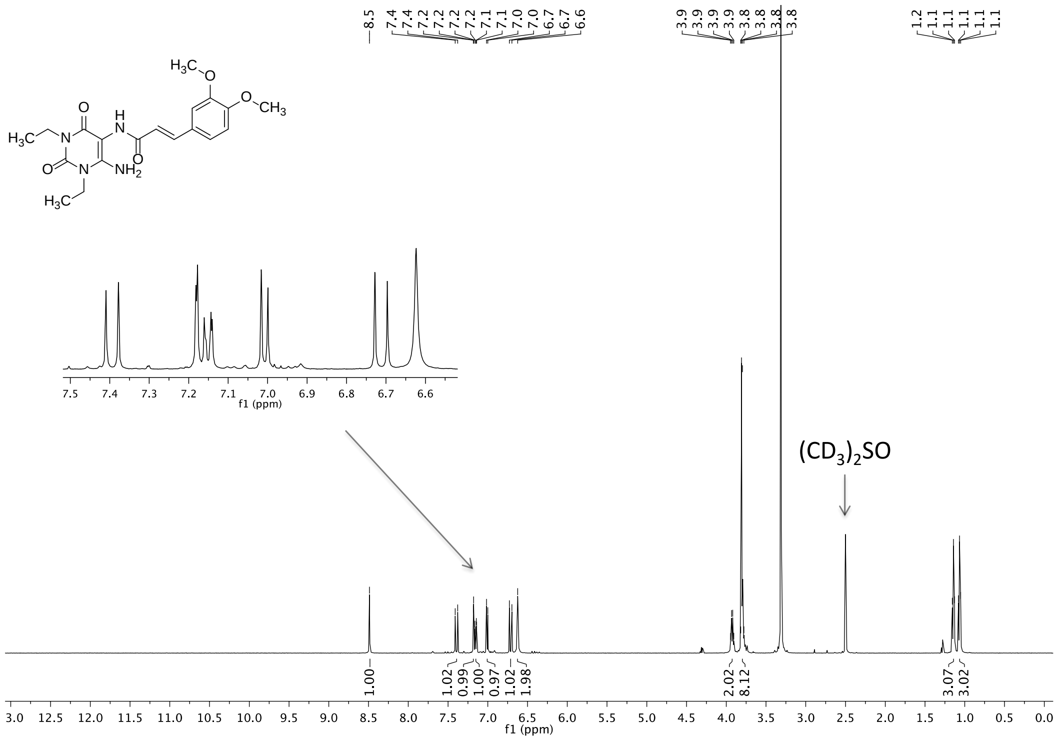

Figure S13. ^{1}H-NMR spectrum of 27 in $(CD_3)_2SO$ at room temperature.

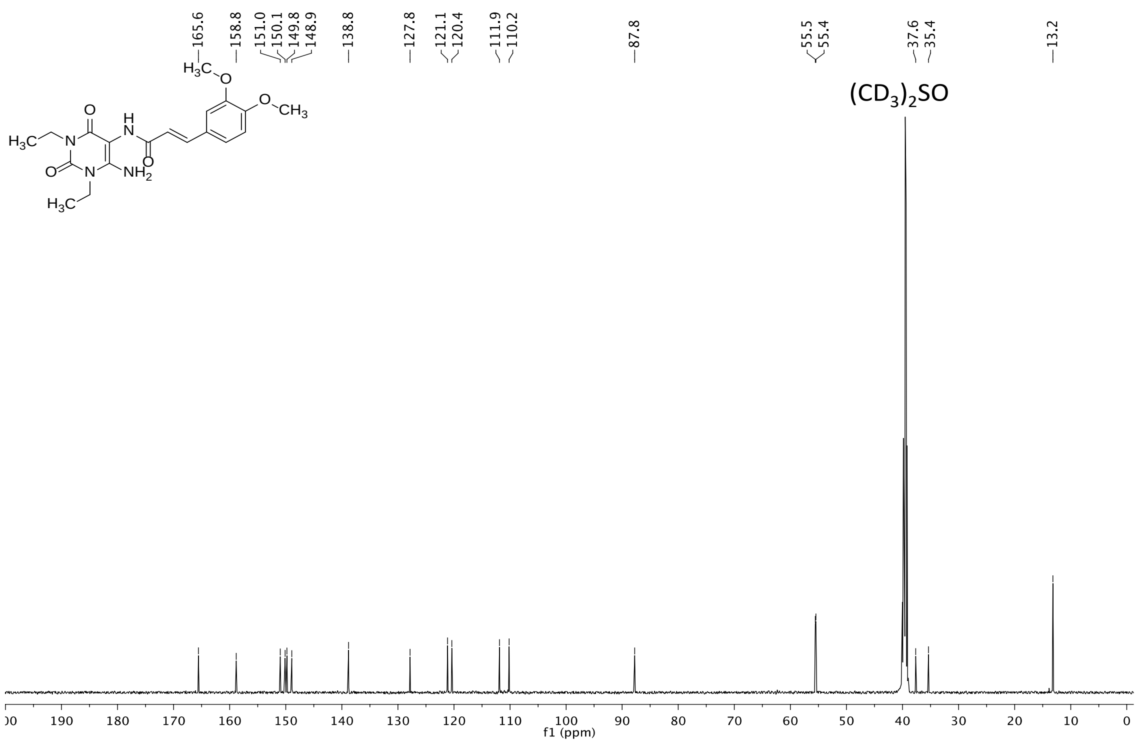

Figure S14. ^{13}**C-NMR spectrum of 27 in** $(CD_3)_2SO$ **at room temperature.**

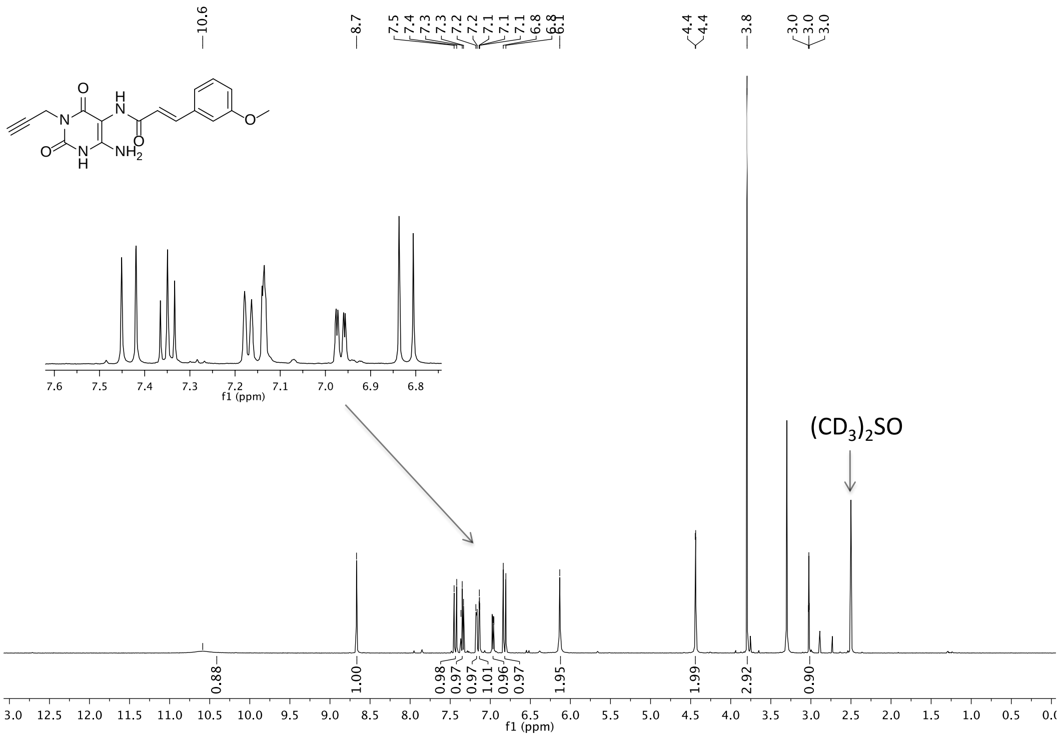

Figure S15. ^{1}**H-NMR spectrum of 28 in** $(CD_3)_2SO$ **at room temperature.**

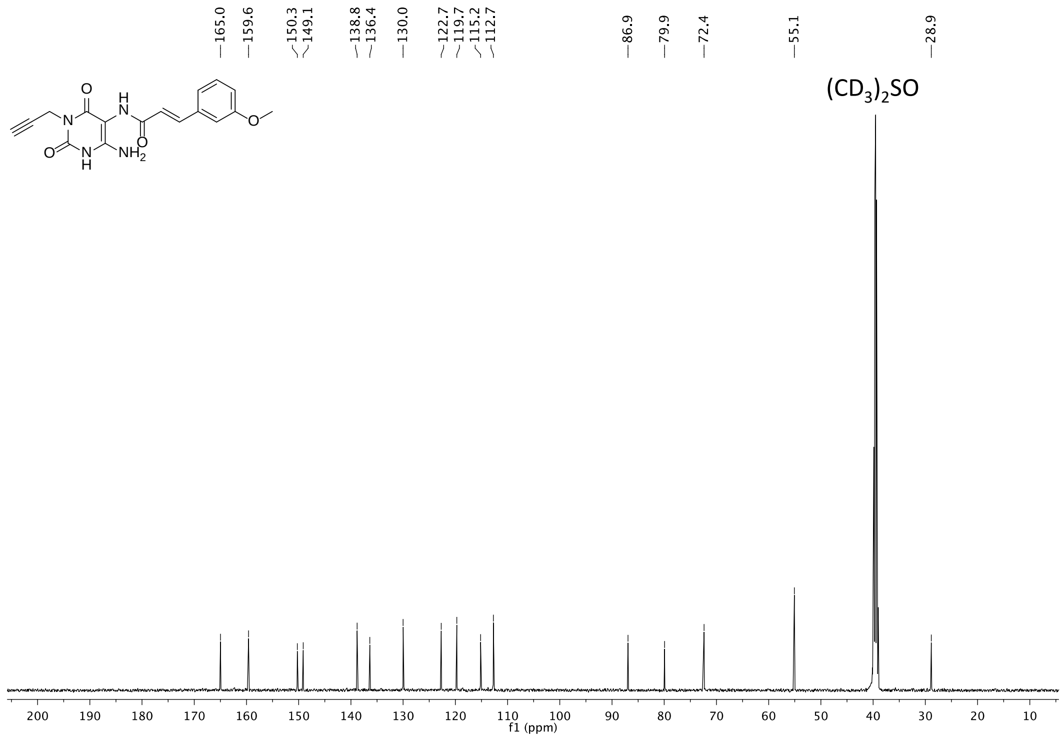

Figure S16. ^{13}C-NMR spectrum of 28 in $(CD_3)_2SO$ at room temperature.

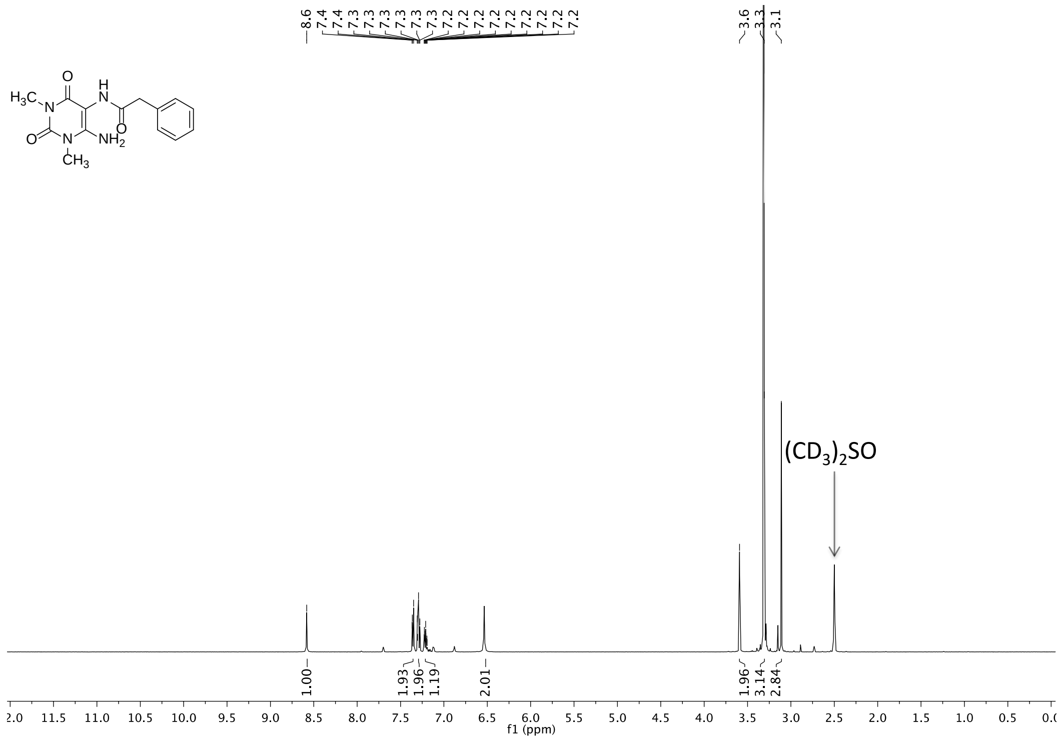

Figure S17. ^{1}H-NMR spectrum of 29 in $(CD_3)_2SO$ at room temperature.

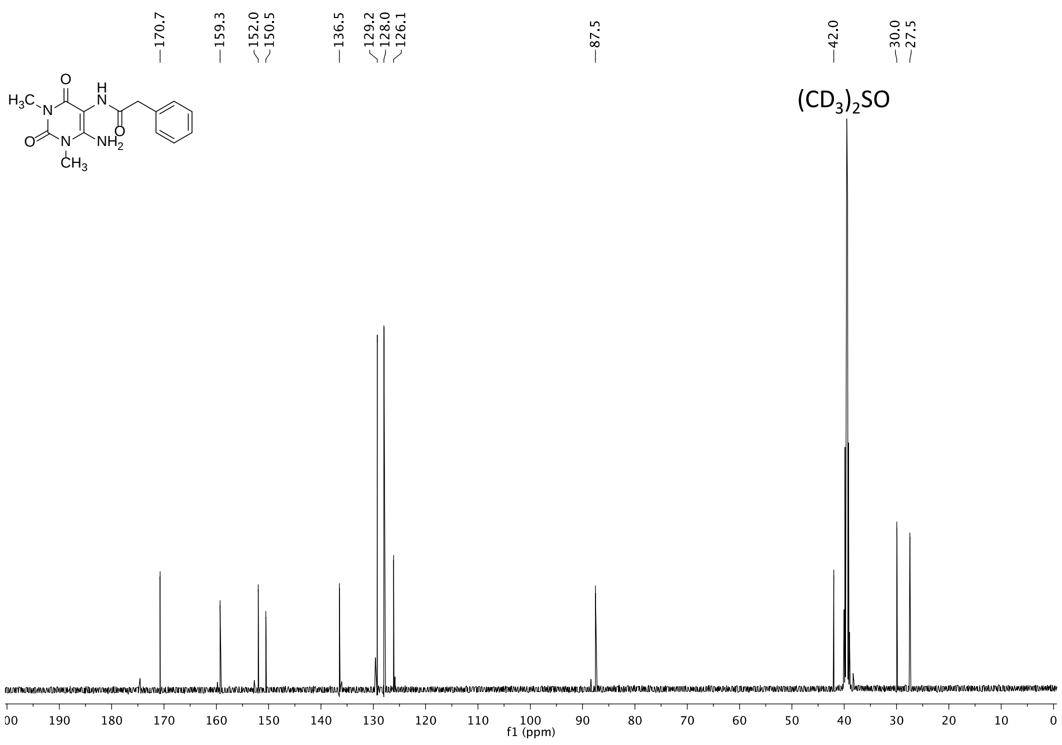

Figure S18. ^{13}C-NMR spectrum of 29 in $(CD_3)_2SO$ at room temperature.

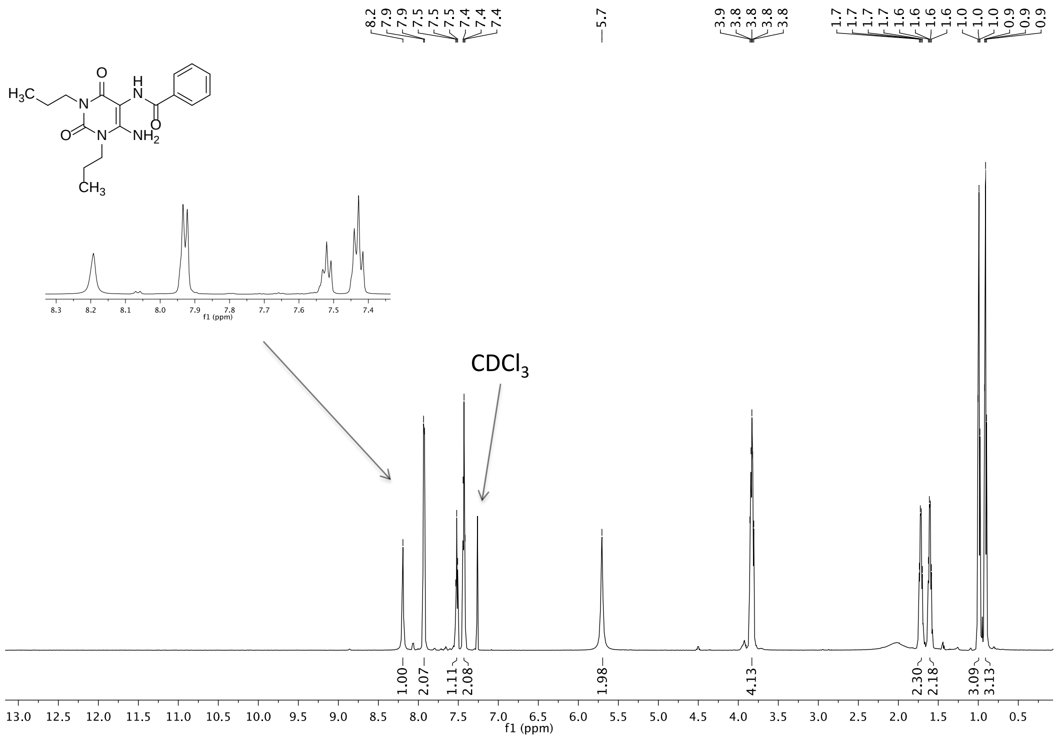

Figure S19. ^{1}H-NMR spectrum of 30 in $(CD_3)_2SO$ at room temperature.

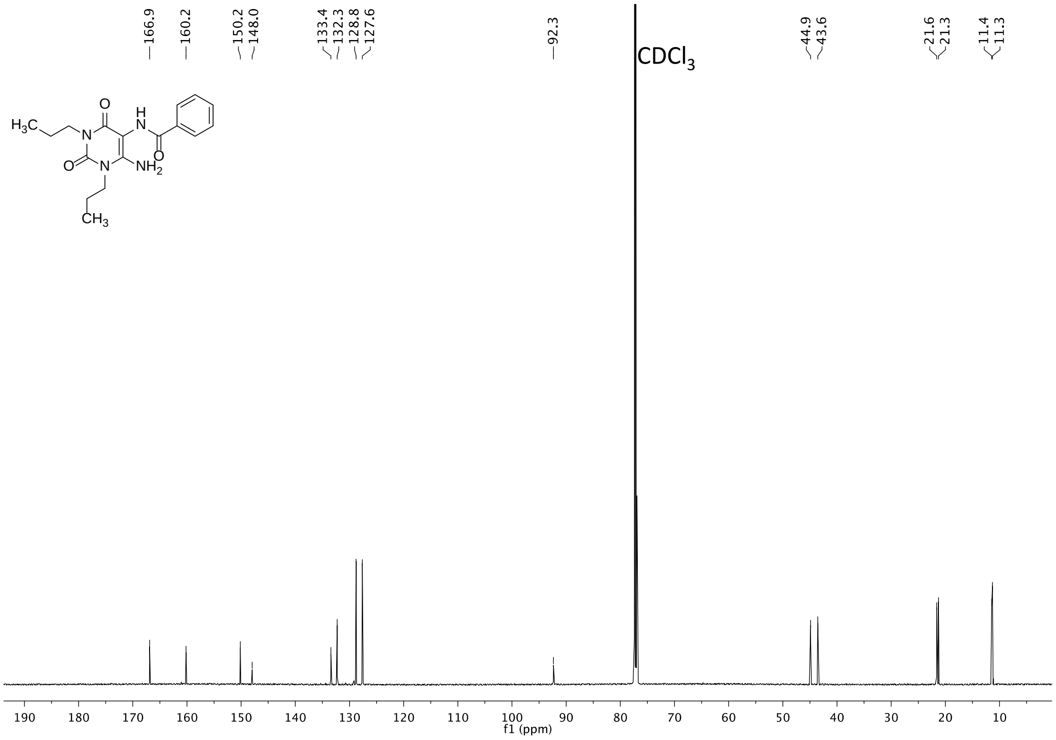

Figure S20. ^{13}C-NMR spectrum of 30 in $(CD_3)_2SO$ at room temperature.

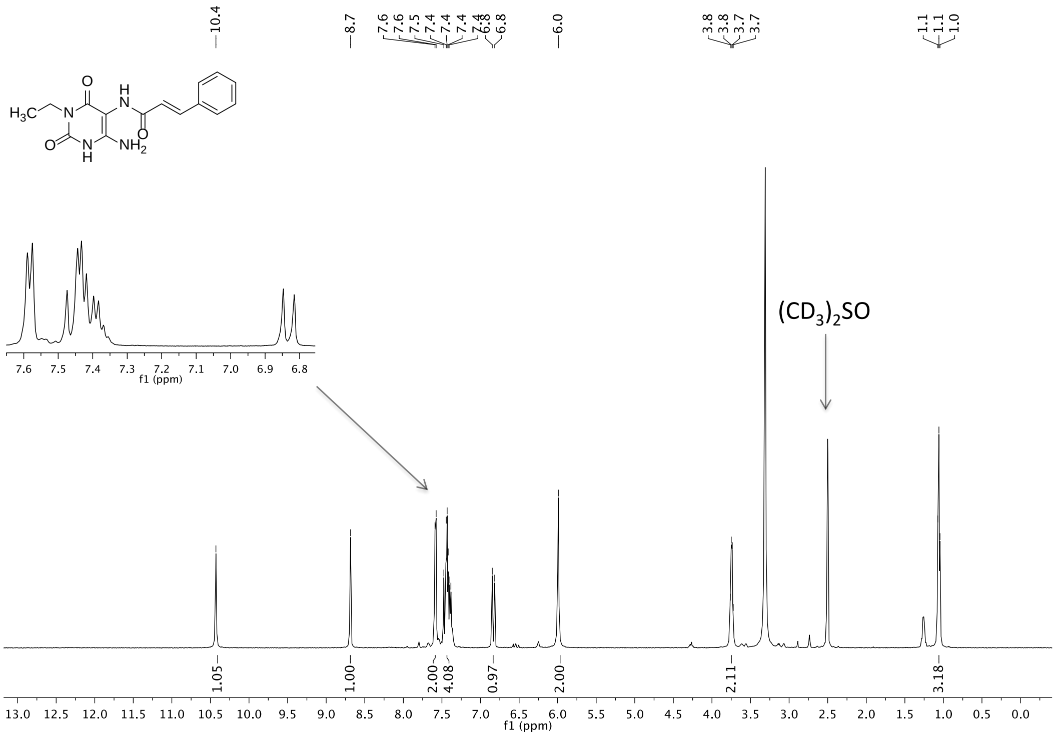

Figure S21. ^{1}H-NMR spectrum of 31 in $(CD_3)_2SO$ at room temperature.

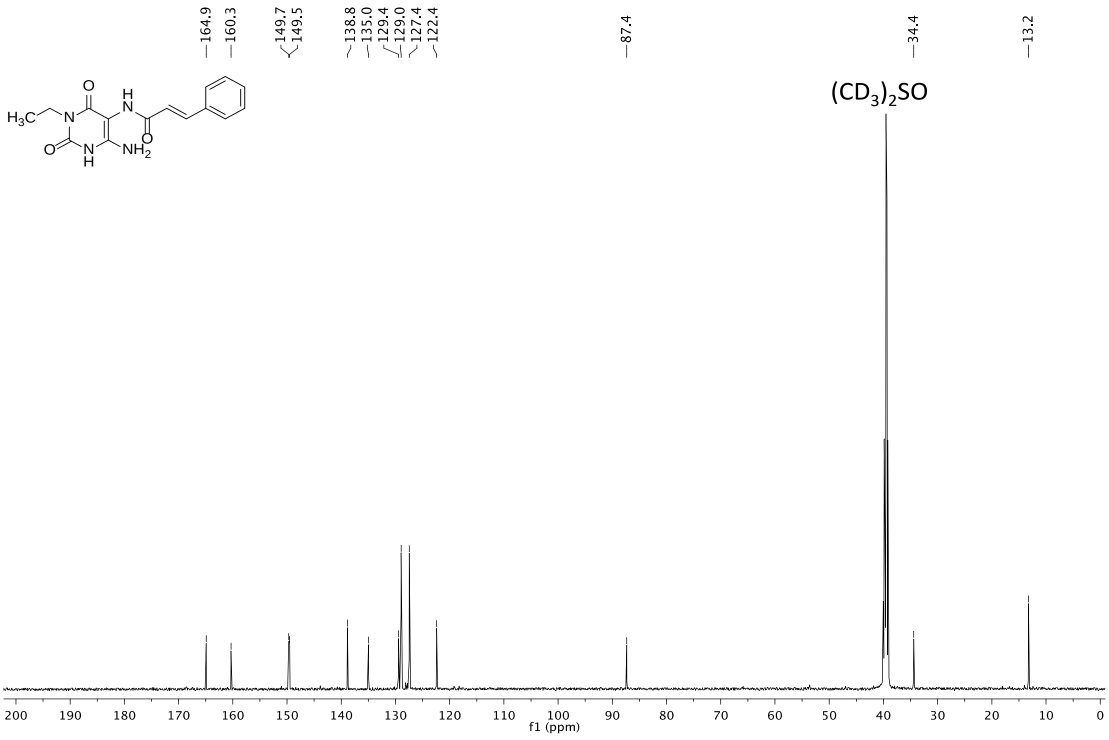

Figure S22. ^{13}**C-NMR spectrum of 31 in** $(CD_3)_2SO$ **at room temperature.**

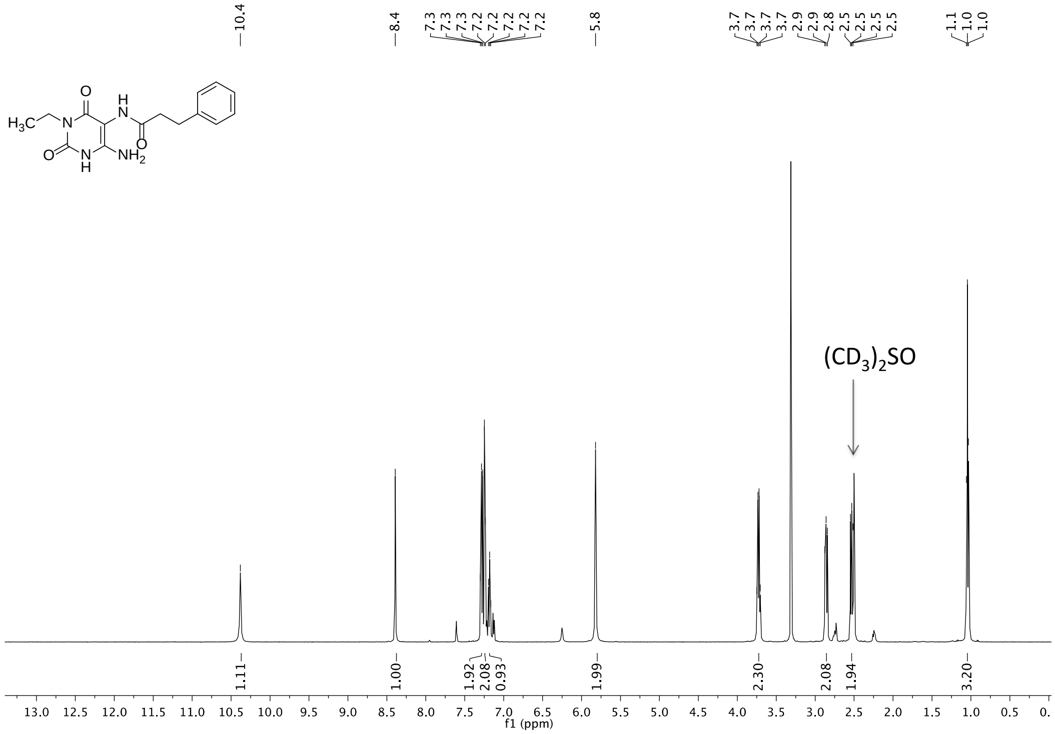

Figure S23. ^{1}**H-NMR spectrum of 32 in** $(CD_3)_2SO$ **at room temperature.**

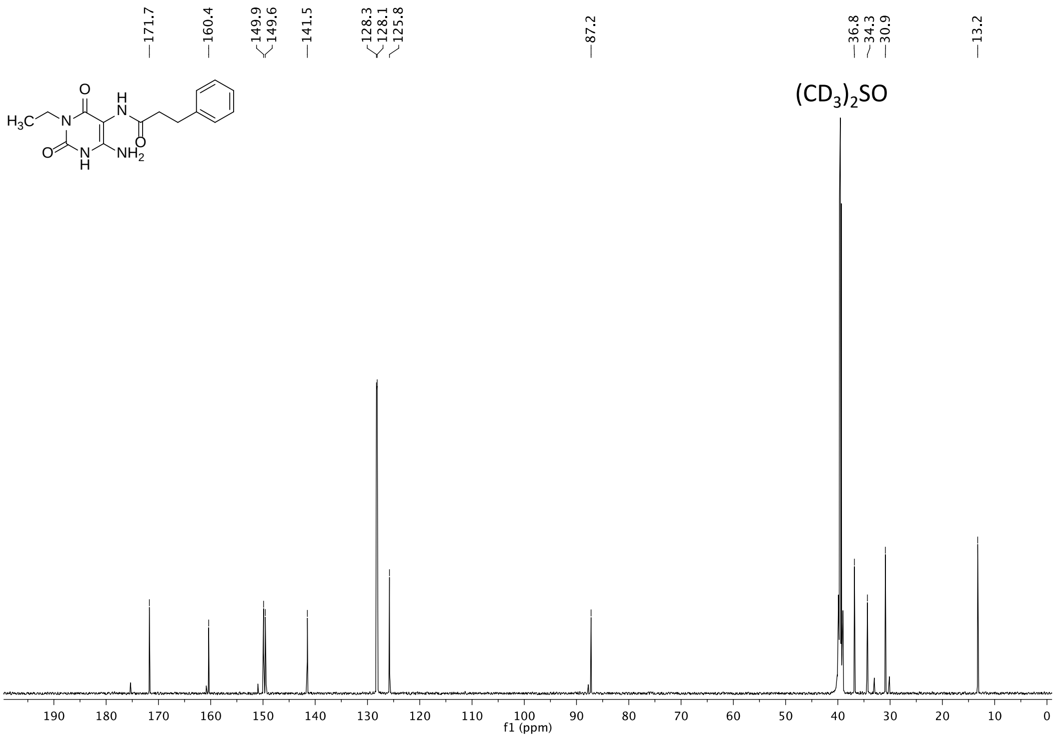

Figure S24. ^{13}C-NMR spectrum of 32 in $(CD_3)_2SO$ at room temperature.

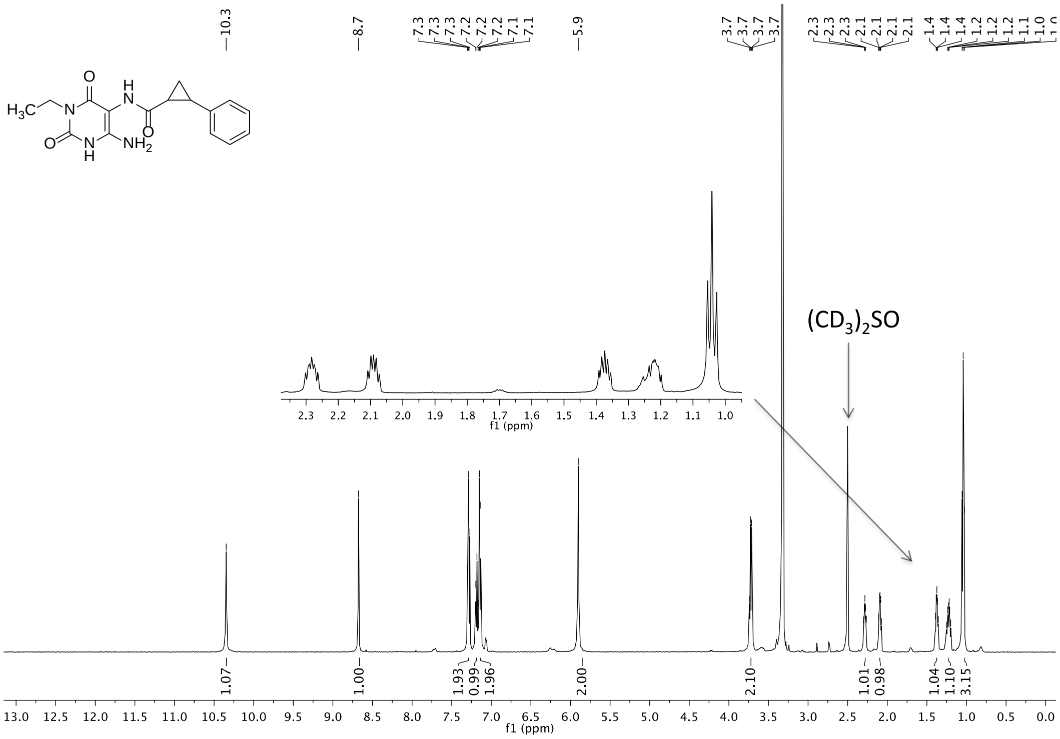

Figure S25. ^{13}C-NMR spectrum of 33 in $(CD_3)_2SO$ at room temperature.

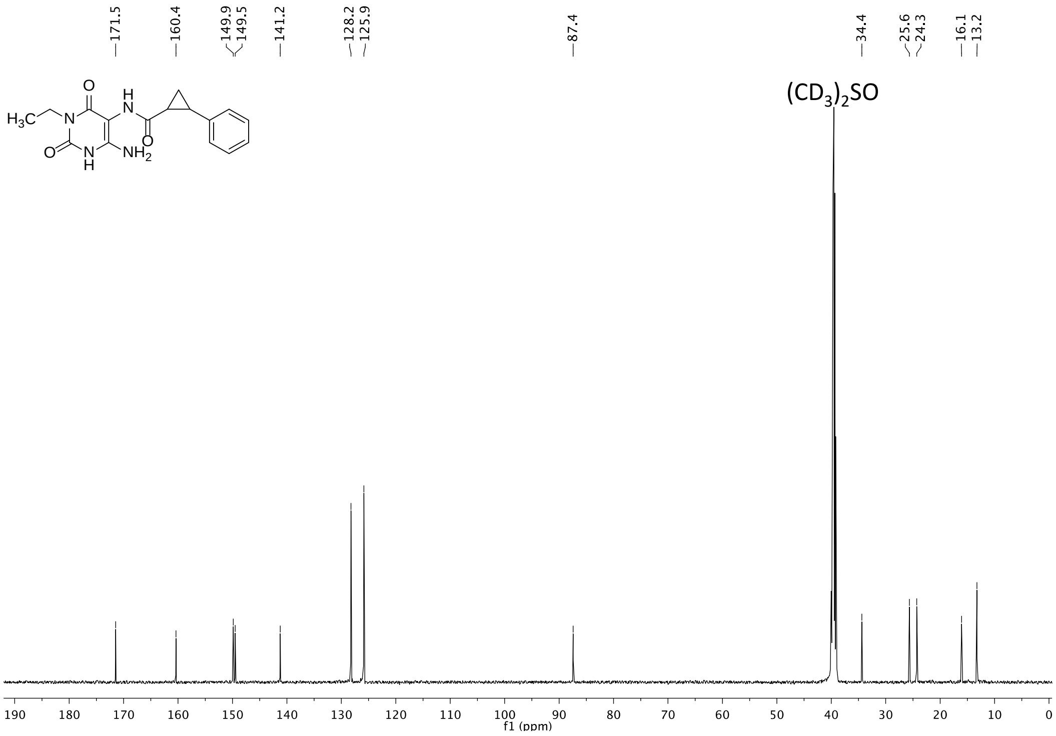

Figure S26. ^{13}**C-NMR spectrum of 33 in** $(CD_3)_2SO$ **at room temperature.**

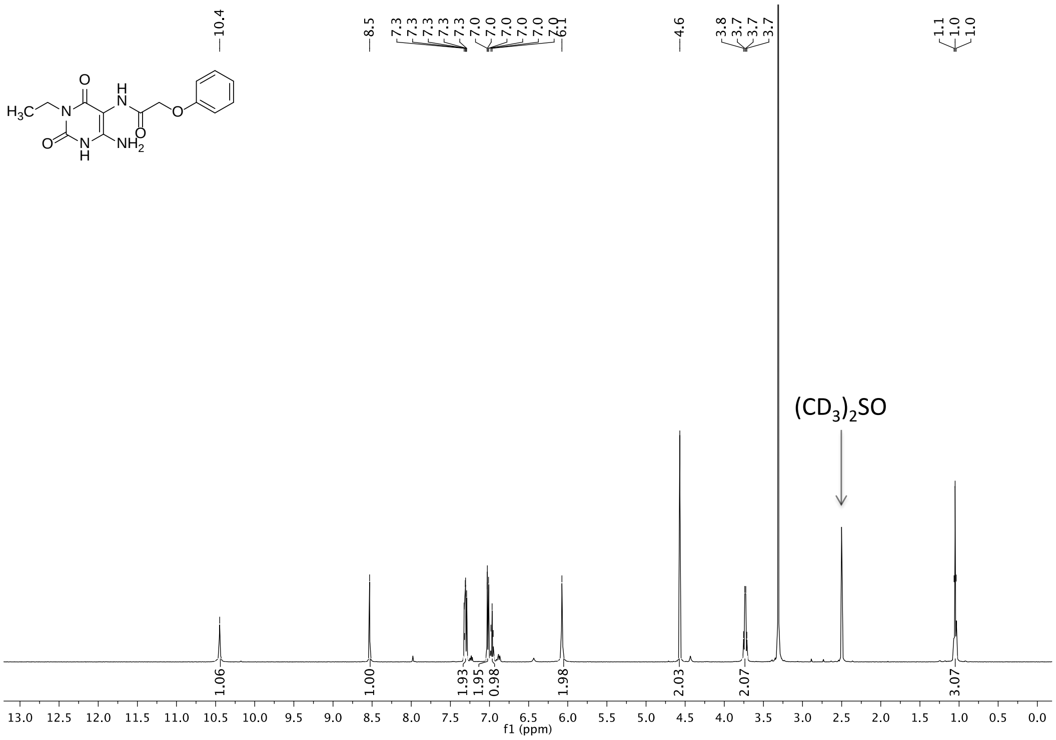

Figure S27. ^{1}**H-NMR spectrum of 34 in** $(CD_3)_2SO$ **at room temperature.**

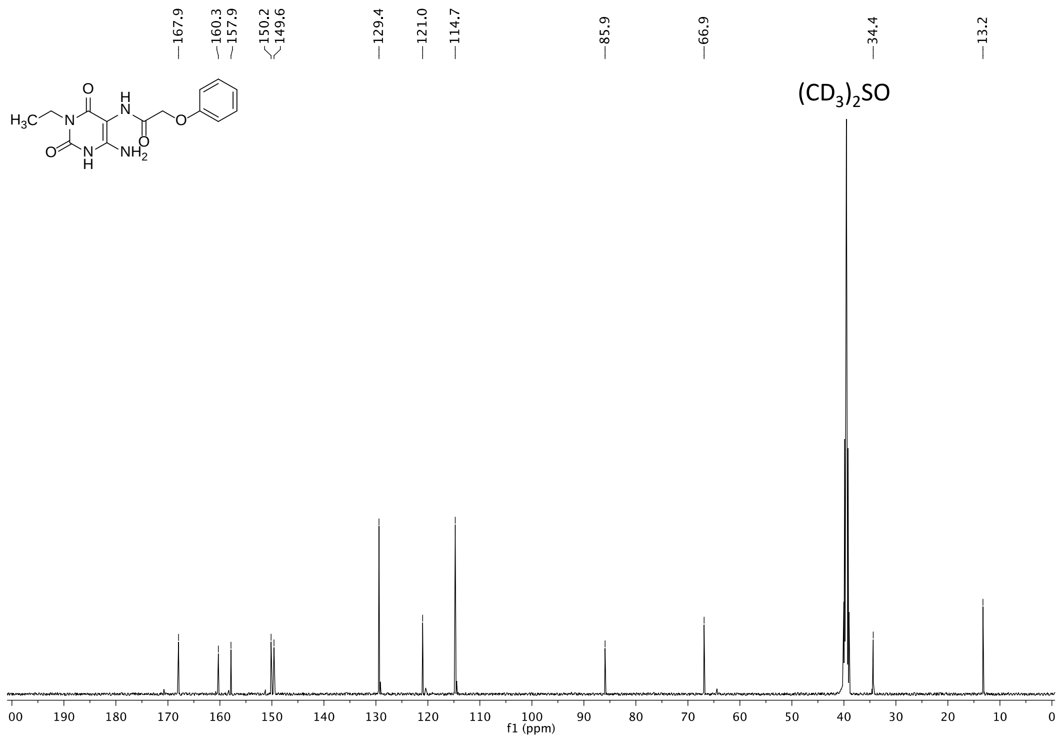

Figure S28. ^{13}C-NMR spectrum of 34 in $(CD_3)_2SO$ at room temperature.

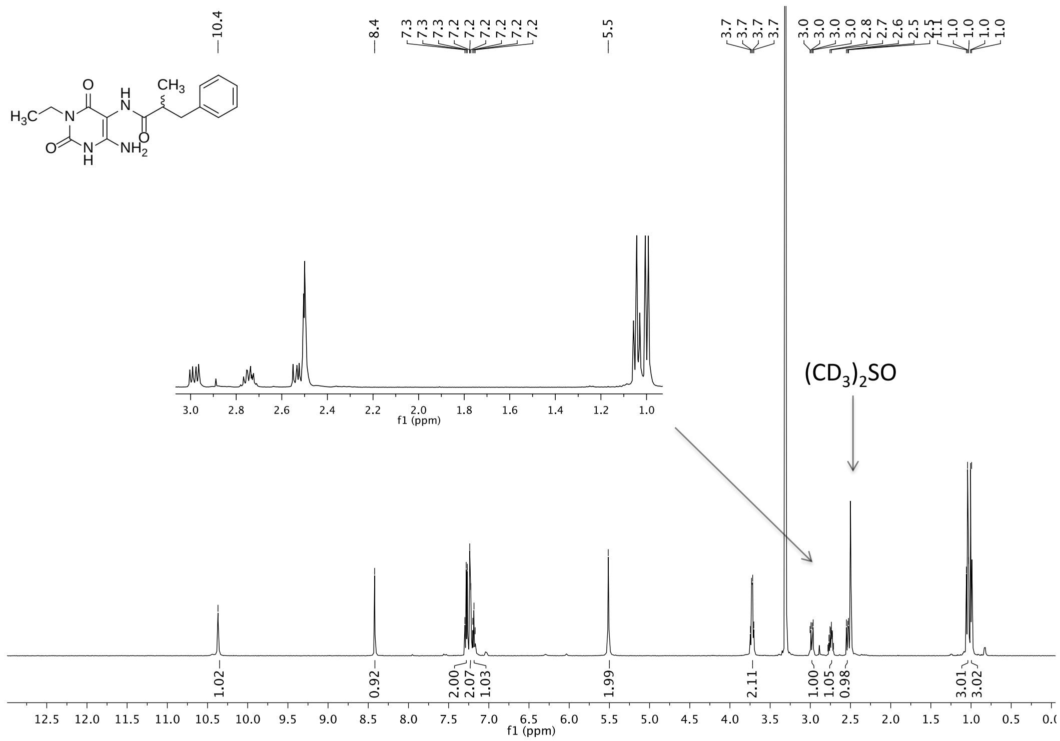

Figure S29. ^{1}H-NMR spectrum of 35 in $(CD_3)_2SO$ at room temperature.

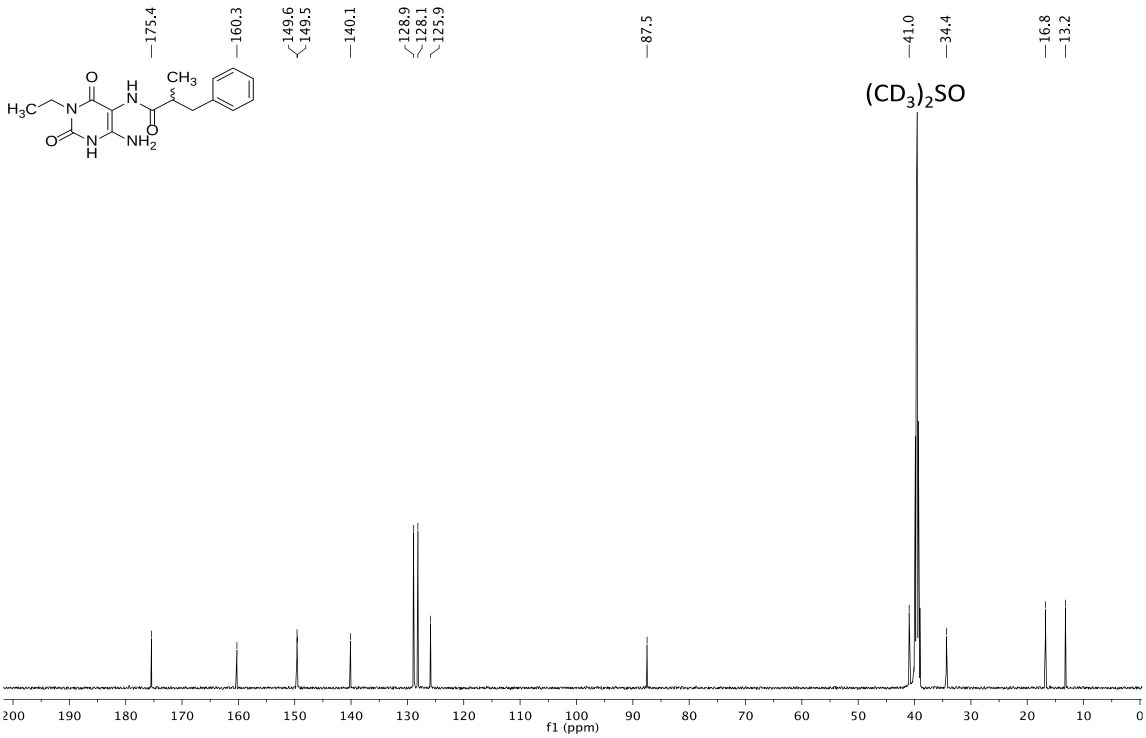

Figure S30. ^{13}C-NMR spectrum of 35 in $(CD_3)_2SO$ at room temperature.

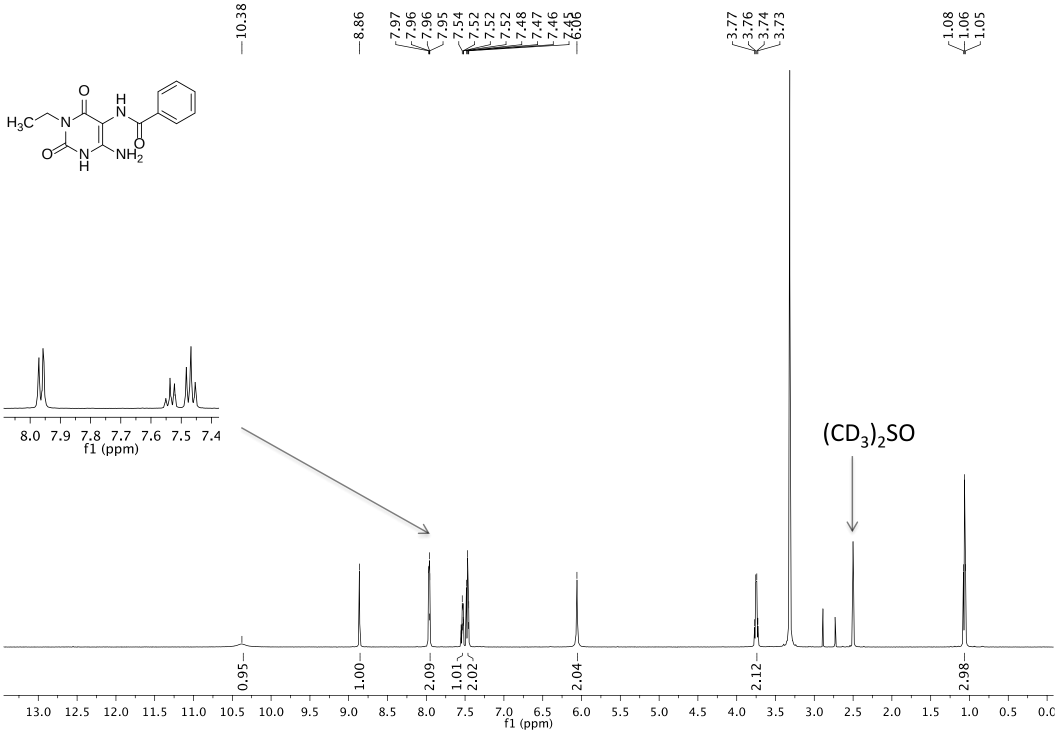

Figure S31. ^{1}H-NMR spectrum of 36 in $(CD_3)_2SO$ at room temperature.

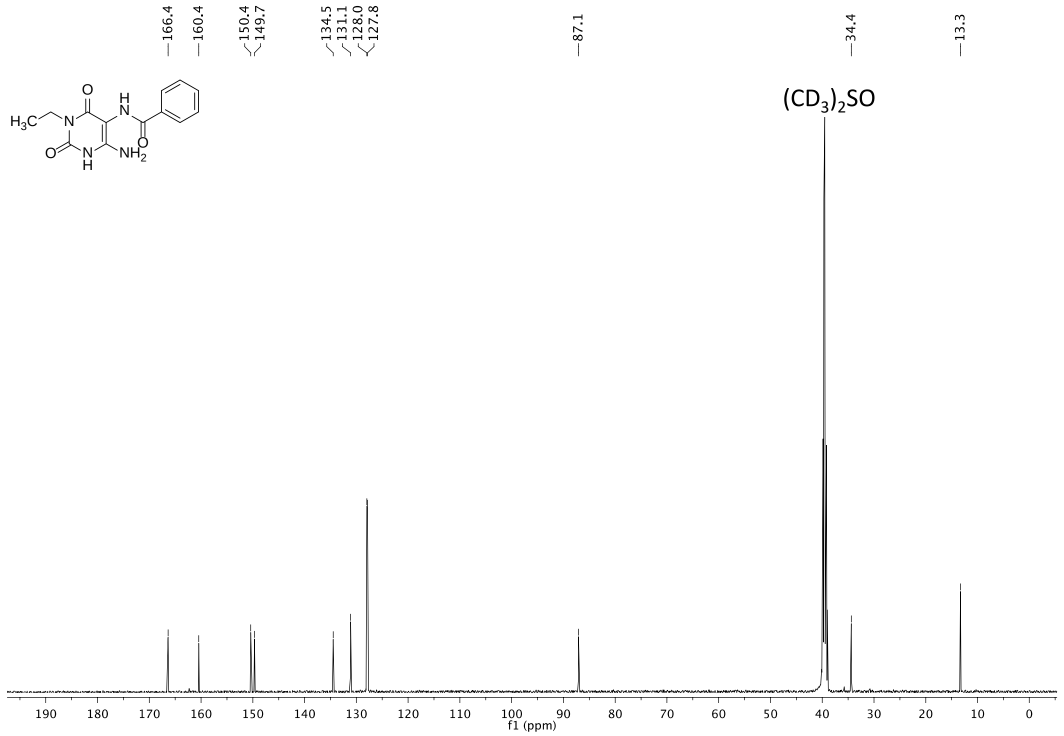

Figure S32 ^{13}C-NMR spectrum of 36 in $(CD_3)_2SO$ at room temperature.

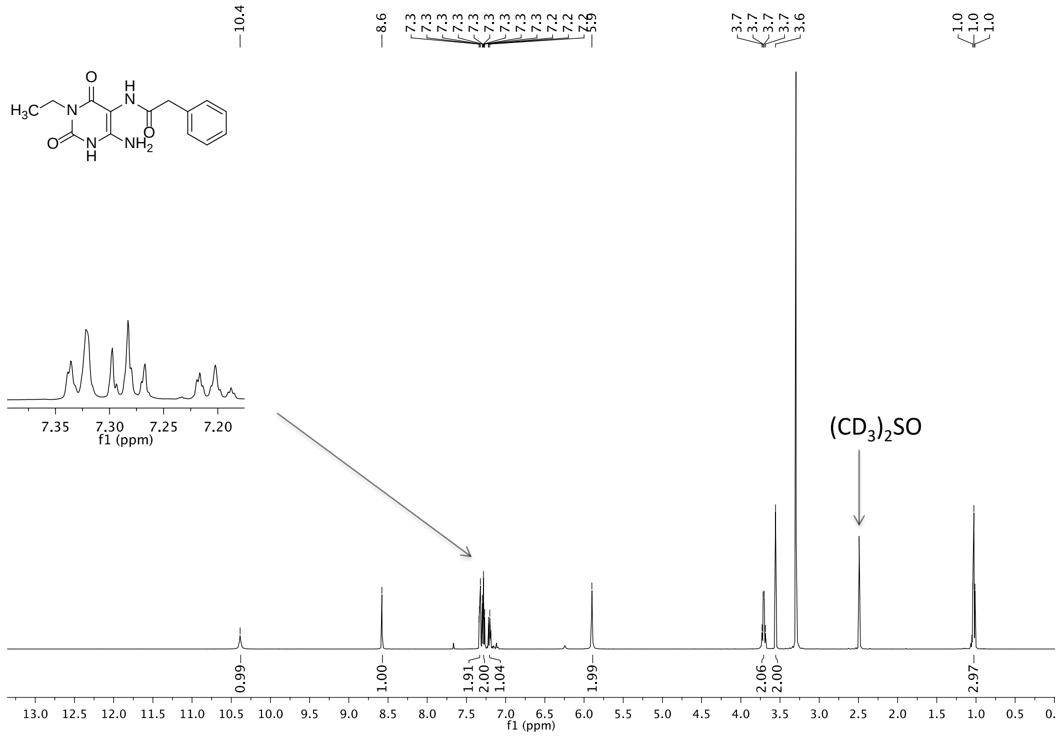

Figure S33. ^{1}H-NMR spectrum of 37 in $(CD_3)_2SO$ at room temperature.

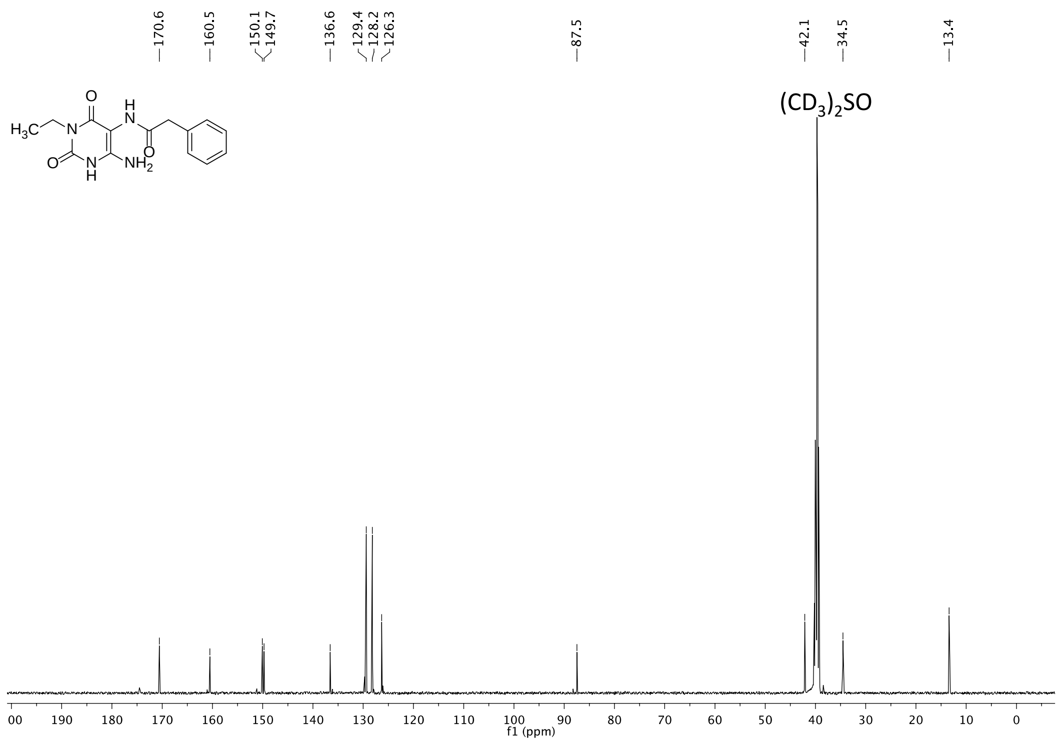

Figure S34. ^{13}C-NMR spectrum of 37 in $(CD_3)_2SO$ at room temperature.

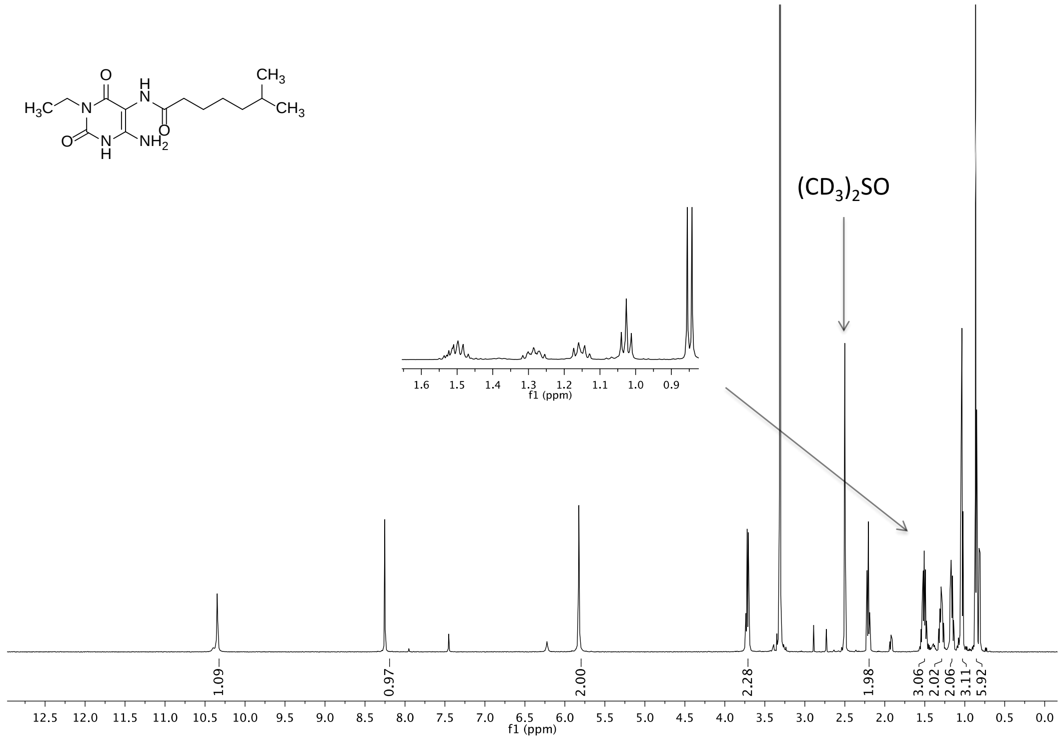

Figure S35. ^{1}H-NMR spectrum of 38 in $(CD_3)_2SO$ at room temperature.

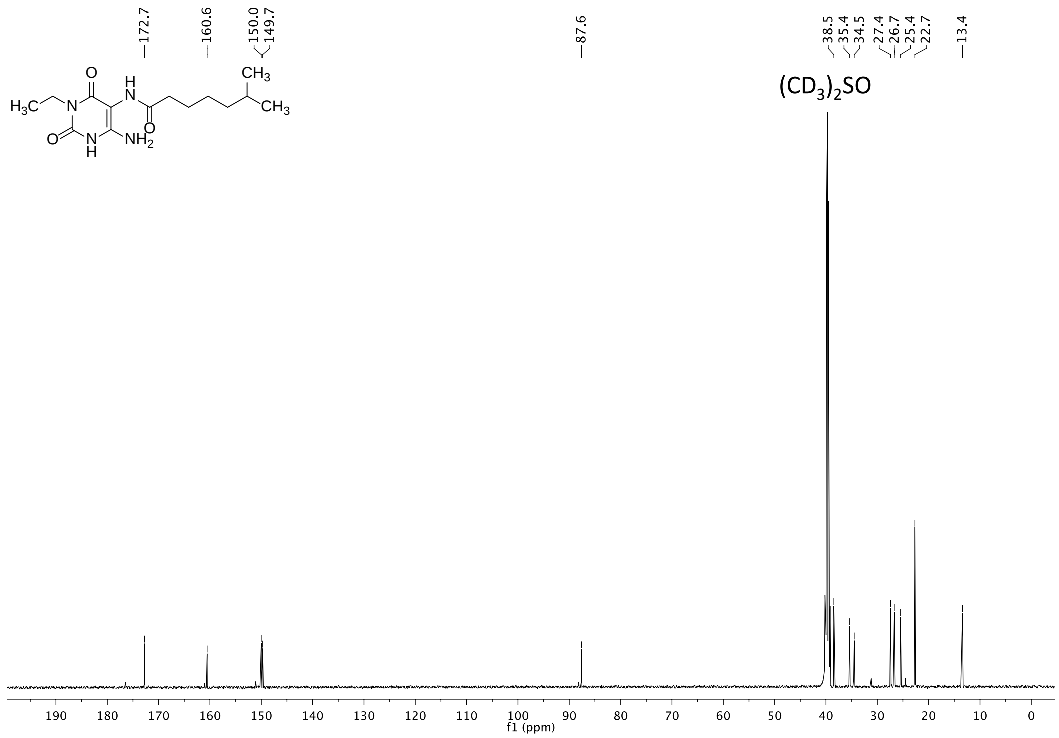

Figure S36. ^{13}C-NMR spectrum of 38 in $(CD_3)_2SO$ at room temperature.

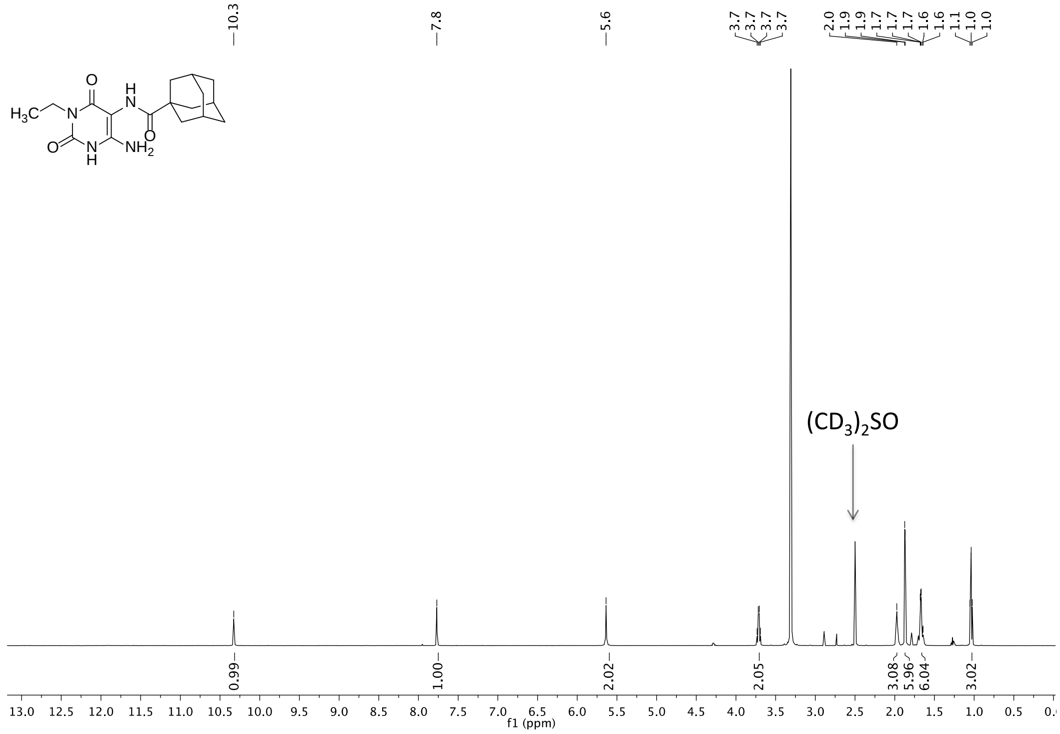

Figure S37. ^{1}H-NMR spectrum of 39 in $(CD_3)_2SO$ at room temperature.

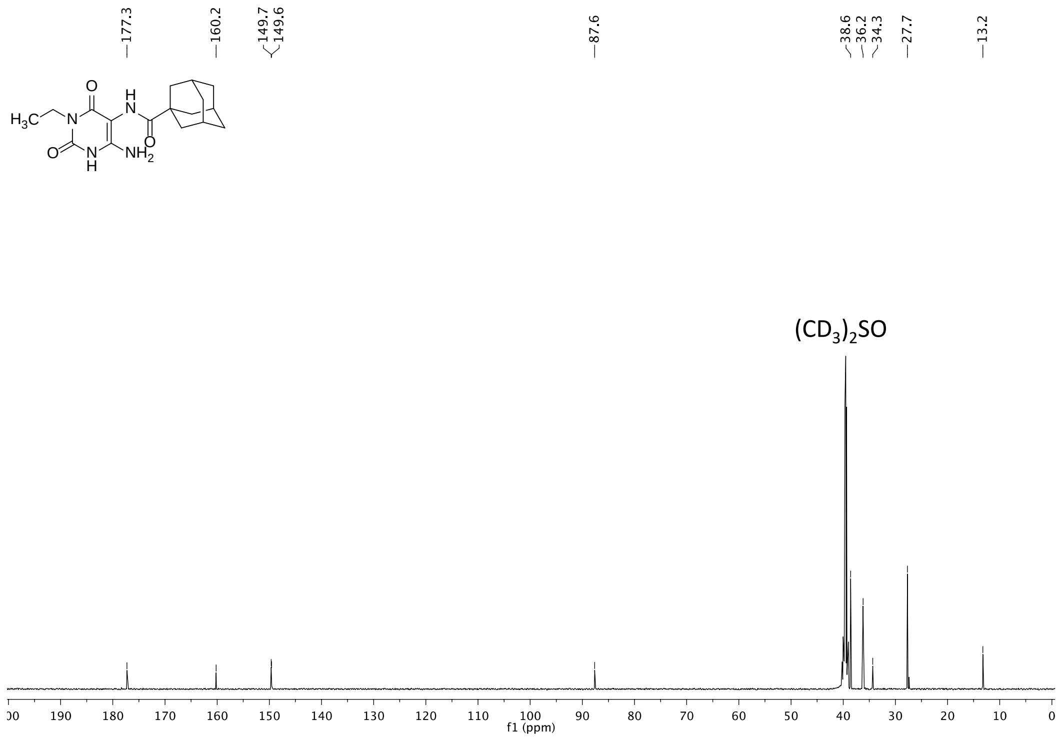

Figure S38. ^{13}**C-NMR spectrum of 39 in** $(CD_3)_2SO$ **at room temperature.**

IR-spectra of compounds 21-

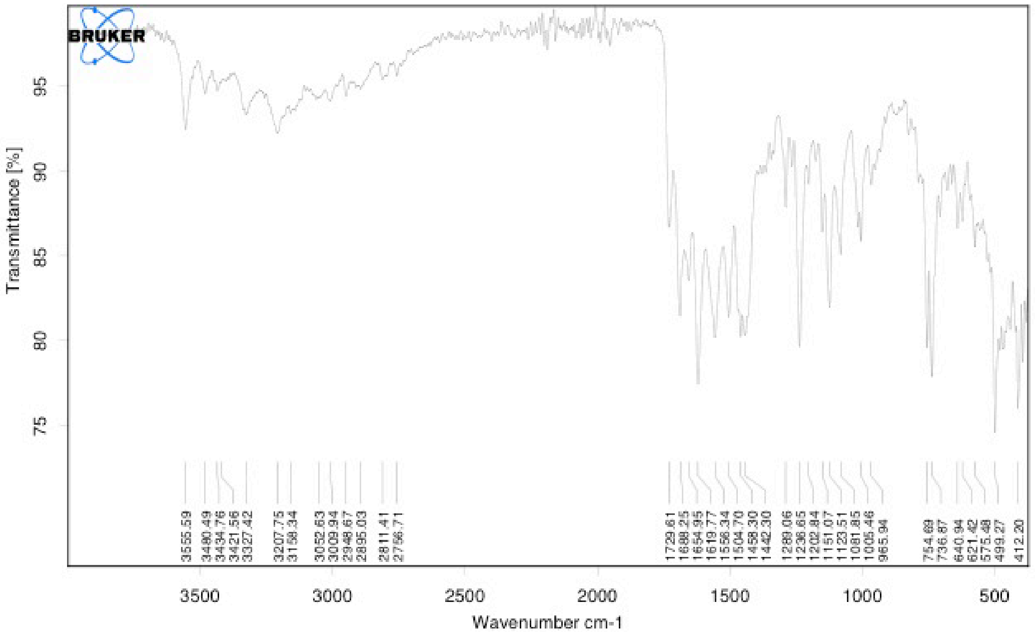

39

Figure S39. IR-spectrum of 21.

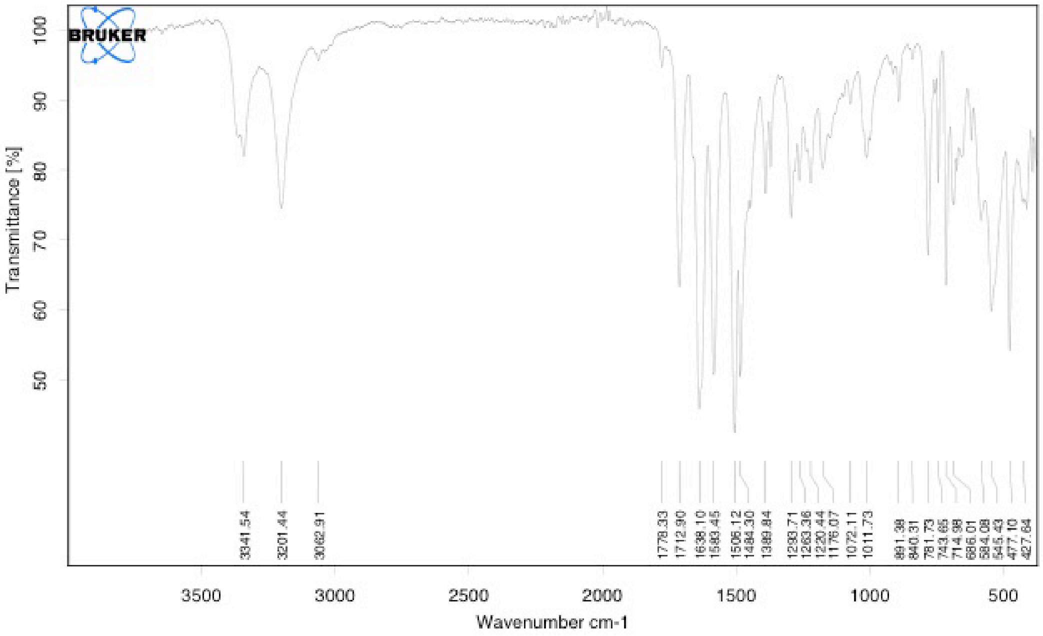

Figure S40. IR-spectrum of 22.

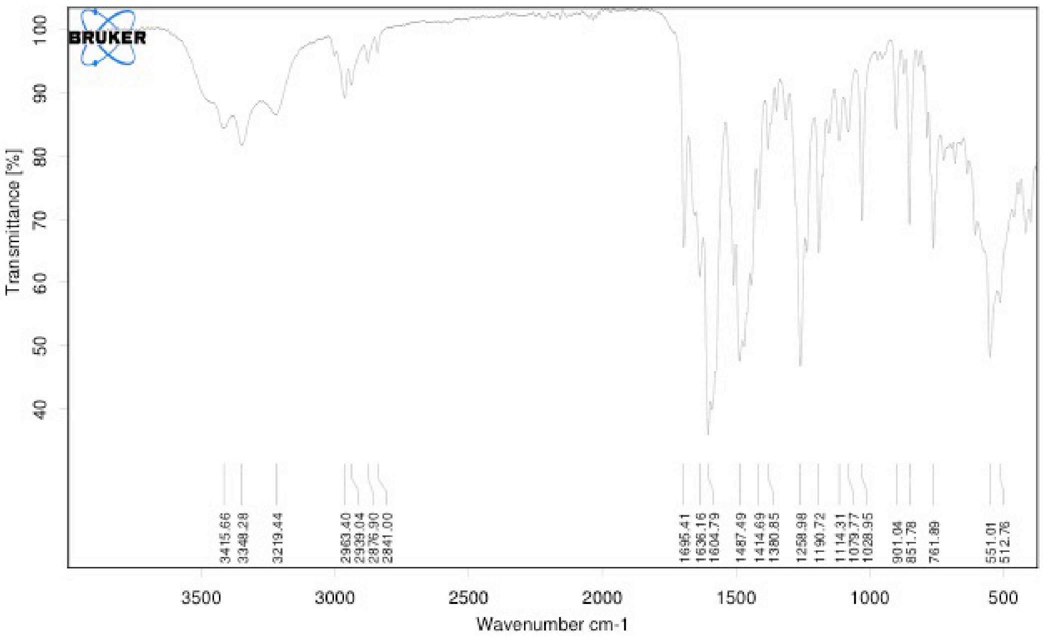

Figure S41. IR-spectrum of 23.

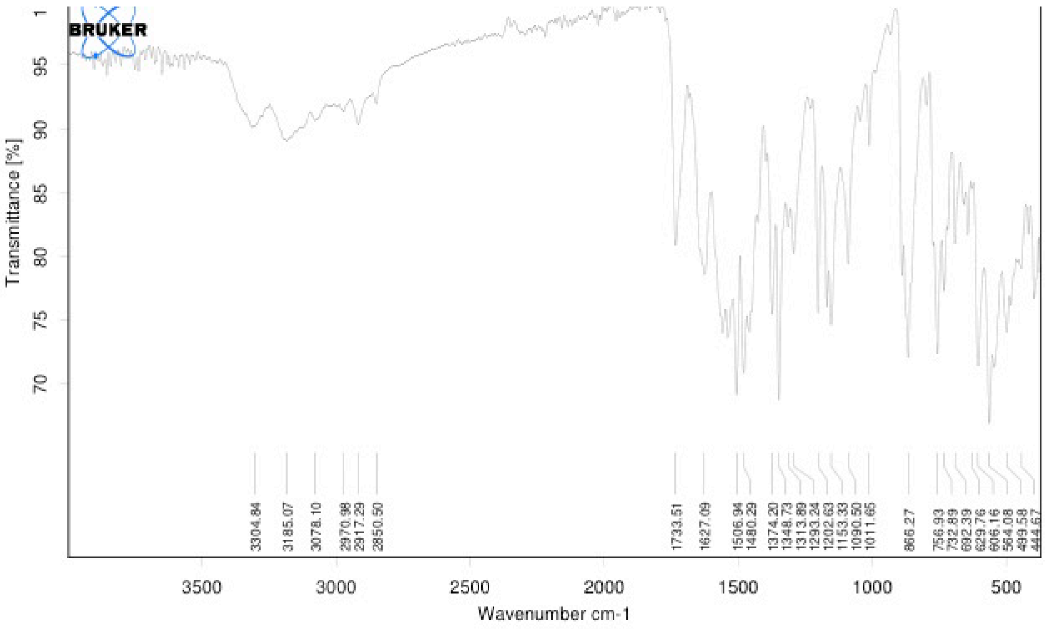

Figure S42. IR-spectrum of 24.

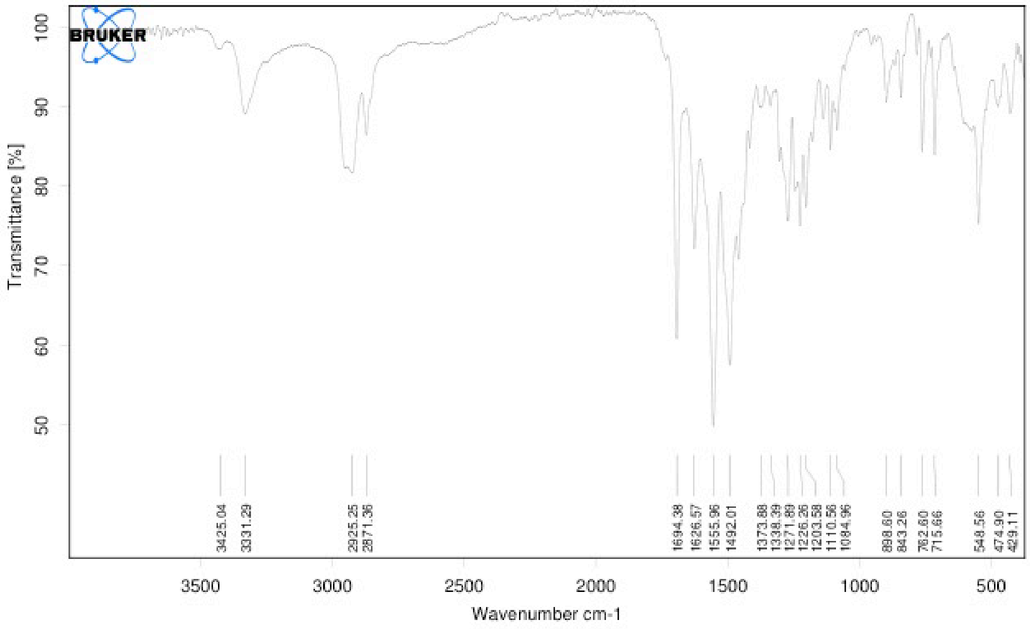

Figure S43. IR-spectrum of 25.

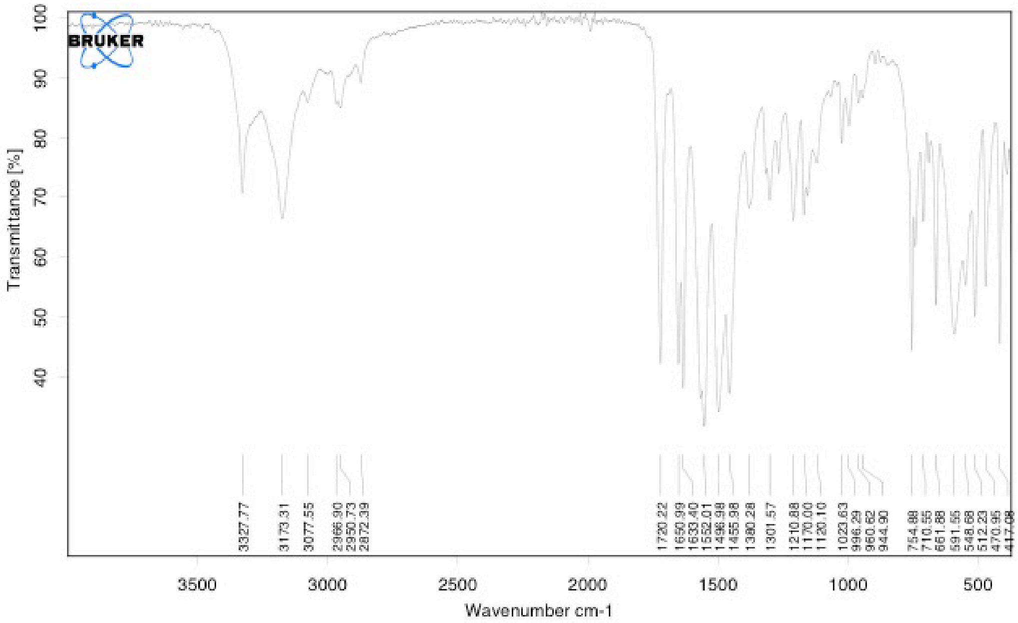

Figure S44. IR-spectrum of 26

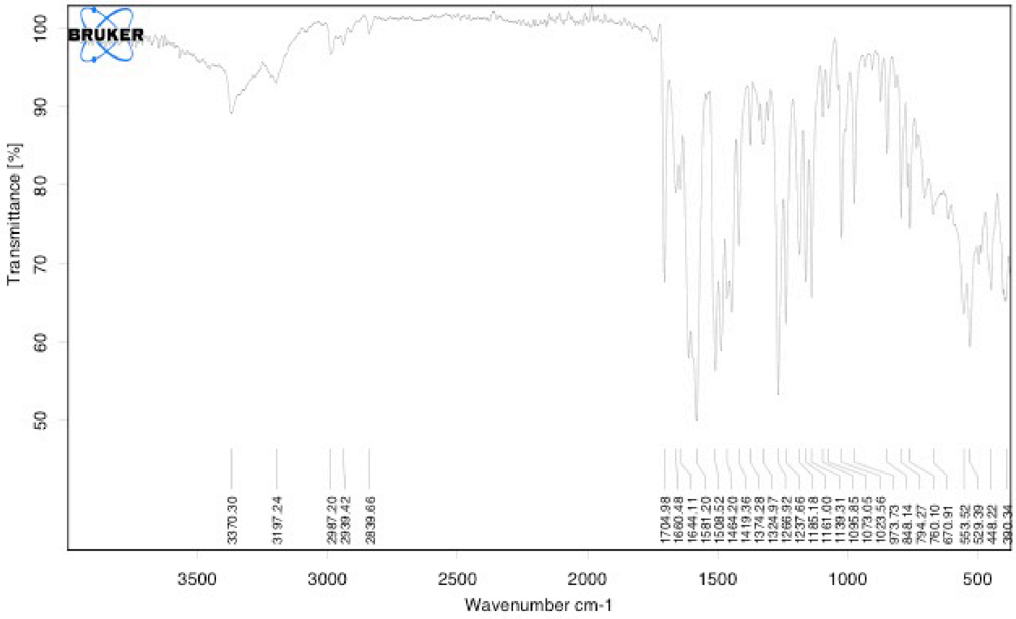

Figure S45. IR-spectrum of 27.

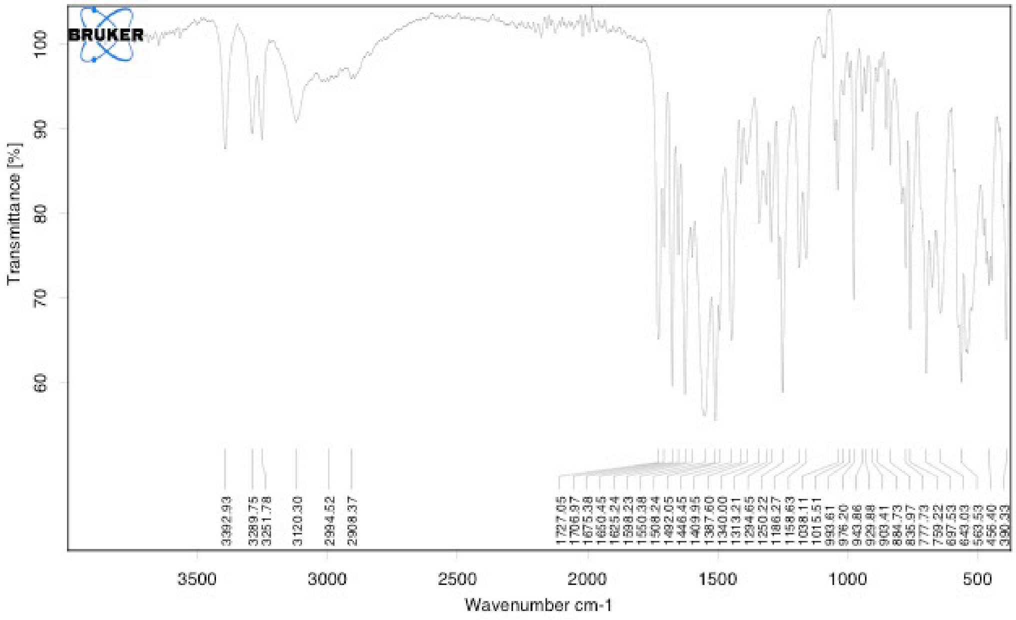

Figure S46. IR-spectrum of 28.

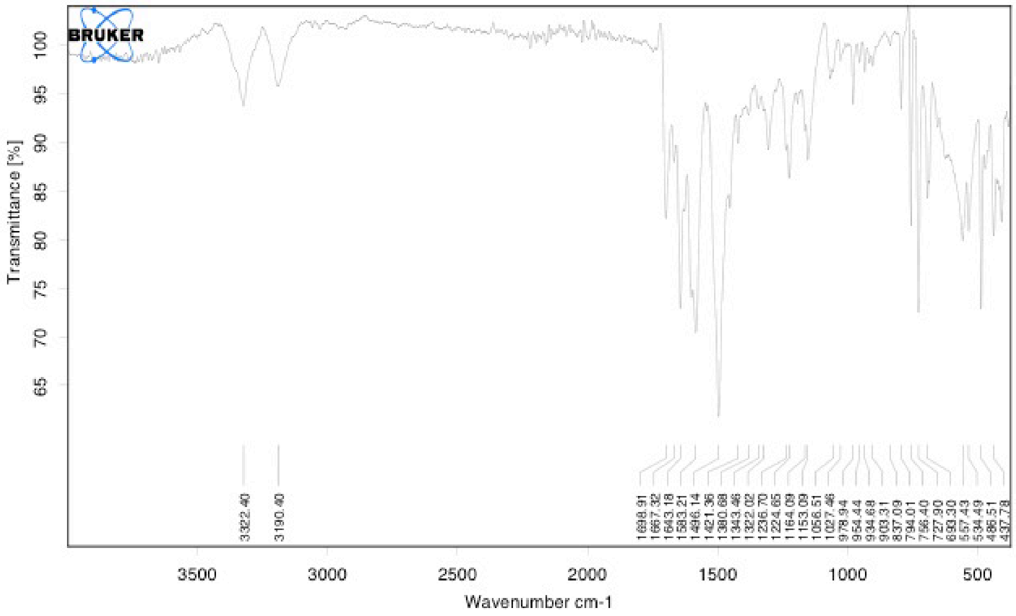

Figure S47. IR-spectrum of 29.

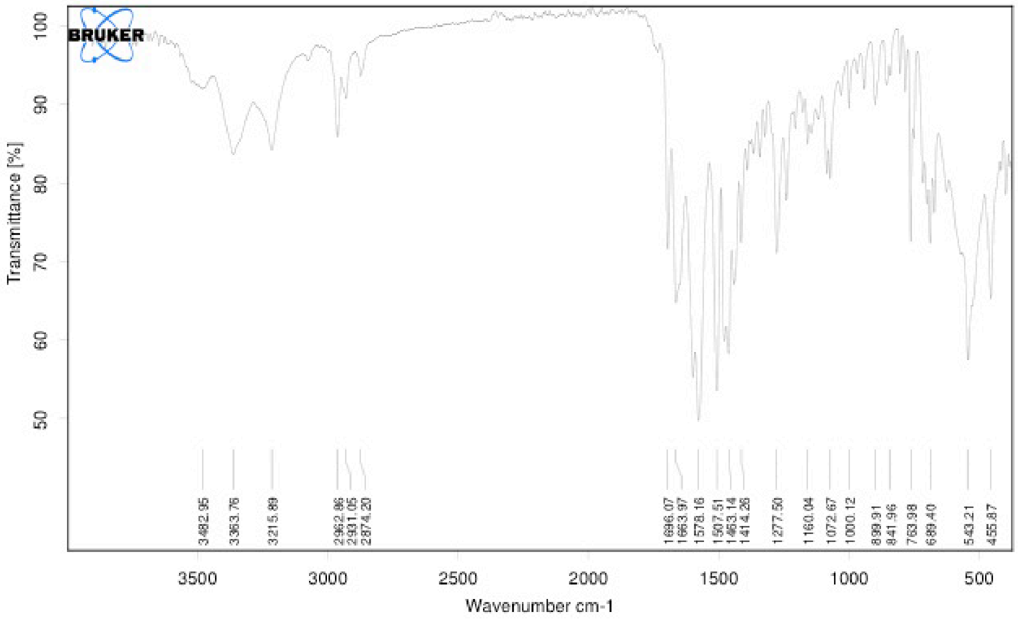

Figure S48. IR-spectrum of 30.

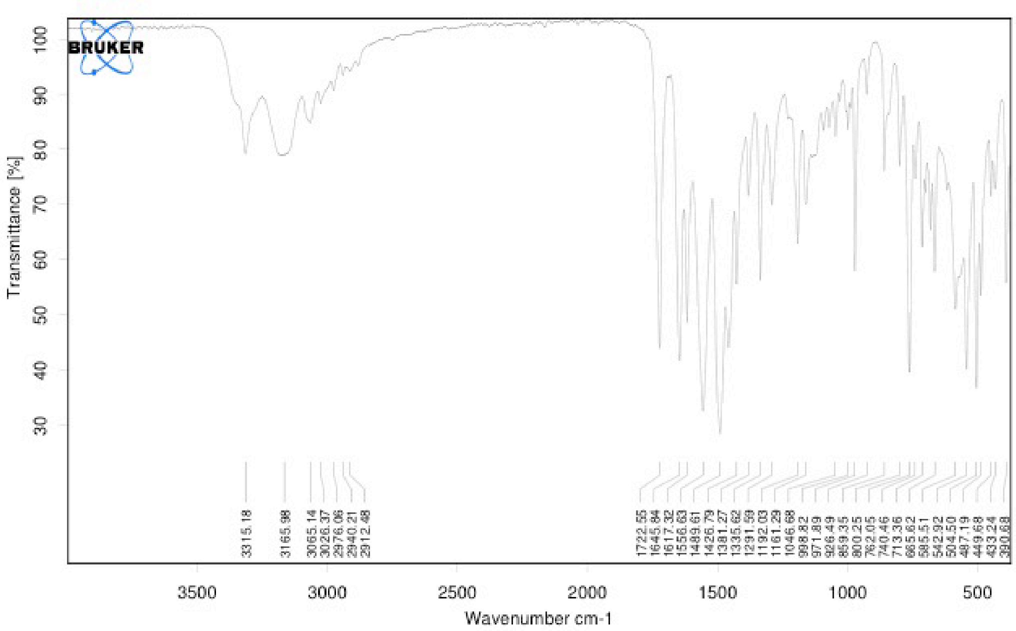

Figure S49. IR-spectrum of 31.

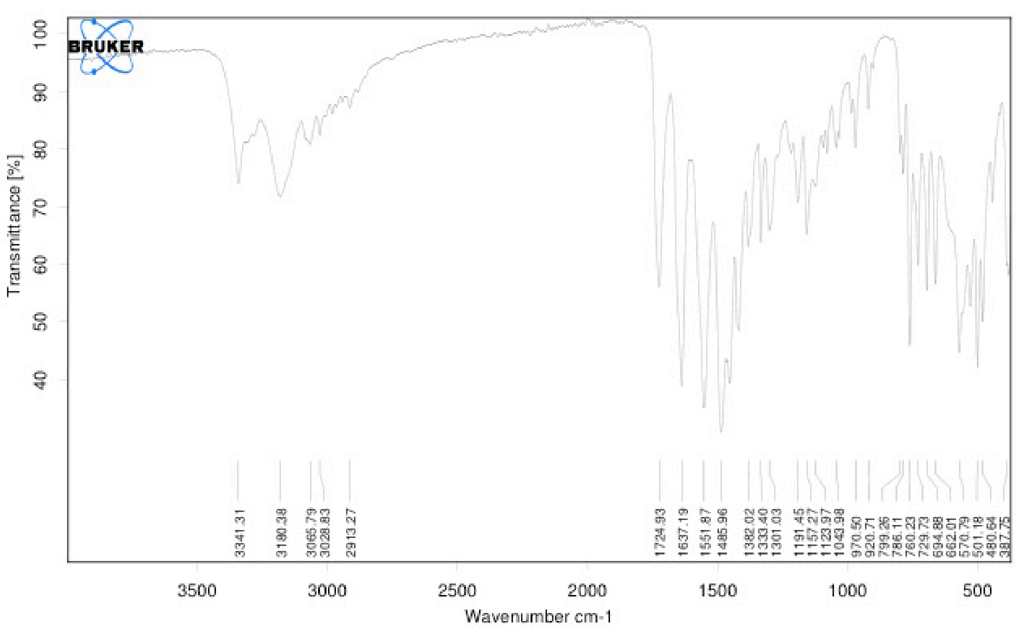

Figure S50. IR-spectrum of 32.

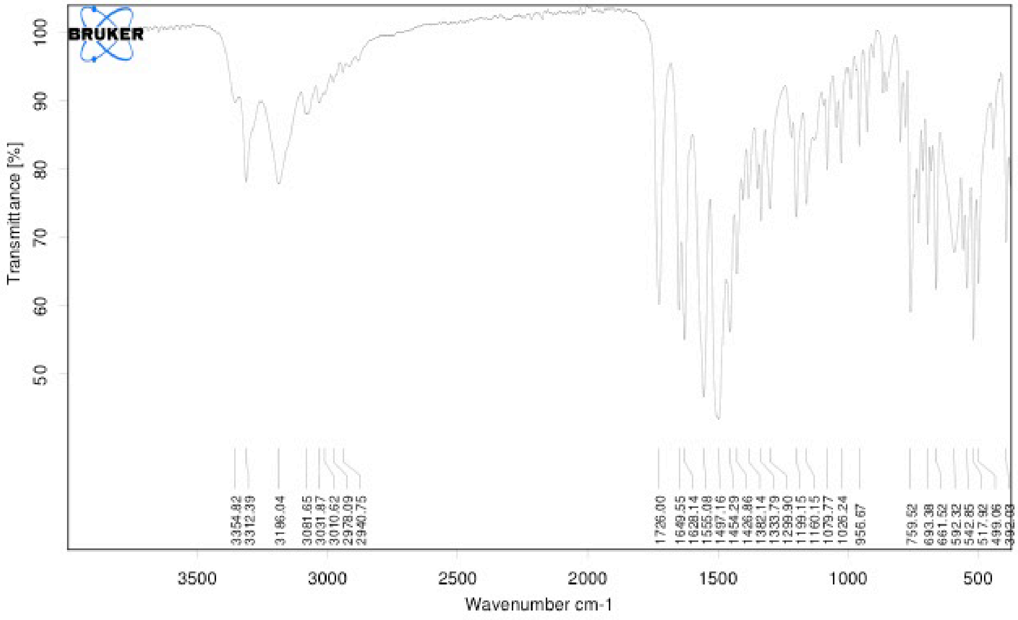

Figure S51. IR-spectrum of 33.

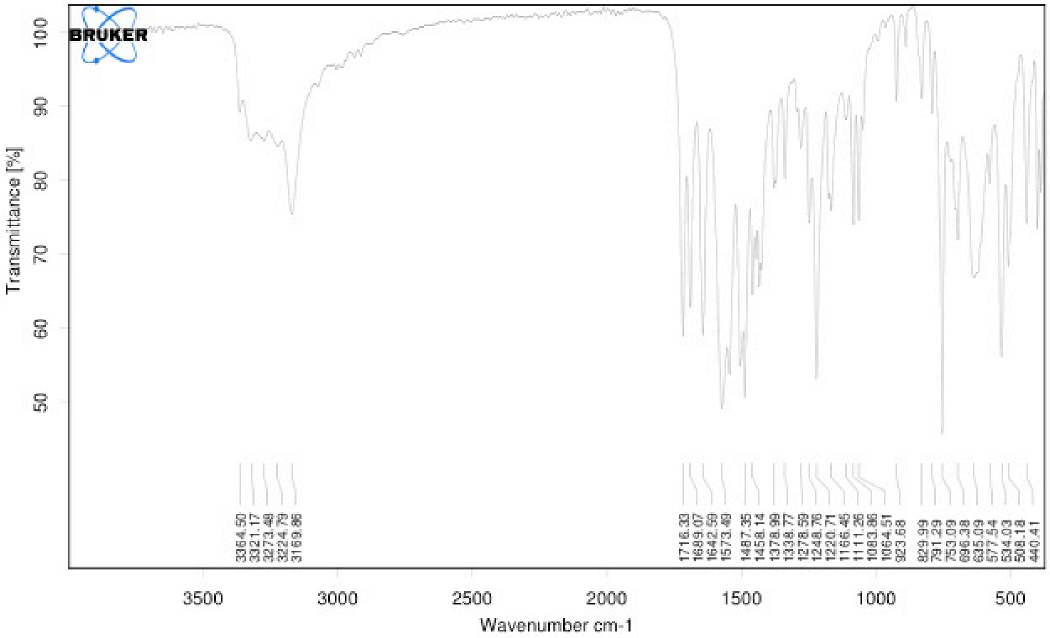

Figure S52. IR-spectrum of 34.

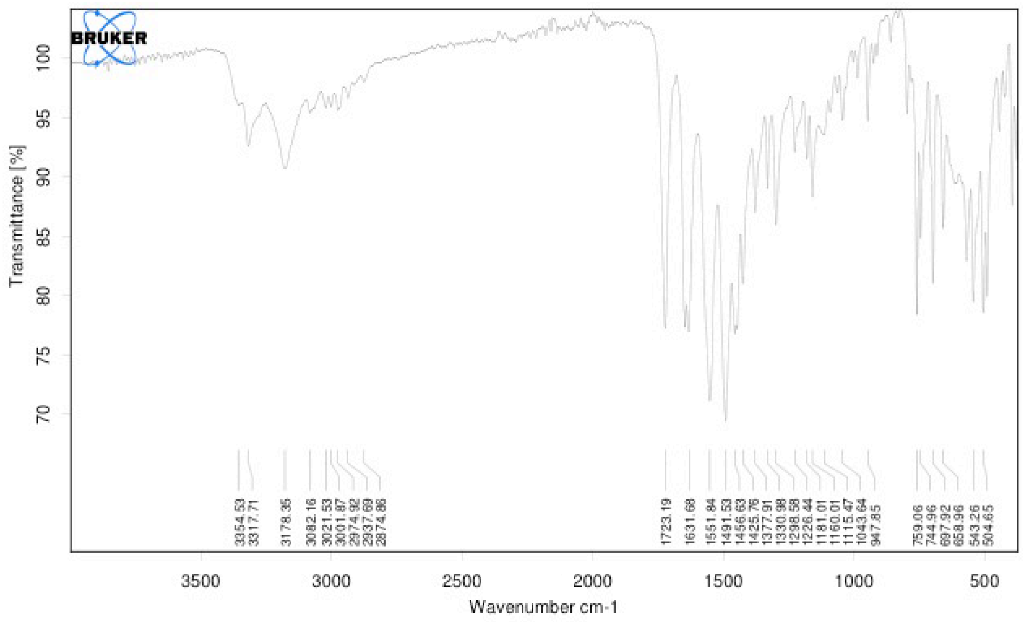

Figure S53. IR-spectrum of 35.

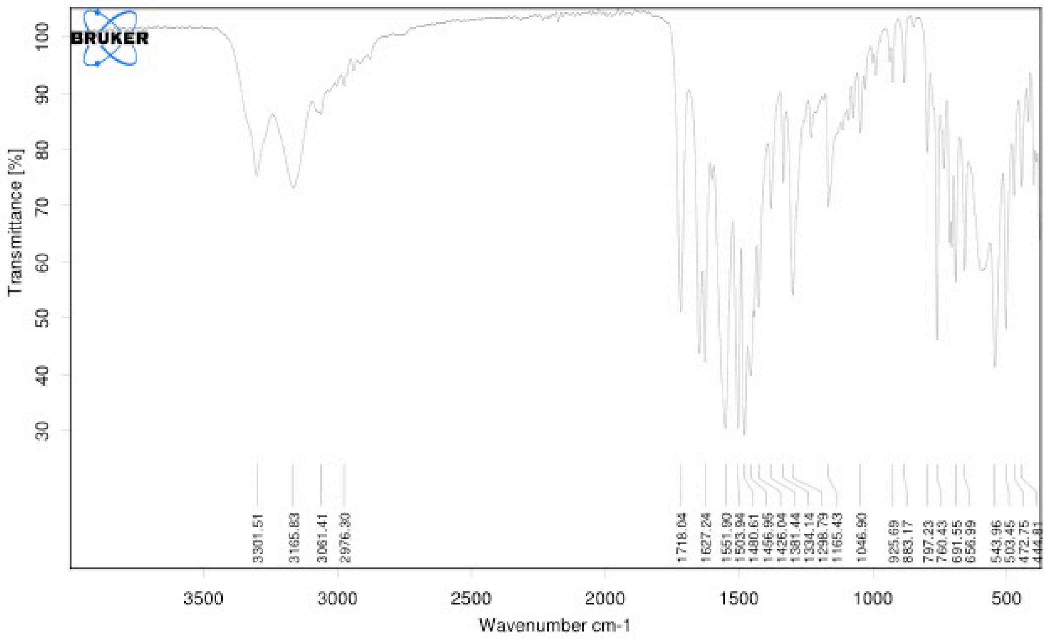

Figure S54. IR-spectrum of 36.

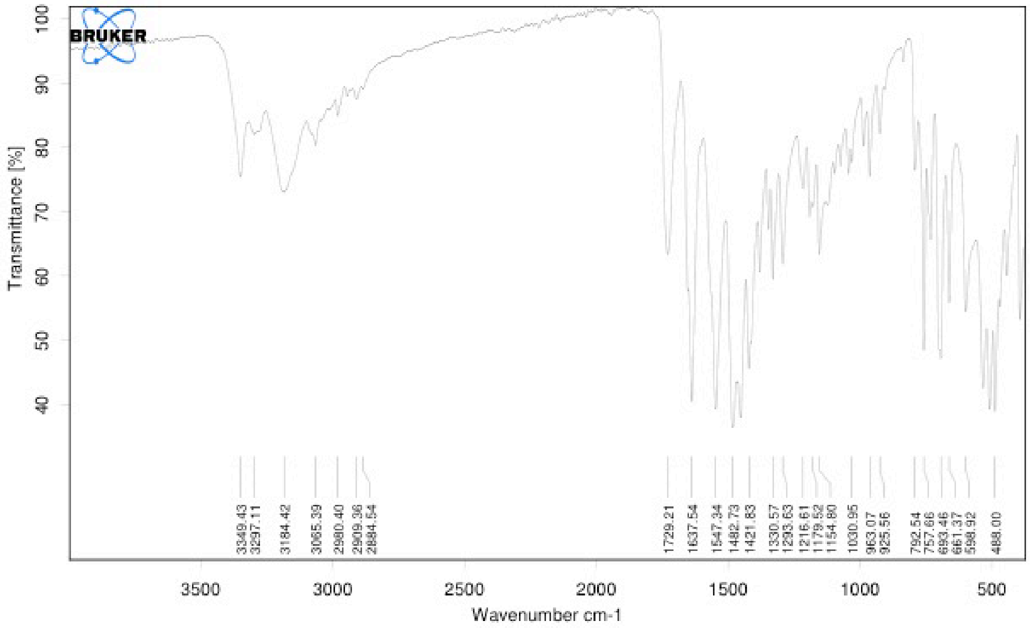

Figure S55. IR-spectrum of 37.

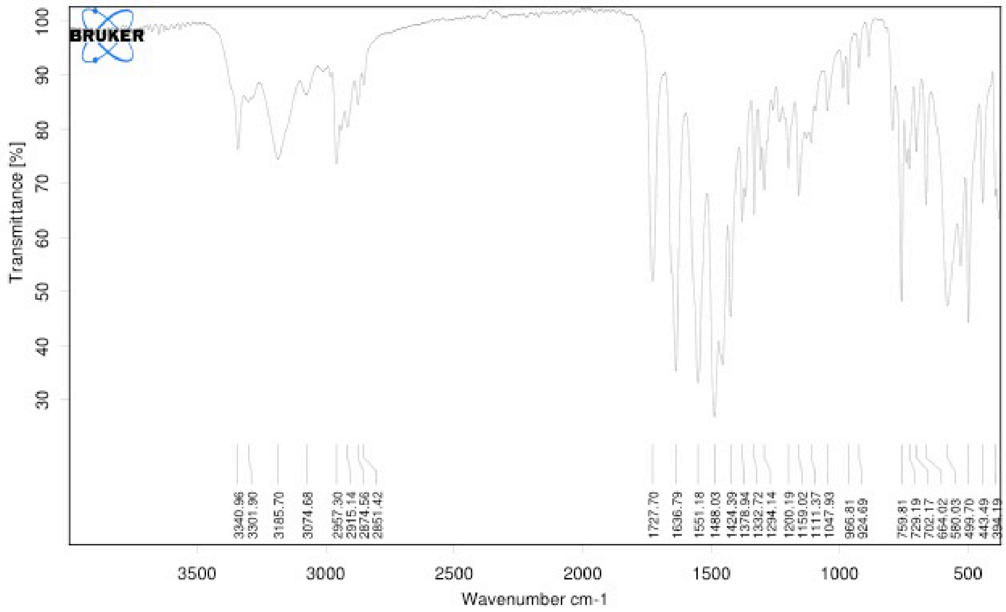

Figure S56. IR-spectrum of 38.

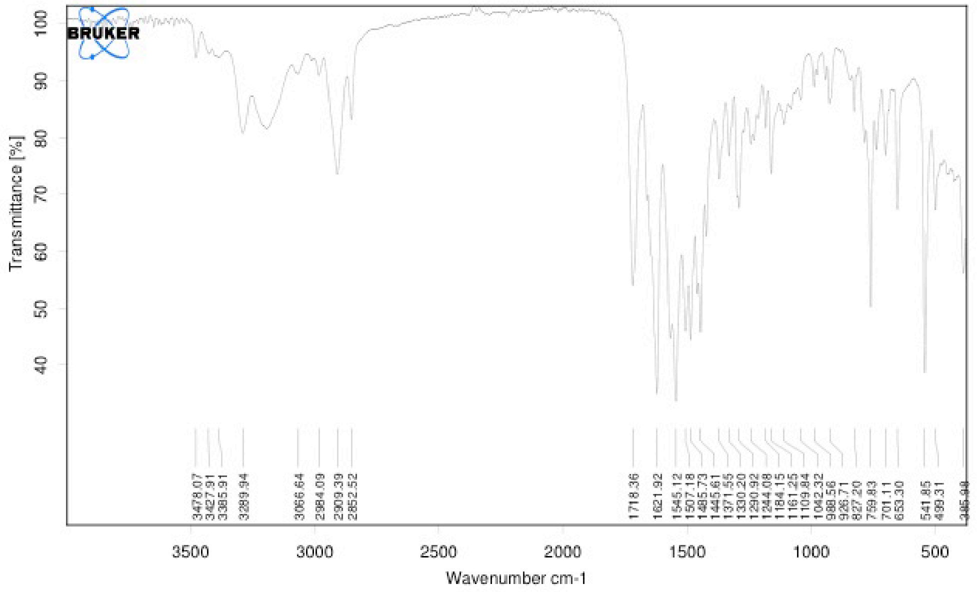

Figure S57. IR-spectrum of 39.

References

Hockemeyer, J., Burbiel, J.C., and Müller, C.E. (2004). Multigram-scale syntheses, stability, and photoreactions of A_{2A} adenosine receptor antagonists with 8-styrylxanthine structure: Potential drugs for Parkinson's disease. *J. Org. Chem.* 69, 3308-3318. doi: 10.1021/jo0358574.

Maxwell, L.C.E., and Salivar, C.J. (1952). Method of preparing 4-aminouracils. *US2715625A*.

Müller, C.E., Shi, D., Manning Jr, M., and Daly, J.W. (1993). Synthesis of paraxanthine analogs (1, 7-disubstituted xanthines) and other xanthines unsubstituted at the 3-position: Structure-activity relationships at adenosine receptors. *J. Med. Chem.* 36, 3341-3349.
